# Stage-specific associations of mineralization markers with CKM syndrome: Nationwide survey and genetic evidence for Alkaline phosphatase’s unique clinical role

**DOI:** 10.1371/journal.pone.0351946

**Published:** 2026-06-18

**Authors:** Lei Hua, Yongshou Tao, Liangru Shen

**Affiliations:** 1 Department of Cardiology, Affiliated Kunshan Hospital of Jiangsu University, Kunshan, Jiangsu, China; 2 Department of Cardiovascular Medicine, The First Affiliated Hospital of Nanjing Medical University, Nanjing, Jiangsu Province, China; 3 Baicheng County Maternal and Child Healthcare Hospital, Aksu, Xinjiang Province, China; 4 Department of Nephrology, Sihong Kang Shen Hospital, the Affiliated Hospital of Jiangsu Food & Pharmaceutical Science College, Suqian, Jiangsu Province, China; Hong Kong Baptist University, HONG KONG

## Abstract

**Background:**

Cardiovascular-Kidney-Metabolic (CKM) syndrome involves disordered mineralization processes. Herein, we investigated the serum alkaline phosphatase (ALP), calcium, and phosphorus across CKM stages.

**Methods:**

This study analyzed 15,233 eligible participants in National Health and Nutrition Examination Survey (2009–2018) using survey-weighted multinomial logistic regression to assess associations of ALP, calcium, and phosphorus. Restricted cubic splines assessed non-linear relationships, while Cox models examined mortality risks. Two-sample Mendelian randomization (MR) explored causal relationships.

**Results:**

Relative risk ratios (RRRs) and 95% confidence intervals (95% CI) were calculated with multinomial logistic regression. Compared with stage 0, ALP was positively associated with the likelihood of being classified into stage 2 (RRR, 1.23[95% CI, 1.11–1.37]), stage 3 (RRR, 1.99 [95% CI, 1.48–2.67]), stage 4a (RRR, 1.29 [95% CI, 1.14–1.46]), stage 4b (RRR, 1.35 [95% CI, 1.16–1.58]). Per mg/dL increase in serum calcium levels was associated with a higher likelihood of being classified into stages 2 (RRR = 2.13, 95% CI: 1.55–2.94) and CKM stage 4, while per mg/dL increase in serum phosphorus was associated with stage 3 classification (RRR = 2.85, 95% CI: 1.77–4.59) and the results remained consistent after standardizing the markers using z-scores. ALP’s 4th quartile was associated with the highest mortality risks (CKM-cause hazard ratio = 2.20; all-cause hazard ratio = 2.15). MR analysis indicated potential causal effects of ALP on cardiovascular disease and of chronic kidney disease on ALP.

**Conclusions:**

ALP demonstrates consistent associations with all CKM stages. These findings indicate that ALP-related mechanisms need further exploration.

## Introduction

Cardiovascular diseases (CVDs), chronic kidney diseases (CKDs), and metabolic disorders are among the leading causes of morbidity and mortality worldwide [1]. Along with growing recognition of their interconnected nature, the American Heart Association recently introduced the concept of Cardiovascular-Kidney-Metabolic (CKM) syndrome [2], which emphasizes the shared pathophysiological mechanisms underlying these conditions [3]. The global burden of CKM syndrome has been rising steadily from year 1990–2021 and is projected to continue increasing through year 2046 [4].

Physiological mineralization is a critical biological process for bone formation [5], tissue repair [6], dental development [7], and the maintenance of mineral homeostasis [5]. This process is largely driven by the formation of hydroxyapatite crystals through the interaction of calcium and phosphorus [8]. In the scenario of mineralization, alkaline phosphatase (ALP) is an enzyme that elevates the level of free phosphorus, promoting hydroxyapatite formation [9]. However, in the context of CKD, this strictly regulated balance of mineralization is disrupted. Impaired phosphate excretion leads to hyperphosphatemia, which in turn drives ectopic calcification, particularly within soft tissues such as blood vessels [10]. Elevated phosphate levels stimulate vascular smooth muscle cells (VSMCs) to undergo osteogenic transformation, promoting vascular calcification [11].

Beyond mineral metabolism disturbances, CKD [12], metabolic syndrome, and CVD [13,14] also trigger chronic inflammation and oxidative stress, further contributing to pathological calcification. Pro-inflammatory cytokines such as tumor necrosis factor-alpha (TNF-α) and interleukin-1 beta (IL-1β) activate osteogenic pathways in VSMCs [15], including the bone morphogenetic protein (BMP) and Wnt/β-catenin signaling cascades [16]. While experimental studies have demonstrated the role of ALP, calcium, and phosphate in vascular calcification and disease progression, findings from clinical studies examining their associations with CVDs, CKDs, and metabolic disorders remain inconsistent [17–19]. For example, calcium concentrations were positively associated with the mortality rate of CVD patients [20] while the reverse results were observed one year later [21]. Importantly, no prior research has comprehensively assessed how serum levels of ALP, calcium, and phosphate associate with different stages of CKM syndrome in a general population.

To address this gap, the present study aimed to evaluate the associations between these serum biomarkers and the risk of various CKM stages, both overall and across key subgroups. Both cross-sectional and mortality data from National Health and Nutrition Examination Survey (NHANES) was obtained, which is a database collecting health data and dietary information for adults and children in American using stratified, multistage probability sampling and national representative strategies [22]. Hence, we analyze this with survey-weighted logistic regression models, adjusting for relevant confounders, to examine these relationships. Additionally, restricted cubic spline (RCS) models were used to assess potential non-linear trends in the associations. Survival analysis were employed for evaluation relationship between ALP levels and CKM-cause and all-cause mortality.

## Materials and Methods

### Data sources

The datasets analyzed in this study were obtained from the NHANES database.

### Ethics approval and consent to participate

NHANES study protocol was permitted by the NCHS Research Ethics Review Board, and written informed consent was signed by participants once they were enrolled (the website is https://www.cdc.gov/nchs/nhanes/irba98.htm). Hence, Ethical approval and consent were not included in this study since the public data was obtained and re-analyzed.

### Study design and population

**Fig 1:**
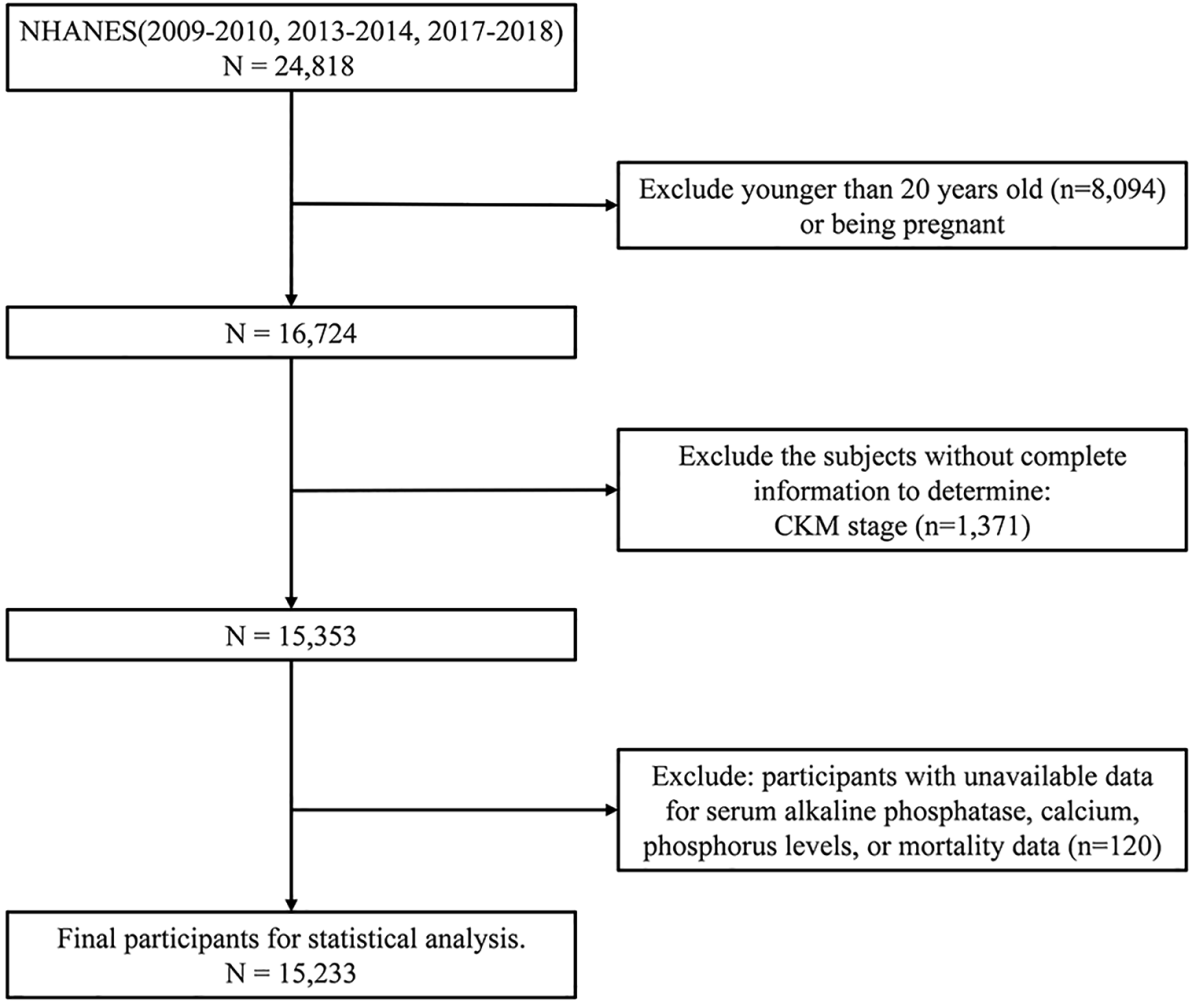
Schematic representation of the participant selection process.

Demographic and health data from the NHANES cycles 2009–2010, 2013–2014, and 2017–2018 were analyzed in this study. Participants under 20 years of age or those who were pregnant were excluded. Additionally, individuals with insufficient data to evaluate CKM staging were excluded—specifically, those missing information on major CVD events, estimated glomerular filtration rate (eGFR), diabetes status, body mass index (BMI), or cholesterol levels, since these are key parameters for determining CKM stage. Participants lacking serum ALP, calcium, or phosphorus measurements were also excluded to ensure the exposure data were available. A detailed description of CKM risk classification criteria and variable definitions is provided in S1Table. Briefly, 24,818 subjects were enrolled in three selected cycles and 8094 subjects were excluded due to the age being younger than 20 years old or being pregnant. The reason why we excluded participants aged below 20 years old instead of 18 years old was that some of the demographic data in the NHANES survey, such as and, was only available among participants above 20 years old to avoid the potential disclosure risks. Next, 1,434 and 5464 participants were ruled out because of without CKM data, serum ALP, phosphate, or calcium levels. Consequently, a total of 15,233 participants were included in the further study (Fig 1).

### Classification of the CKM syndrome

The classification of CKM stages (0-4b) mainly followed the definition of the American Heart Association [2] and was modified for NHANES data referring to the previous studies [2,4]. Briefly, participants were classified as stage 0 if they were neither overweight/obese (defined as BMI < 25 kg/m² and waist circumference <88 cm for women or <102 cm for men) nor had any clinical or subclinical CVD, renal impairment, or metabolic disorders (including hypercholesterolemia or diabetes); Stage 1 included individuals who were overweight or obese or had prediabetes; Stage 2 included participants with hypertension, diabetes, or moderate-to-high risk of renal impairment; Stage 3 comprised individuals with very high-risk renal impairment in the absence of CVD; Participants diagnosed with CVD were classified as stage 4, which was further subdivided into stage 4a (CVD with low-risk renal impairment) and stage 4b (CVD with moderate-to-very high-risk renal impairment). Detailed definitions of CVD, renal impairment, diabetes, CKM risk categories, and calculation methods are provided in S1Table. For ease of subsequent statistical analysis, CKM stages 0 and 1 were grouped as non-advanced CKM stages, while stages 2 through 4b were classified as advanced CKM stages in this study.

### Serum ALP, calcium, and phosphorus levels

Serum ALP, calcium, and phosphorus levels were under the standard biochemistry profile category, and they observed the same or similar sample storage and preparation protocols. In short, serum samples were separated and shortly used at room temperature for less than 8 hours or stored under −15 °C for longer than 48 hours. Collaborative Laboratory Services performed the detection, L.L.C company, using the Beckman Coulter UniCel® DxC800 Synchron Clinical System following its corresponding protocol. Results were recorded by Laboratory Information Systems and analyzed subsequently. Samples were diluted and re-measured once the concentration of the samples was higher than their respective analytical range. Further information about quality control and results can be reached via the NHANES database. For analytical purposes, serum ALP was evaluated both as a continuous variable and through quartile categorization. Using survey-weighted percentiles to account for NHANES’ complex sampling design, we established the following quartile ranges: Q1 (<54 IU/L, reference), Q2 (54–66 IU/L), Q3 (67–81 IU/L), and Q4 (>81 IU/L). The reason was that the relative risk ratios (RRRs) for ALP were pretty close to the margin for each unit change in ALP. Calcium and phosphorus levels were only analyzed as continuous variables (mg/dL) through all the models.

### Potential confounders

Confounders included in this study were selected based on prior research [23] and adapted to the current dataset. These variables included age (years), sex, race/ethnicity, smoking status, poverty income ratio (PIR), education level, body mass index (BMI, kg/m²), and serum vitamin D (nmol/L). For subgroup analyses, age was categorized into three groups: young adults (20–44 years), middle-aged adults (45–64 years), and older adults (≥65 years). Sex (male or female) was treated as a categorical variable. Race/ethnicity was classified as Mexican American, Other Hispanic, Non-Hispanic White, Non-Hispanic Black, and Other/multiracial. Education level was categorized as less than high school, high school graduate, some college, and college or above. PIR was grouped as poor (PIR < 2), near poor (2 ≤ PIR < 5), and not poor (PIR ≥ 5). BMI categories were defined as underweight (BMI < 18.5 kg/m²), normal weight (18.5 ≤ BMI < 25 kg/m²), overweight (25 ≤ BMI < 30 kg/m²), and obese (BMI ≥ 30 kg/m²). Information on age, sex, race/ethnicity, PIR, and education was obtained via questionnaire and stored in the NHANES demographic files. Serum total vitamin D (25-hydroxyvitamin D2 + D3) was used rather than the individual subtypes, as total vitamin D has been reported to be associated with calcium and phosphorus levels [24]. Smoking status was defined as follows: current smokers were individuals who currently smoke; former smokers had smoked more than 100 cigarettes in their lifetime but had quit; and never smokers had smoked fewer than 100 cigarettes and had quit.

### Survival analysis

Mortality data from three NHANES cycles (2009–2010, 2013–2014, and 2017–2018) were collected and combined for survival analysis. In NHANES, follow-up time was defined as the period from the baseline interview until death or the end of the study. CKM-cause mortality in this study was defined as death due to diseases of the heart, renal diseases (including nephritis, nephrotic syndrome, and nephrosis), or diabetes mellitus. Diabetes mellitus was considered the primary metabolism-related cause of death based on previous research [25]. Initially, Kaplan–Meier analysis was conducted to assess the associations between ALP quartiles and CKM-cause as well as all-cause mortality, with significance among quartiles evaluated using the log-rank test. Subsequently, survey-weighted Cox proportional hazards models were applied to estimate the associations of serum ALP quartiles, calcium, and phosphorus levels with CKM-cause and all-cause mortality, adjusting for the same covariates used in the survey-weighted multinomial logistic regression models.

### Mendelian randomization (MR) analysis to infer causality between ALP and key components of CKM syndrome

S1 and S2 Figs illustrate the flowcharts for the two-sample MR analyses. Summary statistics for serum ALP, based on 437,896 participants, were obtained from the UK Biobank. Corresponding genome-wide association study (GWAS) summary statistics for CKD (212,841 controls and 3,902 cases), CVD (131,686 controls and 15,526 cases), and diabetes (170,213 controls and 21,284 cases) were retrieved from the FinnGen database (release 12). Detailed descriptions of these datasets are provided in S2Table. All summary statistics used in this study were derived from individuals of European ancestry.

To identify genetic instruments for the exposure, single nucleotide polymorphisms (SNPs) were initially filtered using a genome-wide significance threshold of *P*  <  5  ×  10 ⁻ ⁸ to ensure strong associations with serum ALP. To minimize linkage disequilibrium (LD) bias, SNPs with *r²*  ≥  0.001 were excluded. After this initial filtering, 399 SNPs associated with ALP were retained. Next, the LDlink tool (https://ldlink.nih.gov/?tab=home), developed by the National Institutes of Health, was used to further exclude SNPs associated with known confounders (e.g., BMI, smoking status, education level) or outcomes (e.g., CVD, diabetes). As a result, 322 SNPs were removed. Detailed exclusion reasons are presented in S3Table, and 77 selected SNPs were adopted for further analysis, and their mean *F*-statistic was 87.53 (ranging from 28.24 to 589.78). After harmonization and additional LD pruning with the outcome datasets, 60 and 61 SNPs were selected as instrumental variables for the MR analyses of ALP on CVD and diabetes, respectively. A similar instrument selection process was conducted for the reverse two-sample MR analysis investigating CKD as the exposure and ALP as the outcome, yielding 90 SNPs for MR analysis.

### Statistical analysis

NHANES employs a multistage, stratified sampling design; therefore, all statistical analyses in this study were adjusted using survey weights to account for the complex sampling strategy. Categorical variables were summarized as weighted frequencies (percentages), while continuous variables were described using weighted means and standard errors (SEs). To assess differences across groups, survey-weighted chi-square (χ²) tests were used for categorical variables, and survey-weighted linear regression was applied for continuous variables. These comparisons were conducted across both individual CKM stages (0–4b) and combined CKM stage categories.

For the main comparisons, survey-designed multinomial logistic regression was performed to estimate the RRRs of serum ALP, calcium, and phosphorus levels across the CKM stages 0-4b adjusted with the proper weights according to the NHANES tutorials [26]. Weight data from the mobile examination center were adopted because dietary data were not included in the current study. Multinomial logistic regression was chosen instead of ordinal logistic regression because ordinal logistic regression assumes that the severity of outcomes is equally distributed, but CKM stages 0-4b were not proportional classified in the current study. Furthermore, survey-weighted binomial logistic regression was used to evaluate the odds ratios of ALP quartiles, calcium, and phosphorus levels in the models distinguishing advanced CKM stages from non-advanced CKM stages, since the outcomes for the models were binary (non-advanced versus advanced CKM stages). To improve comparability, regression analyses were also performed with all three markers standardized as z-scores. Both survey-weighted multinomial and binomial logistic regressions had the adjustment for age (years), BMI (kg/m²), sex, race and ethnicity, PIR, vitamin D (nmol/L), education, and smoking status. Moreover, to validate the stability of the conclusions among different populations, subgroup analysis was performed with the aforementioned adjustment. Besides, the RCS analysis functioned to determine if relationships were linear between the three serum markers and the ORs of the advanced CKM stages, with the adjustment.

Except for bone ALP, liver ALP is another source abundant in serum. We therefore performed the sensitivity analysis by excluding all the participants with a self-report of, adjusting for all the variables aforementioned. Additionally, to further exclude the potential confounding effects of the dysfunctional liver, serum alanine transaminase (ALT) and aspartate aminotransferase (AST) were additionally adjusted. To minimize potential confounding related to mineral metabolism, vitamin D supplement use and phosphate binder medication use were further included as covariates in the adjusted models.

Since serum albumin can bind with calcium and mask the changes of the ionized calcium levels among critically ill participants, albumin-corrected calcium levels were calculated using the formula: albumin-corrected serum calcium (mg/dL) = serum total calcium (mg/dL) + 0.8 * (4(g/dL) – serum albumin (g/dL)) [27]. Survey-designed multinomial and binomial logistic regressions were performed to evaluate the associations of albumin-corrected calcium with CKM stages and Cox proportional hazards models were applied to investigate its association with CKM-cause and all-cause mortality.

Additionally, two-sample MR analyses were performed using the TwoSampleMR package (https://github.com/MRCIEU/TwoSampleMR). The RadialMR package [28] was used to identify and remove potential outlier SNPs. Causal effects were primarily estimated using the inverse variance weighted (IVW) method, with results from the MR-Egger method reported for comparison. To evaluate potential sources of bias, heterogeneity was assessed using Cochran’s Q test, and horizontal pleiotropy was examined via the MR-Egger intercept test.

R (version 4.4.2) was the main tool used for data collection, cleaning, and statistical analysis, except for survey-weighted multinomial logistic regression, since R could not combine the weight adjustment with multinomial logistic regression. Hence, this was performed with STATA (version 17.0, StataCorp LLC). The sample size remained large (15,233) after excluding the participants with missing necessary data. Thus, imputations were not executed during the analysis. Two-sided *p*  <  0.05 was set as the threshold for statistical significance.

## Results

### Participants and demographic characteristics

Of 15,233 eligible participants, their mean age was 49.9 years old and male participants accounted for 48.6%. Table 1 presents the baseline characteristics of the participants grouped by CKM stages 0-4b. The percentage of the missing confounders varied from 3.5% to 9.7%. Higher stages of CKM tended to appear among older age, male, former or current smokers, lower PIR, and lower educational level participants. Additionally, compared to stage 0, participants in stages 2-4b showed a significantly higher serum vitamin D level. The same demographic characteristics were found in advanced CKM stages compared to non-advanced stages (Table 2).

**Table 1:** Demographic and clinical characteristics of participants among CKM syndrome stages 0-4b.

Characteristics	Stage 0 (N = 1746)	Stage 1 (N = 3501)	Stage 2 (N = 8270)	Stage 3 (N = 186)	Stage 4a (N = 857)	Stage 4b (N = 673)	*p*
**Age (years)**	34.1 ± 12.2	39.0 ± 13.4	54.5 ± 15.7	67.5 ± 12.5	62.8 ± 13.1	70.7 ± 10.2	***<0.001***
**Age group**							
20-44	1408 (80.6%)	2421 (69.2%)	2323 (28.1%)	15 (8.1%)	81 (9.5%)	15 (2.2%)	***<0.001***
45-64	300 (17.2%)	920 (26.3%)	3572 (43.2%)	54 (29%)	358 (41.8%)	144 (21.4%)	
>=65	38 (2.2%)	160 (4.6%)	2375 (28.7%)	117 (62.9%)	418 (48.8%)	514 (76.4%)	
**Sex**							
Male	738 (42.3%)	1593 (45.5%)	4085 (49.4%)	87 (46.8%)	522 (60.9%)	383 (56.9%)	***<0.001***
Female	1008 (57.7%)	1908 (54.5%)	4185 (50.6%)	99 (53.2%)	335 (39.1%)	290 (43.1%)	
**Race and ethnicity**							
Mexican American	178 (10.2%)	765 (21.9%)	1227 (14.8%)	29 (15.6%)	92 (10.7%)	53 (7.9%)	***<0.001***
Other Hispanic	158 (9%)	419 (12%)	778 (9.4%)	16 (8.6%)	60 (7%)	33 (4.9%)	
Non-Hispanic White	818 (46.8%)	1318 (37.6%)	3469 (41.9%)	68 (36.6%)	472 (55.1%)	373 (55.4%)	
Non-Hispanic Black	225 (12.9%)	611 (17.5%)	1752 (21.2%)	54 (29%)	160 (18.7%)	165 (24.5%)	
Other/multiracial	367 (21%)	388 (11.1%)	1044 (12.6%)	19 (10.2%)	73 (8.5%)	49 (7.3%)	
**CVD status**							
No	1746 (100%)	3501 (100%)	8270 (100%)	186 (100%)	0 (0%)	0 (0%)	***--***
ASCVD	0 (0%)	0 (0%)	0 (0%)	0 (0%)	666 (77.7%)	415 (61.7%)	
HF	0 (0%)	0 (0%)	0 (0%)	0 (0%)	191 (22.3%)	258 (38.3%)	
**Hypertension**							
Low risk	1746 (100%)	3501 (100%)	1648 (19.9%)	17 (9.1%)	188 (21.9%)	81 (12%)	***--***
Hypertension	0 (0%)	0 (0%)	6622 (80.1%)	169 (90.9%)	669 (78.1%)	592 (88%)	
**Renal impairment**							
Low risk	1746 (100%)	3501 (100%)	6414 (77.6%)	0 (0%)	857 (100%)	0 (0%)	***--***
Moderate risk	0 (0%)	0 (0%)	1492 (18%)	0 (0%)	0 (0%)	357 (53%)	
High risk	0 (0%)	0 (0%)	364 (4.4%)	0 (0%)	0 (0%)	159 (23.6%)	
Very high risk	0 (0%)	0 (0%)	0 (0%)	186 (100%)	0 (0%)	157 (23.3%)	
**Diabetes status**							
No diabetes	1746 (100%)	2440 (69.7%)	3767 (45.6%)	40 (21.5%)	264 (30.8%)	152 (22.6%)	***--***
Prediabetes	0 (0%)	1061 (30.3%)	3073 (37.2%)	57 (30.6%)	393 (45.9%)	269 (40%)	
Undiagnosed diabetes	0 (0%)	0 (0%)	409 (4.9%)	9 (4.8%)	36 (4.2%)	36 (5.3%)	
Diagnosed diabetes	0 (0%)	0 (0%)	1021 (12.3%)	80 (43%)	164 (19.1%)	216 (32.1%)	
**Cholesterol (mg/dL)**							
Normal	1746 (100%)	3501 (100%)	4679 (56.6%)	87 (46.8%)	365 (42.6%)	266 (39.5%)	***--***
High cholesterol	0 (0%)	0 (0%)	3591 (43.4%)	99 (53.2%)	492 (57.4%)	407 (60.5%)	
**Smoking status**							
Never smoke	1097 (62.8%)	2178 (62.2%)	4548 (55%)	92 (49.5%)	327 (38.2%)	277 (41.2%)	***<0.001***
Former smoker	242 (13.9%)	605 (17.3%)	2128 (25.7%)	72 (38.7%)	325 (37.9%)	276 (41%)	
Current smoker	407 (23.3%)	718 (20.5%)	1594 (19.3%)	22 (11.8%)	205 (23.9%)	120 (17.8%)	
**Poverty income ratio (PIR)**							
Not poor	359 (22.5%)	534 (17.1%)	1373 (18.4%)	10 (6.1%)	107 (13.7%)	75 (12.1%)	***<0.001***
Near poor	534 (33.5%)	1020 (32.7%)	2652 (35.6%)	50 (30.3%)	225 (28.7%)	211 (33.9%)	
Poor	703 (44%)	1567 (50.2%)	3433 (46%)	105 (63.6%)	451 (57.6%)	336 (54%)	
**Education**							
Less than high school	266 (15.2%)	804 (23%)	1962 (23.7%)	63 (33.9%)	263 (30.7%)	207 (30.8%)	***<0.001***
High school	365 (20.9%)	747 (21.3%)	1950 (23.6%)	45 (24.2%)	209 (24.4%)	181 (26.9%)	
Some college	527 (30.2%)	1107 (31.6%)	2524 (30.5%)	57 (30.6%)	248 (28.9%)	178 (26.4%)	
College above	588 (33.7%)	843 (24.1%)	1834 (22.2%)	21 (11.3%)	137 (16%)	107 (15.9%)	
**BMI (kg/m²)**	21.8 ± 2.0	30.1 ± 5.7	30.3 ± 7.2	30.7 ± 8.0	30.5 ± 7.6	30.8 ± 7.2	***---***
**Phosphorus (mg/dL)**	3.8 ± 0.5	3.7 ± 0.6	3.7 ± 0.6	4.0 ± 0.8	3.7 ± 0.5	3.8 ± 0.6	***<0.001***
**Calcium (mg/dL)**	9.4 ± 0.3	9.3 ± 0.3	9.4 ± 0.4	9.4 ± 0.6	9.4 ± 0.4	9.4 ± 0.4	***<0.001***
**ALP (IU/L)**	60.7 ± 19.2	68.0 ± 20.6	73.5 ± 25.0	86.0 ± 33.8	76.2 ± 27.5	79.8 ± 34.5	***<0.001***
**ALP Quartile**							
1^st^ Quartile	756 (43.3%)	985 (28.1%)	1747 (21.1%)	27 (14.5%)	167 (19.5%)	136 (20.2%)	***<0.001***
2^nd^ Quartile	468 (26.8%)	938 (26.8%)	2035 (24.6%)	33 (17.7%)	192 (22.4%)	139 (20.7%)	
3^rd^ Quartile	323 (18.5%)	897 (25.6%)	2179 (26.3%)	38 (20.4%)	221 (25.8%)	150 (22.3%)	
4rtile	199 (11.4%)	681 (19.5%)	2309 (27.9%)	88 (47.3%)	277 (32.3%)	248 (36.8%)	
**Albumin (g/dL)**	4.36 ± 0.32	4.20 ± 0.32	4.18 ± 0.33	3.88 ± 0.41	4.11 ± 0.32	4.03 ± 0.35	***<0.001***
**Vitamin D (nmol/L)**	65.6 ± 26.1	59.9 ± 24.4	67.2 ± 29.5	78.0 ± 47.4	68.3 ± 28.4	75.4 ± 32.7	***<0.001***

**Table 2:** Baseline characteristics of participants in non-advanced CKM stages (0-1) and advanced CKM stages (2-4b).

Characteristics	Non-advanced CKM (N = 5247)	Advanced CKM (N = 9986)	*p*
**Age (years)**	37.4 ± 13.2	56.5 ± 15.8	***<0.001***
**Age group**			
20-44	3829 (73%)	2434 (24.4%)	***<0.001***
45-64	1220 (23.3%)	4128 (41.3%)	
>=65	198 (3.8%)	3424 (34.3%)	
**Sex**			
Male	2331 (44.4%)	5077 (50.8%)	***<0.001***
Female	2916 (55.6%)	4909 (49.2%)	
**Race and ethnicity**			
Mexican American	943 (18%)	1401 (14%)	***<0.001***
Other Hispanic	577 (11%)	887 (8.9%)	
Non-Hispanic White	2136 (40.7%)	4382 (43.9%)	
Non-Hispanic Black	836 (15.9%)	2131 (21.3%)	
Other/multiracial	755 (14.4%)	1185 (11.9%)	
**CVD status**			*--*
No	5247 (100%)	8456 (84.7%)	
ASCVD	0 (0%)	1081 (10.8%)	
HF	0 (0%)	449 (4.5%)	
**Hypertension**			
Low risk	5247 (100%)	1934 (19.4%)	*--*
Hypertension	0 (0%)	8052 (80.6%)	
**Renal impairment**			
Low	5247 (100%)	7271 (72.8%)	***--***
Moderate	0 (0%)	1849 (18.5%)	
High risk	0 (0%)	523 (5.2%)	
Very-high risk	0 (0%)	343 (3.4%)	
**Diabetes status**			
No diabetes	4186 (79.8%)	4223 (42.3%)	*--*
Prediabetes	1061 (20.2%)	3792 (38%)	
Undiagnosed diabetes	0 (0%)	490 (4.9%)	
Diagnosed diabetes	0 (0%)	1481 (14.8%)	
**Cholesterol (mg/dL)**			
No	5247 (100%)	5397 (54%)	*--*
High cholesterol	0 (0%)	4589 (46%)	
**Smoking status**			
Never smoke	3275 (62.4%)	5244 (52.5%)	***<0.001***
Former smoker	847 (16.1%)	2801 (28%)	
Current smoker	1125 (21.4%)	1941 (19.4%)	
**Poverty income ratio (PIR)**			
Not poor	893 (18.9%)	1565 (17.3%)	***0.024***
Near poor	1554 (32.9%)	3138 (34.8%)	
Poor	2270 (48.1%)	4325 (47.9%)	
**Education**			
Less than high school	1070 (20.4%)	2495 (25%)	***<0.001***
High school	1112 (21.2%)	2385 (23.9%)	
Some college	1634 (31.1%)	3007 (30.1%)	
College above	1431 (27.3%)	2099 (21%)	
**BMI (kg/m²)**	27.3 ± 6.1	30.4 ± 7.3	*--*
**Phosphorus (mg/dL)**	3.7 ± 0.6	3.7 ± 0.6	*--*
**Calcium (mg/dL)**	9.4 ± 0.3	9.4 ± 0.4	
**ALP (IU/L)**	65.6 ± 20.4	74.4 ± 26.2	***<0.001***
ALP Quartile			
1^st^ Quartile	1741 (33.2%)	2077 (20.8%)	***<0.001***
2^nd^ Quartile	1406 (26.8%)	2399 (24%)	
3^rd^ Quartile	1220 (23.3%)	2588 (25.9%)	
4^th^ Quartile	880 (16.8%)	2922 (29.3%)	
**Albumin (g/dL)**	4.25 ± 0.33	4.16 ± 0.34	***<0.001***
**Vitamin D (nmol/L)**	61.7 ± 25.1	68.0 ± 30.2	***<0.001***

Regarding mortality data, S4Table presents the CKM-specific and all-cause mortality rates among participants classified into CKM stages 0–4b. Overall, mortality rates increased with advancing CKM stage, except stage 4a. Participants in stage 4b exhibited the highest mortality rates—16.6% for CKM-cause mortality and 33.3% for all-cause mortality. The most notable increase in all-cause mortality was observed between stage 2 (7.2%) and stage 3 (33.3%), which was paralleled by a similar pattern in CKM-cause mortality. Additionally, participants in the advanced CKM stages demonstrated higher mortality rates compared to those in the non-advanced CKM stages, as shown in S5Table.

### Associations of ALP quartile, calcium, and phosphorus levels with CKM, respectively

#### Survey-weighted multinomial logistic regression models.

To exclude the potential effects of confounders, survey-weighted multinomial logistic regression models were performed with the adjustment for age (years), race and ethnicity, PIR, Sex, BMI, smoking status, education, and vitamin D levels. As presented in Table 3, a higher ALP level was significantly associated with a significantly higher likelihood of being classified into stage 2 (RRR: 1.23; 95% CI: 1.11, 1.37; *p <* 0.001), stage 3 (RRR: 1.99; 95% CI: 1.48, 2.67; *p <* 0.001), stage 4a (RRR: 1.29; 95% CI: 1.14, 1.46; *p <* 0.001), and stage 4b (RRR: 1.35; 95% CI: 1.16, 1.58; *p < 0.001*) while the association of stage 1 (RRR: 1.1; 95% CI: 0.99, 1.22; *p =* 0.074) was marginally significant referring to stage 0. Moreover, a positive association was revealed between calcium levels and CKM stage 2 (RRR: 2.13; 95% CI: 1.55, 2.94; *p <* 0.001), stage 4a (RRR: 1.67; 95% CI: 1.17, 2.38; *p =* 0.006), and stage 4b (RRR: 1.81; 95% CI: 1.22, 2.68; *p =* 0.004). Similarly, a significant increase in the likelihood of being classified into stage 3 (RRR: 2.85; 95% CI: 1.77, 4.59; *p <* 0.001) and stage 4b (RRR: 1.78; 95% CI: 1.37, 2.31; *p <* 0.001) was associated with higher phosphorus level, indicating its role in late-stage CKD-driven CKM.

**Table 3:** Survey-weighted multinomial logistic regression results for associations between ALP, Calcium, Phosphorus levels and CKM stages 0-4b.

	ALP Quartile		Calcium (mg/dL)		Phosphorus (mg/dL)	
CKM Stages	RRR (95% CI)	*p*-value	RRR (95% CI)	*p*-value	RRR (95% CI)	*p*-value
Stage 0	Reference		Reference		Reference	
Stage 1	1.1 (0.99, 1.22)	*0.074*	0.99 (0. 72, 1.38)	*0.97*	0.91 (0. 77, 1.09)	*0. 296*
Stage 2	1.23 (1.11, 1.37)	***<0.001***	2.13 (1.55, 2.94)	***<0.001***	1.03 (0. 85, 1.24)	*0. 786*
Stage 3	1.99 (1.48, 2.67)	***<0.001***	1.43 (0.68, 3.02)	*0.33*	2.85 (1.77, 4.59)	***<0.001***
Stage 4a	1.29 (1.14, 1.46)	***<0.001***	1.67 (1.17, 2.38)	***0.006***	1.04 (0.82, 1.33)	*0.731*
Stage 4b	1.35 (1.16, 1.58)	***<0.001***	1.81 (1.22, 2.68)	***0.004***	1.78 (1.37, 2.31)	***<0.001***

Adjustment: Age (years), Race and ethnicity, Poverty income ratio, Sex, BMI, Smoking status, Education, and vitamin D level.

Abbreviations: RRRs, relative risk ratios; 95%CI, 95 confidence interval; CKM, Cardiovascular-Kidney-Metabolic Syndrome; BMI, body mass index.

In the survey-weighted multinomial logistic regressions of standardized three markers, the associations between per 1-SD increase of ALP and CKM stages 2-4b remained significantly positive, whereas the associations for per 1-SD increase of calcium and phosphorus were weaker (Table 4), yielding findings consistent with those from unscaled analyses.

**Table 4:** Survey-weighted multinomial logistic regression results for associations of scaled ALP, Calcium, and Phosphorus levels with CKM stages 0-4b.

	ALP (per 1-SD increase)		Calcium (per 1-SD increase)		Phosphorus (per 1-SD increase)	
CKM Stages	RRR (95% CI)	*p*-value	RRR (95% CI)	*p*-value	RRR (95% CI)	*p*-value
Stage 0	Reference		Reference		Reference	
Stage 1	1.14 (0.99, 1.32)	*0.075*	1.01 (0.89, 1.13)	*0.907*	0.94 (0. 86, 1.04)	*0.235*
Stage 2	1.29 (1.11, 1.49)	***<0.001***	1.31 (1.16, 1.47)	***<0.001***	1.00 (0.90, 1.11)	*0.989*
Stage 3	1.79 (1.47, 2.17)	***<0.001***	1.16 (0.86, 1.56)	*0.325*	1.71 (1.31, 2.22)	***<0.001***
Stage 4a	1.36 (1.16, 1.60)	***<0.001***	1.20 (1.05, 1.36)	***0.008***	1.01 (0.88, 1.15)	*0.880*
Stage 4b	1.51 (1.25, 1.82)	***<0.001***	1.24 (1.08, 1.42)	***0.003***	1.37 (1.18, 1.59)	***<0.001***

Adjustment: Age (years), Race and ethnicity, Poverty income ratio, Sex, BMI, Smoking status, Education, and vitamin D level.

Abbreviations: RRRs, relative risk ratios; 95%CI, 95 confidence interval; CKM, Cardiovascular-Kidney-Metabolic Syndrome; BMI, body mass index.

#### Survey-weighted modified Poisson regression models.

To evaluate the stability of the results from survey-weighted multinomial logistic regression models and facilitate further analysis for the associations of ALP quartiles, phosphorus, and calcium levels with CKM stages, CKM stages 0 and 1 were classified into non-advanced CKM stages and stages 2-4b were merged into advanced CKM stages. Hence, survey-weighted modified binomial regression was performed. As shown in Table 5, 2^nd^ quartile of ALP (OR: 1.42; 95% CI: 1.23, 1.64; *p* < 0.001), 3^rd^ quartile (OR: 1.76; 95% CI: 1.49, 2.07; *p* < 0.001), and 4^th^ quartile (OR: 2.72; 95% CI: 2.30, 3.22; *p* < 0.001) were positively associated with likelihood of being classified into advanced CKM stages referring to 1^st^ quartile in unadjusted Model 1. After adjusting for age (years), race and ethnicity, PIR, Sex, BMI, smoking status, education, and vitamin D levels (Model 2), the 2^nd^ ALP quartile (OR: 1.18; 95% CI: 0.98, 1.41; *p =* 0.076) was not significant but the 3^rd^ quartile (OR: 1.26; 95% CI: 1.02, 1.55; *p* = 0.033) and 4^th^ quartile (OR: 1.55; 95% CI: 1.29, 1.86; *p <* 0.001) remained significantly associated with likelihood of being classified into advanced CKM stages. Regarding calcium level, elevated calcium level was significantly associated with a higher likelihood of being classified into advanced CKM stages both in Model 1 (OR: 1.53; 95% CI: 1.37, 1.72; *p <* 0.001) and Model 2 (OR: 2.11; 95% CI: 1.76, 2.54; *p <* 0.001). There was a significantly inverse association between phosphorus and advanced CKM stages (OR: 0.90; 95% CI: 0.83, 0.98, *p =* 0.020) in Model 1, while the significance disappeared in the adjusted model (Model 2).

**Table 5:** The associations of ALP quartiles, phosphorus level, and calcium level with risk of the advanced CKM stages.

	Model 1		Model 2	
Characteristic	OR (95% CI)	*p*-value	OR (95% CI)	*p*-value
**ALP Quartile**				
1^st^ Quartile	Reference		Reference	
2^nd^ Quartile	1.42 (1.23, 1.64)	***<0.001***	1.18 (0.98, 1.41)	*0.076*
3^rd^ Quartile	1.76 (1.49, 2.07)	***<0.001***	1.26 (1.02, 1.55)	***0.033***
4^th^ Quartile	2.72 (2.30, 3.22)	***<0.001***	1.55 (1.29, 1.86)	***<0.001***
**Calcium (mg/dL)**	1.53 (1.37, 1.72)	***<0.001***	2.11 (1.76, 2.54)	***<0.001***
**Phosphorus (mg/dL)**	0.90 (0.83, 0.98)	***0.020***	1.10 (0.97, 1.24)	*0.150*

Model 1: only ALP quartiles, Calcium (mg/dL), and Phosphorus (mg/dL), without adjustment.

Model 2: Model 1, adjusted by Age (years), Race and ethnicity, Poverty income ratio, Sex, BMI, Smoking status, Education, and vitamin D level.

Abbreviations: ORs, odds ratios; 95%CI, 95% confidence interval; CKM, Cardiovascular-Kidney-Metabolic Syndrome; BMI, body mass index.

Also, in regression models standardizing the three markers, per 1-SD increase of ALP and calcium were positively associated with the advanced CKM stages in both models while association of per 1-SD increase of phosphorus with advanced CKM stages were not significant after adjustment (Table 6).

**Table 6:** Survey-weighted modified Poisson regression for associations of scaled ALP, Calcium, and Phosphorus levels, with advanced CKM stages.

	Model 1		Model 2	
Characteristic	OR (95% CI)	*p*-value	OR (95% CI)	*p*-value
ALP (per 1-SD increase)	1.54 (1.42, 1.66)	***<0.001***	1.16 (1.07, 1.25)	***<0.001***
Calcium (per 1-SD increase)	1.18 (1.13, 1.23)	***<0.001***	1.30 (1.21, 1.39)	***<0.001***
Phosphorus (per 1-SD increase)	0.94 (0.90, 0.99)	***0.013***	1.04 (0.97, 1.12)	*0.200*

Model 1: only ALP (per 1-SD increase), Calcium (per 1-SD increase), and Phosphorus (per 1-SD increase), without adjustment.

Model 2: Model 1, adjusted by Age (years), Race and ethnicity, Poverty income ratio, Sex, BMI, Smoking status, Education, and vitamin D level.

Abbreviations: ORs, odds ratios; 95%CI, 95% confidence interval; CKM, Cardiovascular-Kidney-Metabolic Syndrome; BMI, body mass index.

### Sensitivity analyses

To avoid the potential confounding effects of liver diseases on ALP, the sensitivity analyses of multinomial and binomial logistic regressions were performed by excluding the participants with liver diseases. As a result, 341 participants were excluded from the current cohort, and conclusions regarding serum ALP, calcium, and phosphorus remained consistent in both multinomial and binomial logistic regression analysis (S6–S7 Tables). Moreover, we further adjusted serum ALT and AST in the multinomial and binomial logistic regressions, revealing the same conclusions on ALP, calcium, and phosphorus in CKM stages (S8–S9 Tables). Vitamin D supplement use and phosphate binder medication use were further adjusted to minimize the confounding effects of mineral metabolism related variables. The conclusion that ALP is the most robust calcification marker across the CKM stages remained unchanged (S10–S11 Tables). These sensitivity analyses indicated the stability of the results.

Survey-weighted multinomial logistic regression results for associations of albumin-corrected calcium revealed that albumin-corrected calcium was positively associated with the likelihood of being classified into stage 2-4b, referring to stage 0 (S12 Table). Also, a positive association of albumin-corrected calcium with the likelihood of being classified into advanced CKM stages (S13 Table) and with all-cause mortality (S14 Table).

#### Subgroup analysis.

**Fig 2:**
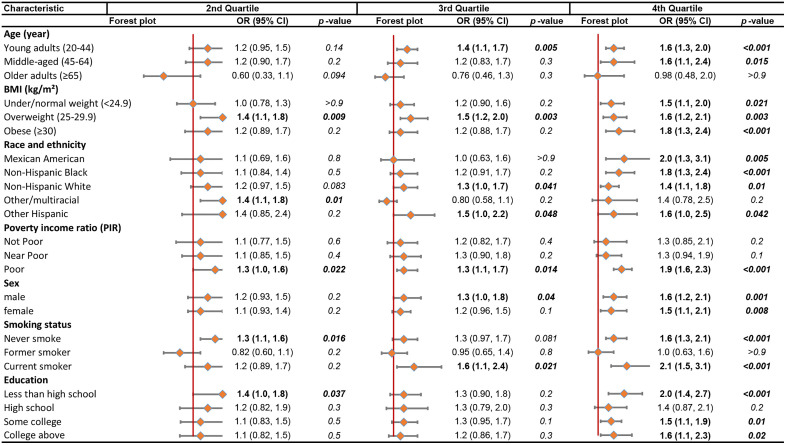
Forest plot displaying subgroup analysis for the associations between ALP quartiles and advanced CKM stages referring to 1^st^ quartile ALP. Odds ratios (ORs) and 95% CI were calculated with the survey-weighted modified Poisson regression models adjusting for age (years), race and ethnicity, PIR, sex, BMI, smoking status, education, and vitamin D level, calcium level, and phosphorus level but not the respective variable for stratification. Red vertical lines serve as references for an OR value equal to one. Abbreviations: ALP, alkaline phosphatase; CKM, Cardiovascular-Kidney-Metabolic Syndrome; OR, odds ratios; 95% CI, 95% confidence interval; PIR, poverty income ratio; BMI, body mass index.

**Fig 3:**
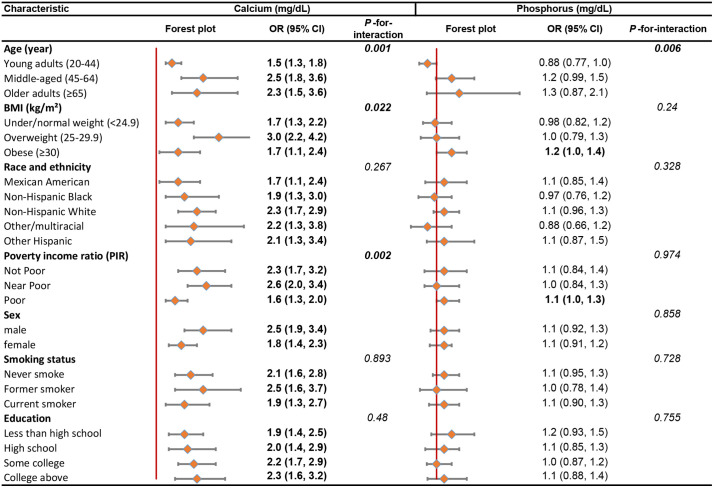
Forest plot illustrating the subgroup analysis for associations of calcium and phosphorus with risks of advanced CKM stages. Survey-weighted modified Poisson regression models were adopted to evaluate ORs and 95% CI of the associations. Red lines represent the OR values of one. The adjustments for models included: age (years), race and ethnicity, PIR, sex, BMI, smoking status, education, and vitamin D level, ALP quartiles, except the variable for subgrouping. Abbreviations: CKM, Cardiovascular-Kidney-Metabolic Syndrome; OR, odds ratios; 95% CI, 95% confidence interval; PIR, poverty income ratio; BMI, body mass index; ALP, alkaline phosphatase.

Furthermore, subgroup analyses were performed to investigate how the associations between ALP, calcium, and phosphorus levels and CKM stages vary across different sub-populations. Results of subgroup analysis for ALP quartiles and advanced CKM stages were displayed in Fig 2. Concerning the 4^th^ quartiles of ALP, participants with young (OR: 1.6; 95% CI: 1.3, 2.0; *p <* 0.001) and middle-aged (OR: 1.6; 95% CI: 1.1, 2.4; *p =* 0.015) showed a stronger association between ALP level and likelihood of being classified into advanced CKM stages, not in older adults. For the subgroups stratified by BMI, the association between ALP and advanced CKM stages remained significant, but the effect size was the highest in the obese population (OR: 1.8; 95% CI: 1.3, 2.4; *p <* 0.001). Besides, among the poor population, the association between ALP and the likelihood of advanced CKM stages was significant (OR, 1.9; 95% CI: 1.6, 2.3; *p <* 0.001) while no significance was found in the other two subgroups. Concerning the subgroups divided by sex, significant associations were found both in male (OR: 1.6; 95% CI: 1.2, 2.1; *p =* 0.001) and female (OR: 1.5; 95% CI: 1.1, 2.1; *p =* 0.008) groups. Moreover, significant associations were found in the ‘never smoke’ (OR: 1.6; 95% CI: 1.3, 2.1; *p <* 0.001) and ‘current smoker’ (OR: 2.1; 95% CI: 1.5, 3.1; *p <* 0.001) subgroups but not in the ‘former smoker’ group. Among the subgroups stratified by education, there were significant associations between ALP and advanced CKM stages in all subgroups except the ‘high school’ sub-population. However, *P*-for-interactions were calculated to investigate the interactions between ALP and the stratification variables, and no significant interactions were observed across the subgroups, indicating that associations between ALP and advanced CKM stages were relatively stable among subgroups. Moreover, Subgroup-specific survey-weighted regression models confirmed the consistently positive associations between calcium levels and advanced CKM stages across all demographic categories (Fig 3). Moreover, calcium levels were more strongly associated with advanced CKM stages in ‘middle-aged’ (OR: 2.5; 95% CI: 1.8, 3.6; *P*-for-interaction = 0.001), ‘overweight’ (OR: 3.0; 95% CI: 2.2, 4.2; *P*-for-interaction = 0.022), and the ‘near poor’ participants (OR: 2.6; 95% CI: 2.0, 3.4; *P*-for-interaction = 0.002). Besides, Fig 3 showed that phosphorus was significantly associated with advanced CKM stages only in the obese (OR: 1.2; 95% CI: 1.0, 1.4; *p =* 0.043) and poor (OR: 1.1; 95% CI: 1.0, 1.3; *p =* 0.048) subgroups. Additionally, associations between phosphorus and advanced CKM stages were not significant among different age-subgroups, though a significant interaction was discovered between age and phosphorus (*P*-for-interaction = *0.006*).

#### Linear or non-linear associations?.

**Fig 4:**
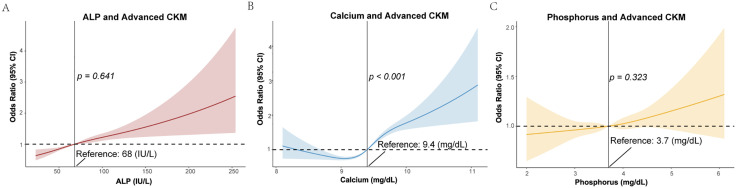
RCS analysis for the associations of ALP **(A)**, calcium **(B)**, and phosphorus **(C)** levels with risks of advanced CKM stages. OR and 95% CI were calculated and predicted with generalized linear models adjusting for age (years), race and ethnicity, PIR, sex, BMI, smoking status, education, vitamin D level, ALP level, calcium level, and phosphorus level, but not the independent variable. Colored solid lines represent their respective ORs, and the shadows are their corresponding 95% CI. Medians were set as references. The Wald test was used to test their linearity. Abbreviations: RCS, restricted cubic spline; ALP, alkaline phosphatase; CKM, Cardiovascular-Kidney-Metabolic Syndrome; OR, odds ratios; 95% CI, 95% confidence interval; PIR, poverty income ratio; BMI, body mass index; ALP, alkaline phosphatase.

A consistent tendency of RRRs was observed between ALP and CKM stages 0-4b, but not for associations of calcium and phosphorus levels with CKM stages from survey-weighted multinomial logistic regression. Hence, RCS analyses were performed together with survey-weighted binomial logistic regression, adjusting for all the confounders, to explore whether these associations were linear. Fig 4A demonstrated that the association between ALP level and the likelihood of being classified into advanced CKM stages was linear. Conversely, a significant non-linear association between calcium level and likelihood of being classified into advanced CKM stages was shown in Fig 4B and no significant adverse effect of low calcium level was observed on the odds ratio from the left side of Fig 4B. Moreover, Fig 4C exhibited a linear association between phosphorus level and the odds ratio (*p =* 0.323) and the association was not significant considering the 95%CI, which was consistent with the results in Table 5 (OR: 1.10; 95% CI: 0.97, 1.24; *p =* 0.150).

#### Associations of ALP, calcium, and phosphorus levels with CKM-cause mortality, all-cause Mortality.

**Fig 5:**
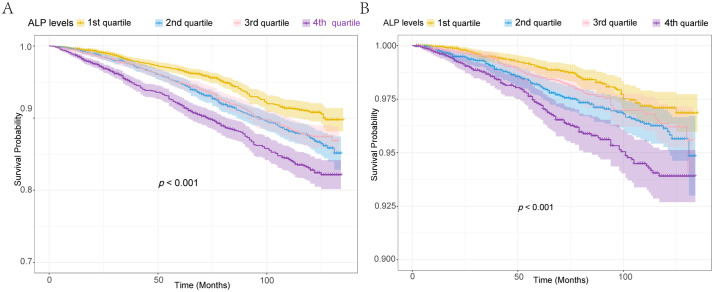
Kaplan-Meier survival curves stratified by serum ALP quartiles for the all-cause mortality (A) and CKM-cause mortality (B). Curves represent the 1st (yellow, dotted), 2nd (light blue, dashed), 3rd (pink, solid), and 4th (purple, dot-dashed) ALP quartiles, with shaded areas indicating 95% confidence intervals. Time-to-event data are shown in months; survival probabilities are displayed on a scale from 0.7–1.0 (A) and 0.9–1.0 (B). Abbreviations: ALP, alkaline phosphatase; CKM, Cardiovascular-Kidney-Metabolic Syndrome.

Additionally, survival analyses were conducted to assess whether the observed associations extended to mortality outcomes. Kaplan-Meier survival curves demonstrated significantly worse all-cause (Fig 5A) and CKM-cause (Fig 5B) mortality outcomes with higher serum ALP levels (Log-rank *p* < 0.001) over a median follow-up of 6.1 years. The cohort’s follow-up duration ranged from 1 month to 11.3 years (135 months). Furthermore, results from survey-weighted Cox proportional hazards models, adjusted for all relevant confounders, are summarized in S14 Table. Compared to the first quartile, the second quartile of ALP was significantly associated with increased CKM-cause mortality (HR: 1.56; 95% CI: 1.01–2.40; *p =* 0.043) and all-cause mortality (HR: 1.51; 95% CI: 1.14–2.01; *p =* 0.004). The fourth quartile showed even stronger associations with increased CKM-cause mortality (HR: 2.20; 95% CI: 1.47–3.28; *p <* 0.001) and all-cause mortality (HR: 2.15; 95% CI: 1.70–2.70; *p <* 0.001). The third quartile of ALP was significantly associated with all-cause mortality (HR: 1.41; 95% CI: 1.12–1.78; *p =* 0.004), but not with CKM-cause mortality (HR: 1.06; 95% CI: 0.79–1.42; p = 0.700). In contrast to ALP, lower serum calcium levels were significantly associated with higher all-cause mortality (HR: 0.74; 95% CI: 0.56–0.98; *p =* 0.038), but not with CKM-cause mortality (HR: 0.84; 95% CI: 0.58–1.22; *p =* 0.400). Elevated serum phosphorus levels were significantly associated with increased both CKM-cause mortality (HR: 2.28; 95% CI: 1.86–2.79; *p <* 0.001) and all-cause mortality (HR: 1.57; 95% CI: 1.36–1.83; *p <* 0.001).

#### Causality inference between ALP and key elements of CKM syndrome.

To explore potential causal relationships between ALP and CKD, CVD, and diabetes, separate two-sample MR analyses were conducted. As shown in Table 7, a significant positive causal effect of ALP on CVD was observed using the IVW method (OR: 1.81; 95% CI: 1.01–3.22; *p =* 0.044), although this association did not reach statistical significance in the MR-Egger analysis (OR: 2.48; 95% CI: 0.97–6.34; *p =* 0.064). Additionally, a significant causal effect of CKD on ALP levels was found using the IVW method (*β* = *7.27E-4*; SE = 3.04E-4; *p =* 0.017), whereas the association was not significant when evaluated with the MR-Egger method (*p =* 0.648). Given the absence of strong evidence of pleiotropy in the MR-Egger intercept test (S15 Table), we place greater confidence in the IVW results, which supported a potential causal relationship between ALP and CVD, as well as a potential causal effect of CKD on ALP. A reverse Mendelian randomization analysis assessing the effect of ALP on CKD found no evidence of a causal relationship. The corresponding scatter plot is shown in S3 Fig. No statistically significant causal effect of ALP on diabetes was detected using either the IVW method (OR: 1.31; 95% CI: 0.76–2.25; *p* = 0.332) or the MR-Egger method (OR: 1.71; 95% CI: 0.71–4.09; *p =* 0.237). Scatter plots illustrating the relationships between ALP and CVD, and ALP and diabetes are provided in S4 and S5 Figs, respectively. The scatter plot for CKD on ALP is shown in S6 Fig. Forest plots and leave-one-out sensitivity analyses for all MR models are presented in S7–S12 Figs, which confirmed that no single SNP had a disproportionate influence on the results. Furthermore, no significant horizontal or directional pleiotropy was detected based on Cochran’s Q and MR-Egger intercept tests, as summarized in S15 Table.

**Table 7:** Mendelian randomization evaluated the causality between ALP and CKM syndrome elements using the genetic instruments.

	No. of SNPs	IVW		MR-Egger	
		OR (95% CI)/ β (SE)	P value	OR (95% CI)/ β (SE)	P value
ALP on CVD	60	1.81 (1.01- 3.22)	***0.044***	2.48 (0.97- 6.34)	*0.064*
CKD on ALP	90	7.27E-4 (3.04E-4)	***0.017***	1.92E-4 (4.2E-4)	*0.648*
ALP on diabetes	61	1.31(0.76-2.25)	*0.332*	1.71(0.71-4.09)	*0.237*

Abbreviations: ALP, alkaline phosphatase; CVD, cardiovascular diseases; SNPs, single-nucleotide polymorphisms; IVW, inverse variance weighted; OR, odds ratio; SE, standard error; MR-Egger, Mendelian randomization-Egger.

## Discussion

The purpose of this study was to investigate and compare the associations between serum biomarkers—ALP, calcium, and phosphorus—and CKM syndrome stages, with a particular focus on the potential roles of calcification and mineral metabolism in the disease process. The current analysis revealed that elevated serum ALP and calcium levels, but not phosphorus, were significantly associated with an increased likelihood of being classified into advanced CKM stages after adjustment for all confounders. Specifically, ALP showed significant or marginally significant associations across nearly all CKM stages when compared to stage 0 and demonstrated a clear linear trend. Phosphorus was significantly associated only with stages 3 and 4b, while calcium levels were significantly associated with stages 2 and 4 following full covariate adjustment.

Recent studies have demonstrated that lower PIR, lower education levels, male sex, and older age are associated with a higher risk of advanced CKM stages [4,29], findings that align with our demographic results. Additionally, participants in CKM stages 2–4b were found to have higher vitamin D levels compared to those in stage 0, which is consistent with previous research [30], despite the frequent reports of vitamin D deficiency among individuals with kidney impairment [10]. In the present study, ALP emerged as a robust biomarker significantly and linearly associated with CKM stages, as shown by both binomial and multinomial logistic regression models. These findings are partially supported by earlier studies highlighting the relevance of ALP in the pathophysiology of both CVD and CKD [31]. Furthermore, we observed a linear association between ALP and the risk of advanced CKM stages, a result echoed by the Rafsanjan Cohort Study, which found a similar linear relationship between ALP and the risk of metabolic syndrome [31]. Although RCS analyses specific to ALP levels in CVD and CKD are limited, our findings are supported by underlying mechanistic theories. In CKD, hyperphosphatemia is common and contributes to the osteogenic transformation of vascular smooth muscle cells, a process that increases ALP release [32]. Elevated ALP, in turn, further promotes phosphate accumulation, forming a positive feedback loop between ALP and phosphate levels in CKD pathology. In CVD, in addition to vascular calcification and atherosclerosis, chronic inflammation has been shown to elevate ALP levels in neutrophils, macrophages, and certain lymphocytes [33], providing further biological plausibility for a linear association between ALP and CKM syndrome. However, serum ALP was comprised of hepatic, bone, intestinal, and placental isoenzymes, and bone and liver isoforms account for over 90% of total serum ALP activity [34]. Although our further sensitivity analysis demonstrated consistent results by excluding the participants with liver diseases and further adjusting ALT as well as AST, we could not diminish the confounding effects of subclinical hepatic dysfunction.

Regarding the relationship between serum calcium levels and CKM stages, a significant association was observed between elevated calcium levels and an increased likelihood of being classified into advanced CKM stages, both before and after full adjustment. This finding is consistent with previous studies in populations with type 2 diabetes [35] and CVD [20]. However, further stage-specific analyses revealed that calcium levels were not significantly associated with CKM stage 1 or stage 3, suggesting that calcium may be less sensitive as a marker in the context of obesity [36], kidney impairment, or subclinical CVD. This pattern may be partly explained by the bidirectional nature of calcium disturbances across CKD and CVD. Lower serum calcium levels are frequently observed in individuals with kidney impairment [37], while elevated calcium levels have been linked to increased CVD risk [38]. Additionally, dialysis treatment can influence serum calcium concentrations [39]. Therefore, the coexistence of CKD and CVD may attenuate or mask calcium fluctuations, limiting its predictive value for specific CKM stages [40]. Moreover, RCS analysis revealed a non-linear association between calcium levels and advanced CKM stages after full adjustment, consistent with prior reports in patients with advanced CKD [41]. Dysregulation of calcium homeostasis can occur throughout the progression of CKM syndrome. For instance, lower calcium levels may be present in early CKD, potentially reflecting impaired activation of vitamin D [42]. In contrast, the mechanisms underlying hypercalcemia in the late stages of CKM remain poorly understood and warrant further investigation.

Hypoalbuminemia, a common disturbance in hospitalized patients, has been reported to mask the increased concentration of calcium among the critically ill patients [27], which was also observed among the current cohort, where CKM stage 0 exhibited the highest serum albumin concentrations. Also, CKD-related ‘masked’ hypocalcemia might be partially explained by this phenomenon, in which ionized calcium (active form) concentration can be normal or even higher but the total calcium level is lower due to the reduced albumin-bound calcium [40]. Hence, our biologically inconsistent results between positive associations of calcium level with CKM stages and its inverse association with all-cause mortality since end-stage participants tended to have masked hypocalcemia. Our subsequent results using albumin-corrected calcium [43] instead of total calcium (S12–S14 Tables) exhibited positive associations of albumin-corrected calcium both with CKM stage 2-4b and all-cause mortality, documenting the rationality of the explanation.

Consistent with a previous randomized controlled trial [44], our findings indicate that, after adjusting for confounders, elevated serum phosphorus levels are primarily associated with CKM stages characterized by very high-risk renal impairment (stages 3 and 4b). In contrast, phosphorus levels showed no significant association with CVD accompanied by low-level renal impairment (stage 4a) or with metabolic diseases (stages 1 and 2). This suggests that serum phosphorus is a more specific predictor for CKD than for CVD or metabolic conditions. However, there is currently insufficient strong evidence linking serum phosphorus levels with metabolic syndrome to further support this hypothesis. To put together, our current data suggested that serum phosphorus levels served as a late-stage CKD marker and CKD-driven CKM rather than a general CKM biomarker. Notably, the COX analysis results of phosphorus indicated that phosphorus may be more relevant to mortality risk than CKM stage classification.

Moreover, the current subgroup analysis revealed a stronger association between ALP levels and the likelihood of being classified into advanced CKM stages in young and middle-aged adults compared to older populations, consistent with findings previously reported in peritoneal dialysis patients [45]. This may be explained by higher ALP levels in older participants (mean = 71.65 IU/L) with non-advanced CKM stages compared to middle-aged (mean = 69.45 IU/L) and young adults (mean = 64.03 IU/L), while ALP levels among those with advanced CKM stages remained relatively stable across age groups. Interestingly, after adjusting for confounders, the association between ALP and advanced CKM stages was not significant in former smokers (OR: 1.0; 95% CI: 0.63–1.6; *p > 0.9*), though no clear explanation has been identified for this observation. Notably, compared to calcium and phosphorus, ALP showed more consistent associations with CKM stage, as also observed in peritoneal dialysis patients [46]. Regarding calcium levels, subgroup analysis by age showed a stronger association between serum calcium and advanced CKM stages in middle-aged individuals compared to younger adults. This may be due to accelerated vascular calcification with advancing age [47], which is often accompanied by endothelial dysfunction and chronic inflammation [48]. Additionally, the study found that the overweight subgroup exhibited a stronger association between calcium and advanced CKM stages than the under/normal weight or obese subgroups (*P*-for-interaction = *0.022*). This is intriguing, as previous research reported significantly lower calcium levels in overweight/obese individuals compared to those with normal weight [49], but did not differentiate between overweight and obese groups.

As for the causal relationships between ALP and CVD, CKD, and diabetes, the current study demonstrated a potential positive effect of ALP on CVD, aligning with previous research that identified associations between liver enzyme-related SNPs and coronary heart disease as well as ischemic stroke [50]. Although a positive trend was observed between ALP and diabetes, this association did not reach statistical significance. Due to limited prior studies on this topic, further inference on the relationship between ALP and diabetes remains uncertain. Given that the MR analysis relied on European-ancestry summary statistics, while NHANES included participants from diverse racial and ethnic backgrounds, the causal inferences from MR analysis here may not fully generalize to NHANES population and explain the conclusions in NHANES data.

The current study also suffers from some limitations. Firstly, residual confounding still exists, like parathyroid hormone levels or genetic information, though we adjusted for the confounders we know. Besides, definitions of CKM stages from AHA [2] were adapted for the NHANES data due to the limited available variables, such as the subclinical CVD, resulting in a less accurate classification of stage 3. Furthermore, we could not differentiate the bone ALP and liver ALP since they are both abundant in serum. Other residual confounders related to mineral metabolism, such as parathyroid hormone, fibroblast growth factor-23, were not included in the adjustment due to absence of data. Hence, further research is needed to extend the analysis and confirm the role of ALP in CKM syndrome.

In summary, considering the associations with the likelihood of all CKM stages 0-4b, serum ALP seemed to be more robust than serum calcium and phosphorus since it remained significant or marginally significant across all the stages and linearly associated with the likelihood of being classified into advanced CKM stages. Also, serum ALP levels were strongly and positively associated with both CKM-cause and all-cause mortality, with higher quartiles corresponding to increased mortality risk during follow-up. This highlights ALP as a significant predictor of mortality outcomes in CKM syndrome.

## Conclusions

Our study reveals significant associations between serum ALP levels and CKM syndrome stages. These findings suggest that ALP may serve as a marker for CKM syndrome. While calcium showed stage-specific associations and phosphorus was less informative, ALP demonstrated consistent linear relationships across CKM stages. However, while mineralization processes have been implicated in CKM, further research is needed to elucidate the precise mechanisms through which ALP influences vascular and tissue calcification within this context.

## Supporting information

S1 Fig Schematic Diagram of the Two-Sample Mendelian Randomization (MR) Analysis Framework Investigating the Causal Relationship Between Serum Alkaline Phosphatase and Cardiovascular Disease/Diabetes. (PNG)

S2 Fig Schematic Diagram of the Two-Sample Mendelian Randomization (MR) Analysis Investigating the Causal Effect of Chronic Kidney Disease on Serum Alkaline Phosphatase Levels. (PNG)

S3 Fig Scatter plot of Mendelian randomization analysis for the causal effect of serum alkaline phosphatase (ALP) on chronic kidney disease (CKD). (TIFF)

S4 Fig Scatter Plot of Mendelian Randomization Analysis for the Causal Effect of Serum Alkaline Phosphatase (ALP) on cardiovascular disease (CVD). (TIFF)

S5 Fig Scatter plot of Mendelian randomization analysis for the causal effect of serum alkaline phosphatase (ALP) on type 2 diabetes (T2D). (TIFF)

S6 Fig Scatter plot of Mendelian randomization analysis for the causal effect of chronic kidney disease (CKD) on serum alkaline phosphatase (ALP). (TIFF)

S7 Fig Forest plot of SNP Mendelian randomization analyses assessing the causal effect of serum alkaline phosphatase (ALP) on cardiovascular disease (CVD). (TIFF)

S8 Fig Forest plot of single‑SNP Mendelian randomization analyses assessing the causal effect of serum alkaline phosphatase (ALP) on type 2 diabetes (T2DM). (TIFF)

S9 Fig Forest plot of Mendelian randomization analysis for the causal effect of chronic kidney disease (CKD) on serum alkaline phosphatase (ALP). (TIFF)

S10 Fig Forest plot of leave-one-out sensitivity analysis for the effect of serum alkaline phosphatase (ALP) on cardiovascular disease (CVD). (TIFF)

S11 Fig Forest plot of leave-one-out sensitivity analysis for the effect of serum alkaline phosphatase (ALP) on type 2 diabetes (T2DM). (TIFF)

S12 Fig Forest plot of leave-one-out sensitivity analysis for the effect of chronic kidney disease (CKD) on serum alkaline phosphatase (ALP). (TIFF)

S1 Table The definitions of CKM-related disease or risk factors. (DOCX)

S2 Table Characteristics of GWAS datasets enrolled in the MR study. (DOCX)

S3 Table SNPs of ALP used to construct the instrumental variable for the MR analysis. (DOCX)

S4 Table Mortality rates of participants with CKM stages(0-4b). (DOCX)

S5 Table Mortality rates of participants in non-advanced and advanced CKM stages. (DOCX)

S6 Table Survey-weighted multinomial logistic regression results for associations between ALP, Calcium, Phosphorus levels, and CKM stages 0-4b after excluding participants with liver diseases. (DOCX)

S7 Table The associations of ALP quartiles, phosphorus level, and calcium level with the likelihood of being classified into the advanced CKM stages after excluding the participants with liver diseases. (DOCX)

S8 Table Survey-weighted multinomial logistic regression results for associations between ALP, Calcium, Phosphorus levels, and CKM stages 0-4b, with the further adjustment of ALT, AST. (DOCX)

S9 Table The associations of ALP quartiles, phosphorus level, and calcium level with the likelihood of being classified into the advanced CKM stages, with the further adjustment of ALT, AST. (DOCX)

S10 Table Survey-weighted multinomial logistic regression results for associations of ALP Quartile, Calcium, and Phosphorus levels with CKM stages 0-4b with the further adjustment of ALT, AST, vitamin D supplement intake, and phosphate binder use. (DOCX)

S11 Table The associations of ALP quartiles, albumin-corrected calcium level, phosphorus level, and the likelihood of being classified into the advanced CKM stages with the further adjustment of ALT, AST, vitamin D supplement intake, and phosphate binder use. (DOCX)

S12 Table Survey-weighted multinomial logistic regression results for associations between ALP, Albumin-corrected calcium, Phosphorus levels, and CKM stages 0-4b, with the further adjustment of ALT, AST. (DOCX)

S13 Table The associations of ALP quartiles, albumin-corrected calcium level, phosphorus level, and the likelihood of being classified into the advanced CKM stages. (DOCX)

S14 Table ALP quartiles, calcium, albumin-corrected calcium, and phosphorus on CKM-cause and all-cause mortality. (DOCX)

S15 Table Heterogeneity and pleiotropy test for Mendelian analysis. (DOCX)
