# Supplementary figures and images for: Stage-specific associations of mineralization markers with CKM syndrome: Nationwide survey and genetic evidence for Alkaline phosphatase’s unique clinical role
Source: PLoS One. 2026 Jun 18;21(6):e0351946. doi: 10.1371/journal.pone.0351946 (PMC13278675; doi:10.1371/journal.pone.0351946)

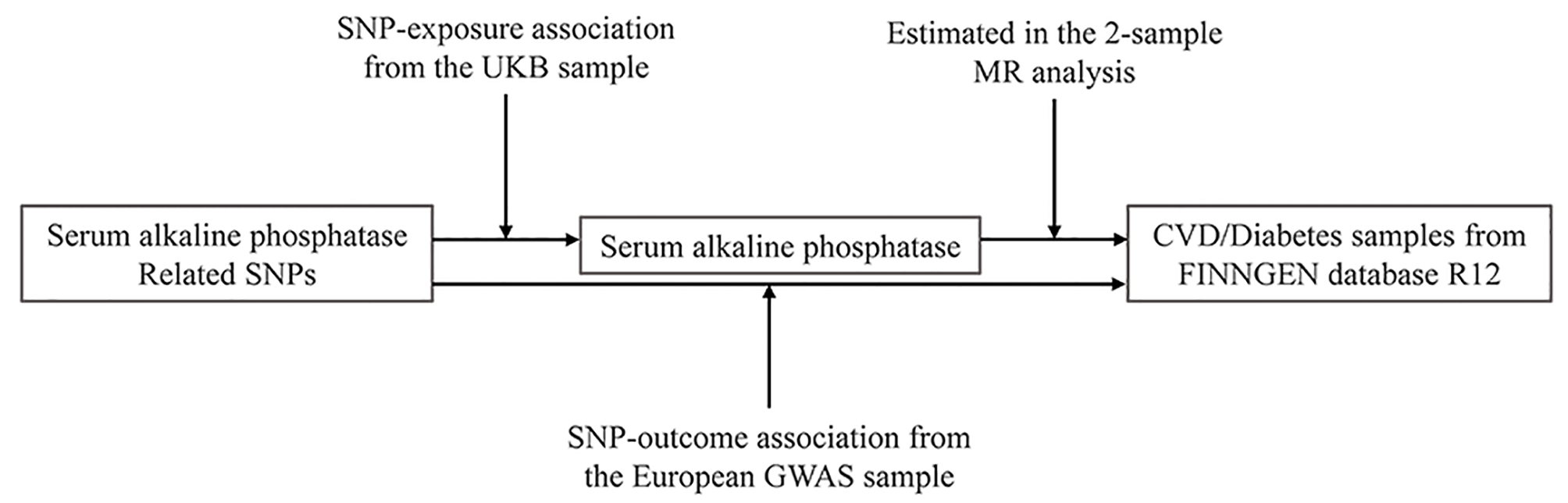

Supplement: S1 Fig — (PNG) [file pone.0351946.s001.png]

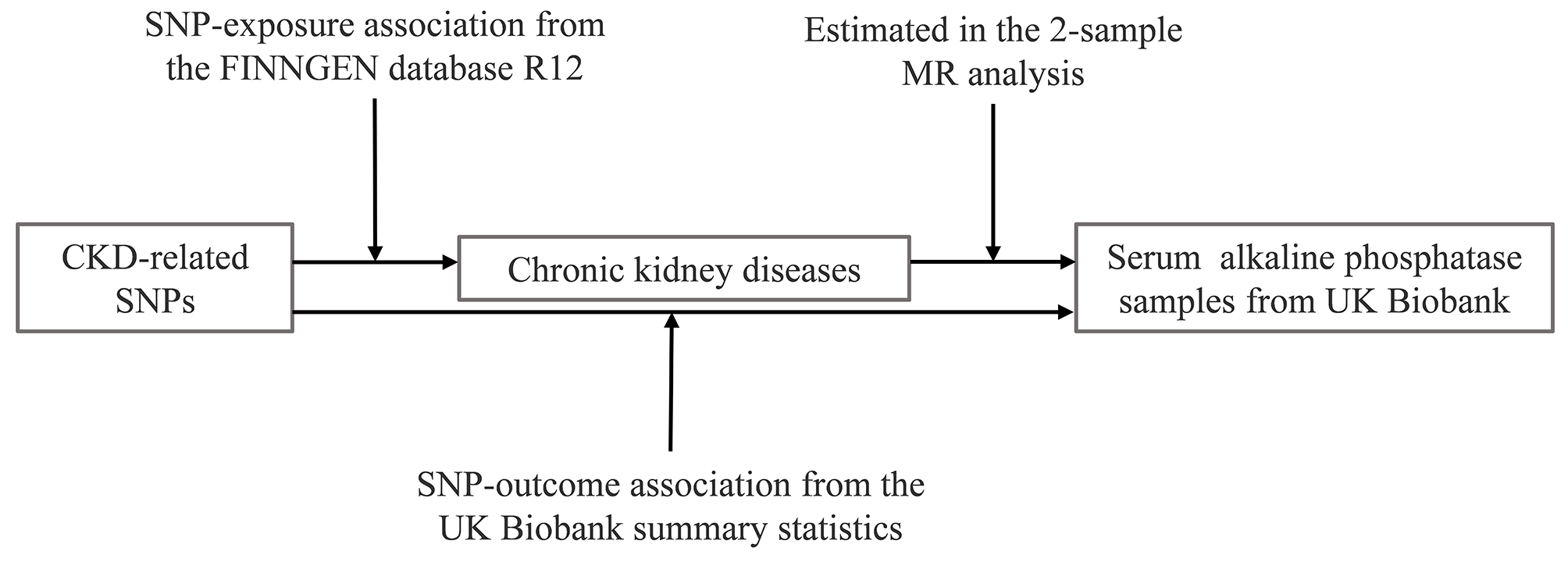

Supplement: S2 Fig — (PNG) [file pone.0351946.s002.png]

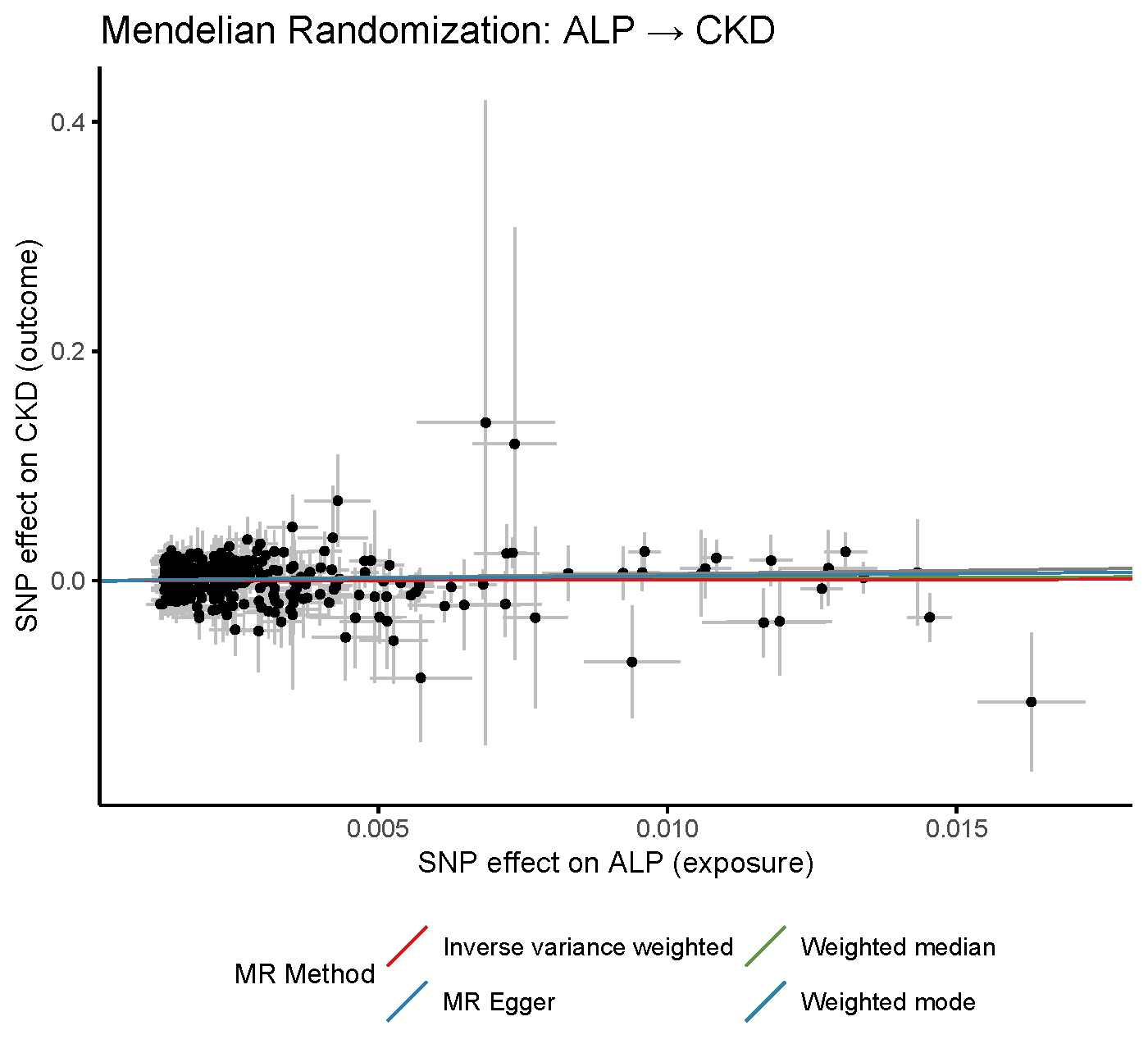

Supplement: S3 Fig — (TIFF) [file pone.0351946.s003.tiff]

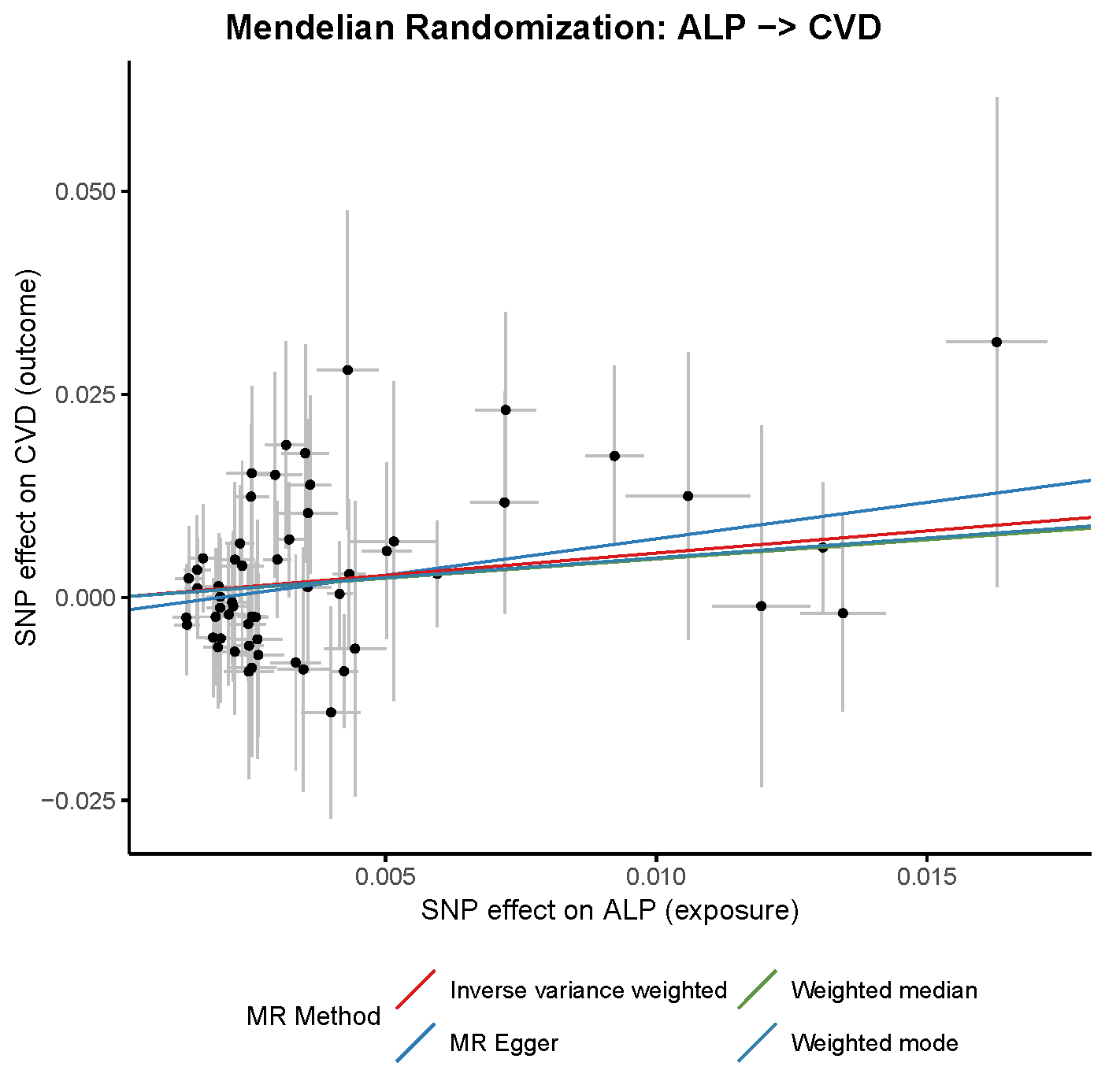

Supplement: S4 Fig — (TIFF) [file pone.0351946.s004.tiff]

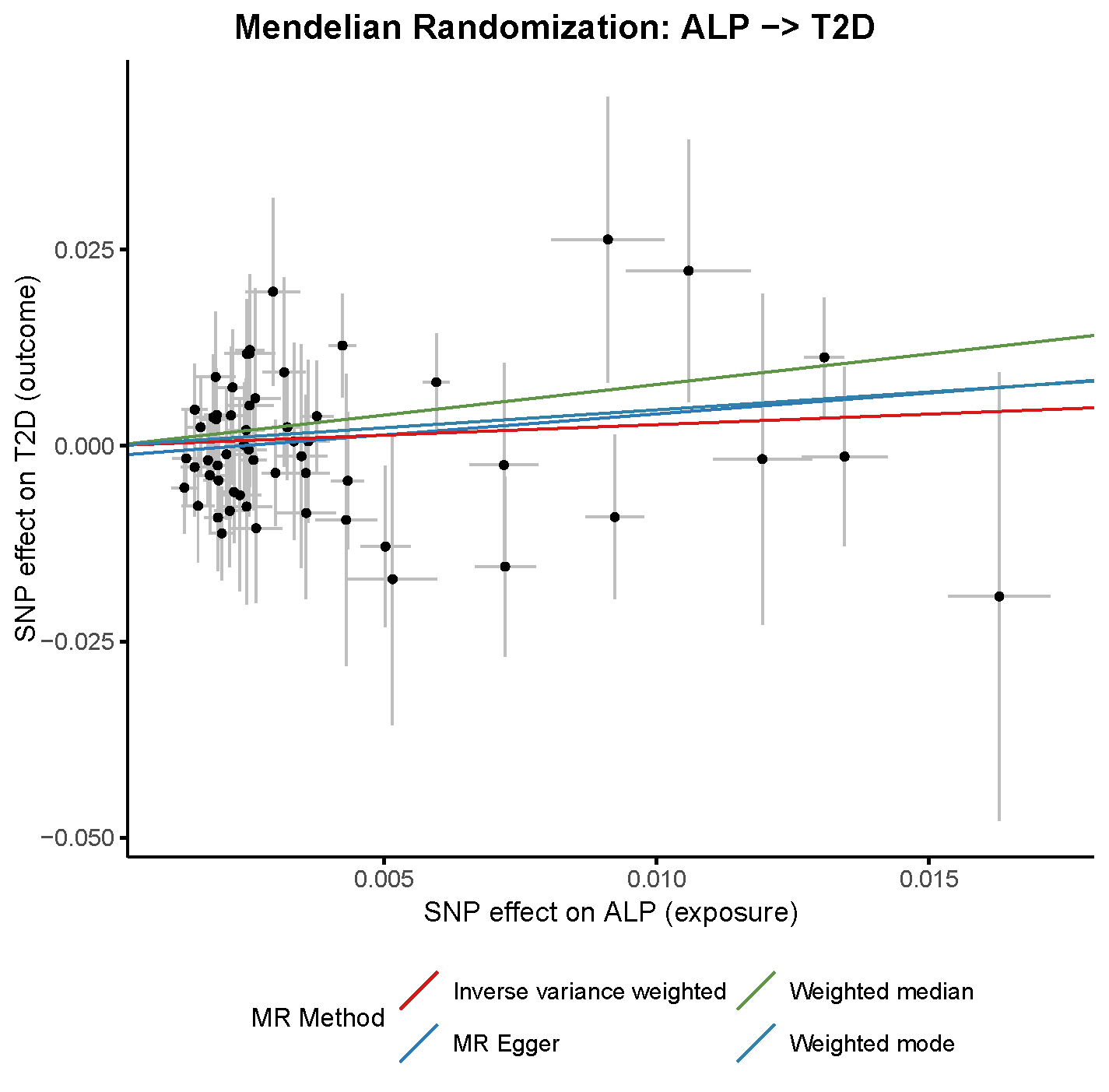

Supplement: S5 Fig — (TIFF) [file pone.0351946.s005.tiff]

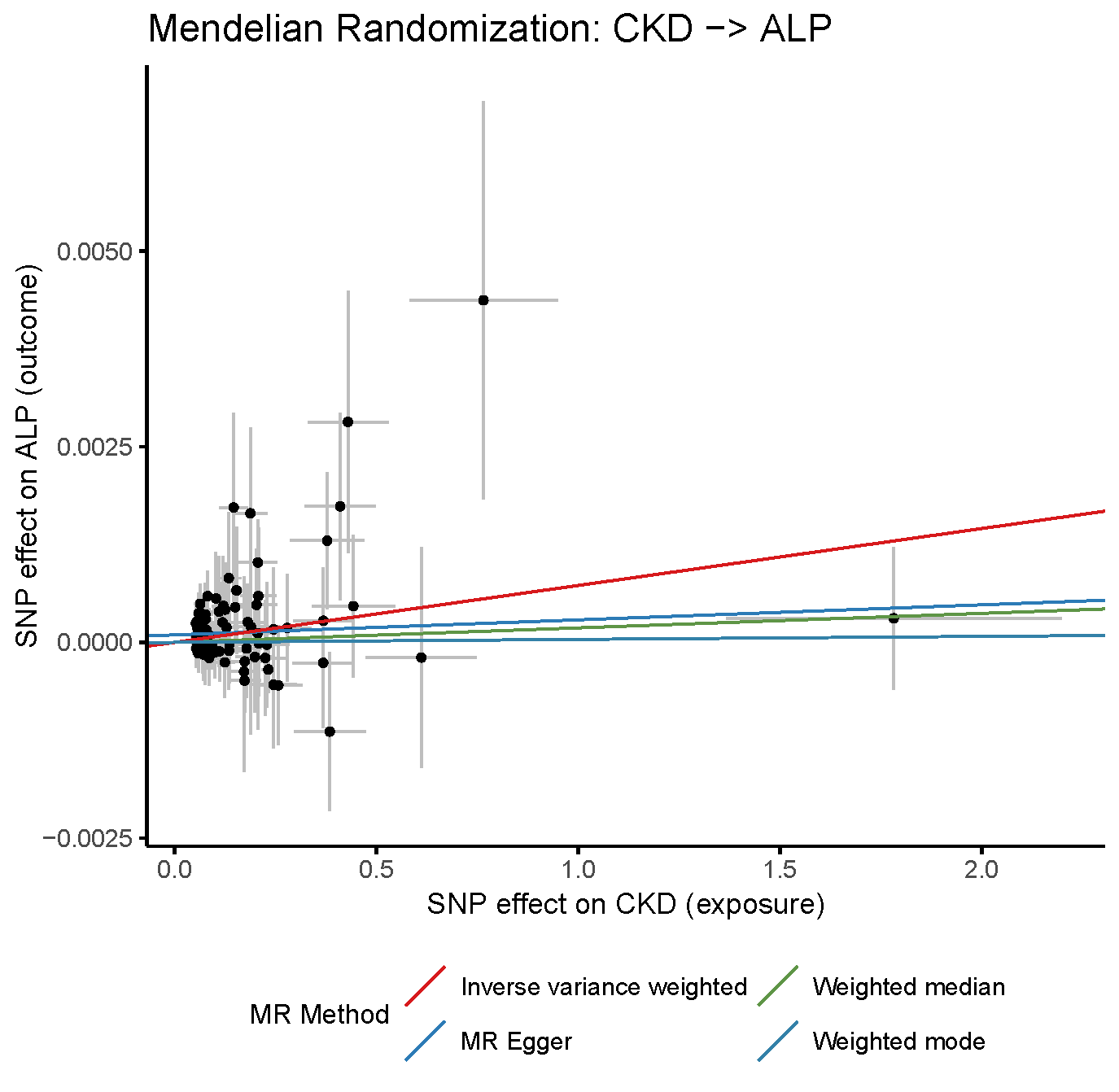

Supplement: S6 Fig — (TIFF) [file pone.0351946.s006.tiff]

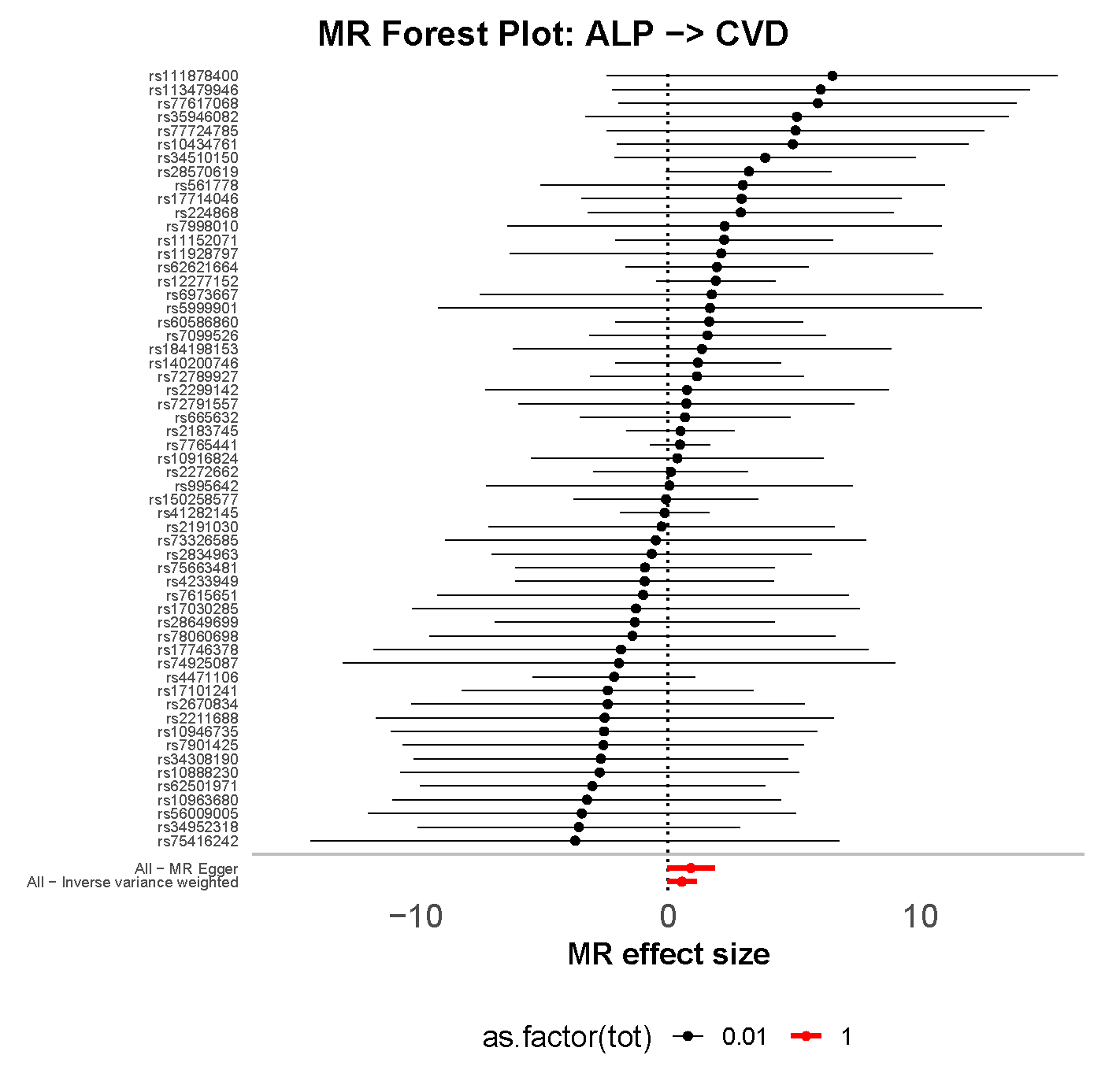

Supplement: S7 Fig — (TIFF) [file pone.0351946.s007.tiff]

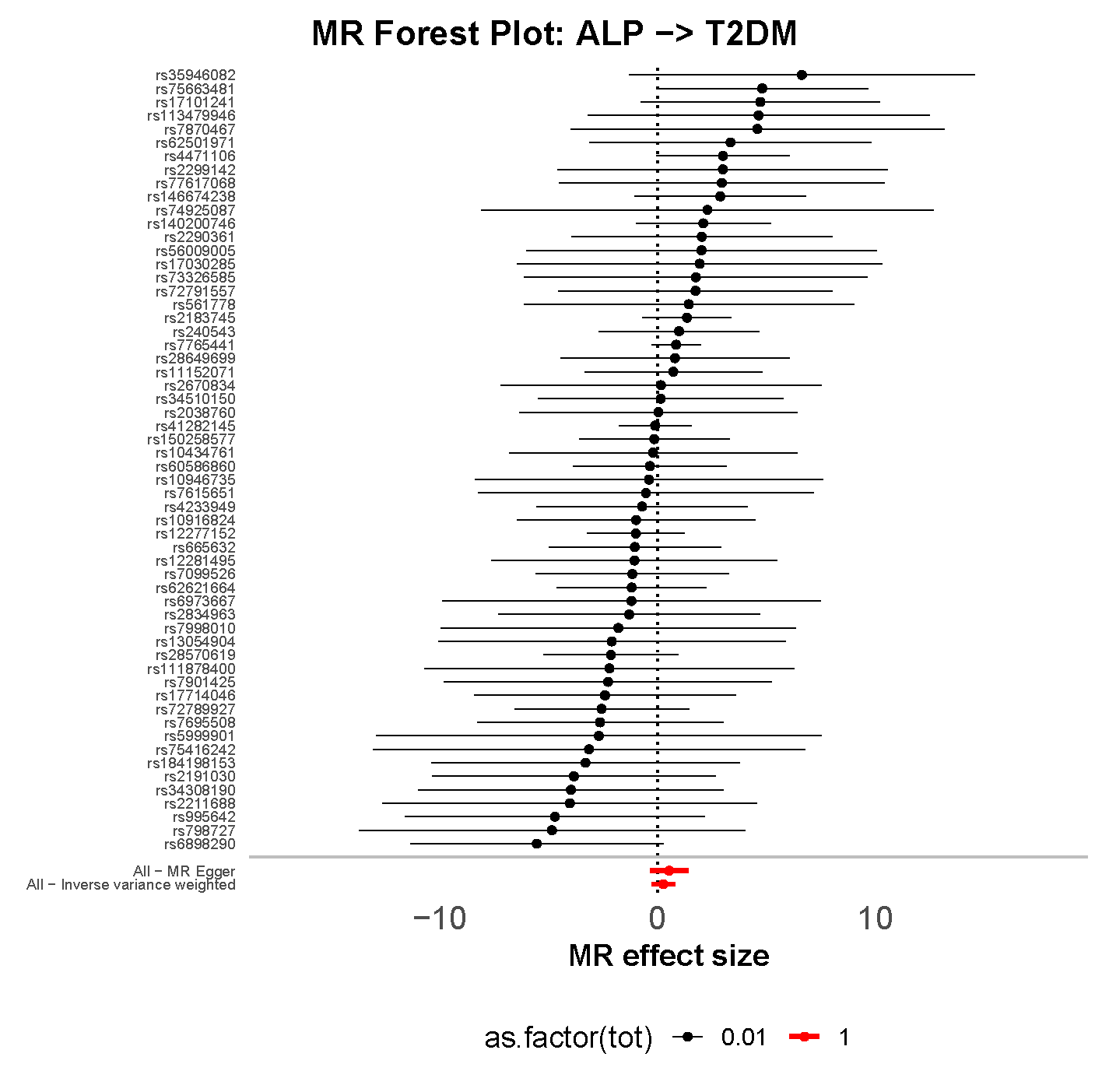

Supplement: S8 Fig — (TIFF) [file pone.0351946.s008.tiff]

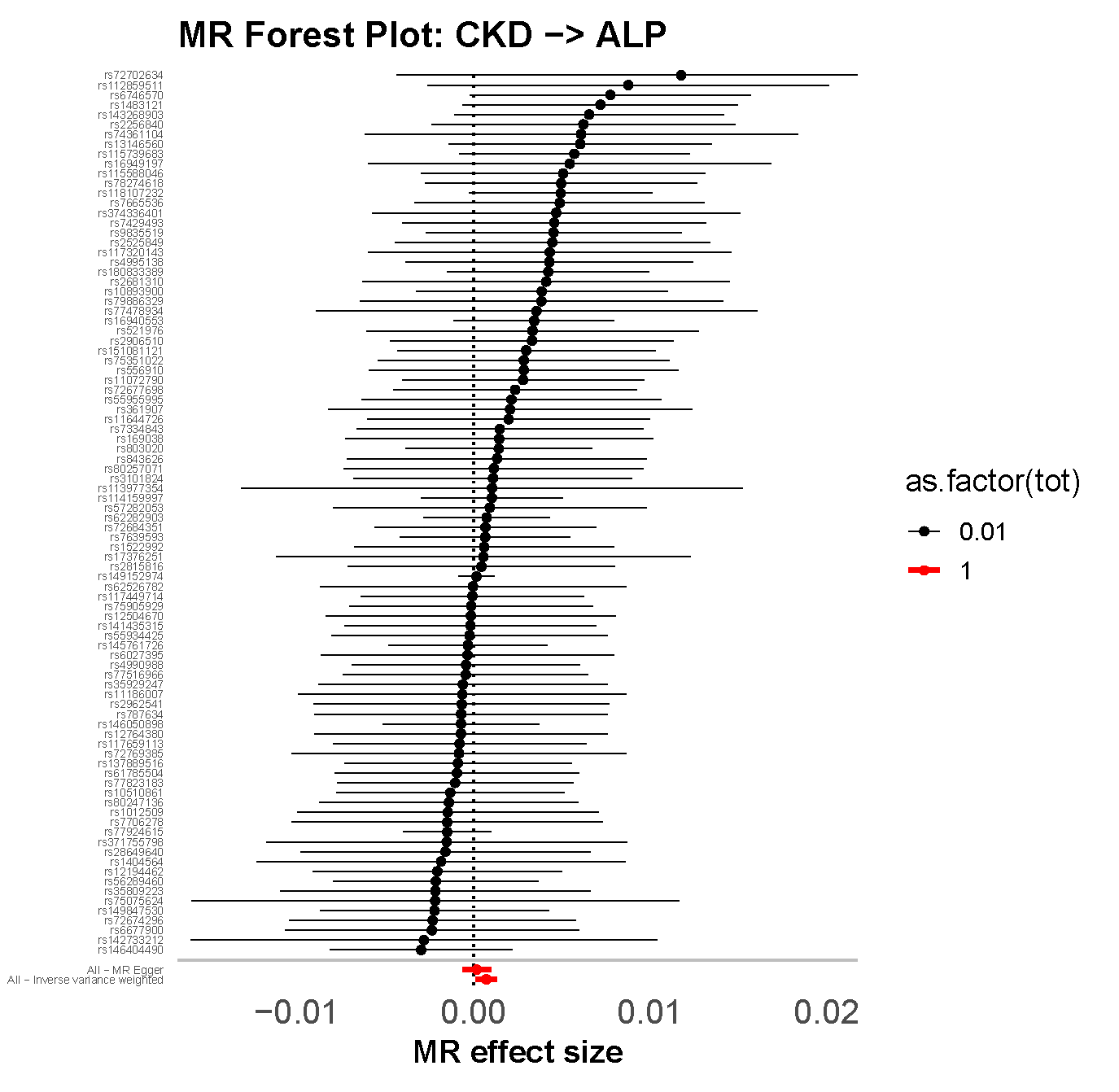

Supplement: S9 Fig — (TIFF) [file pone.0351946.s009.tiff]

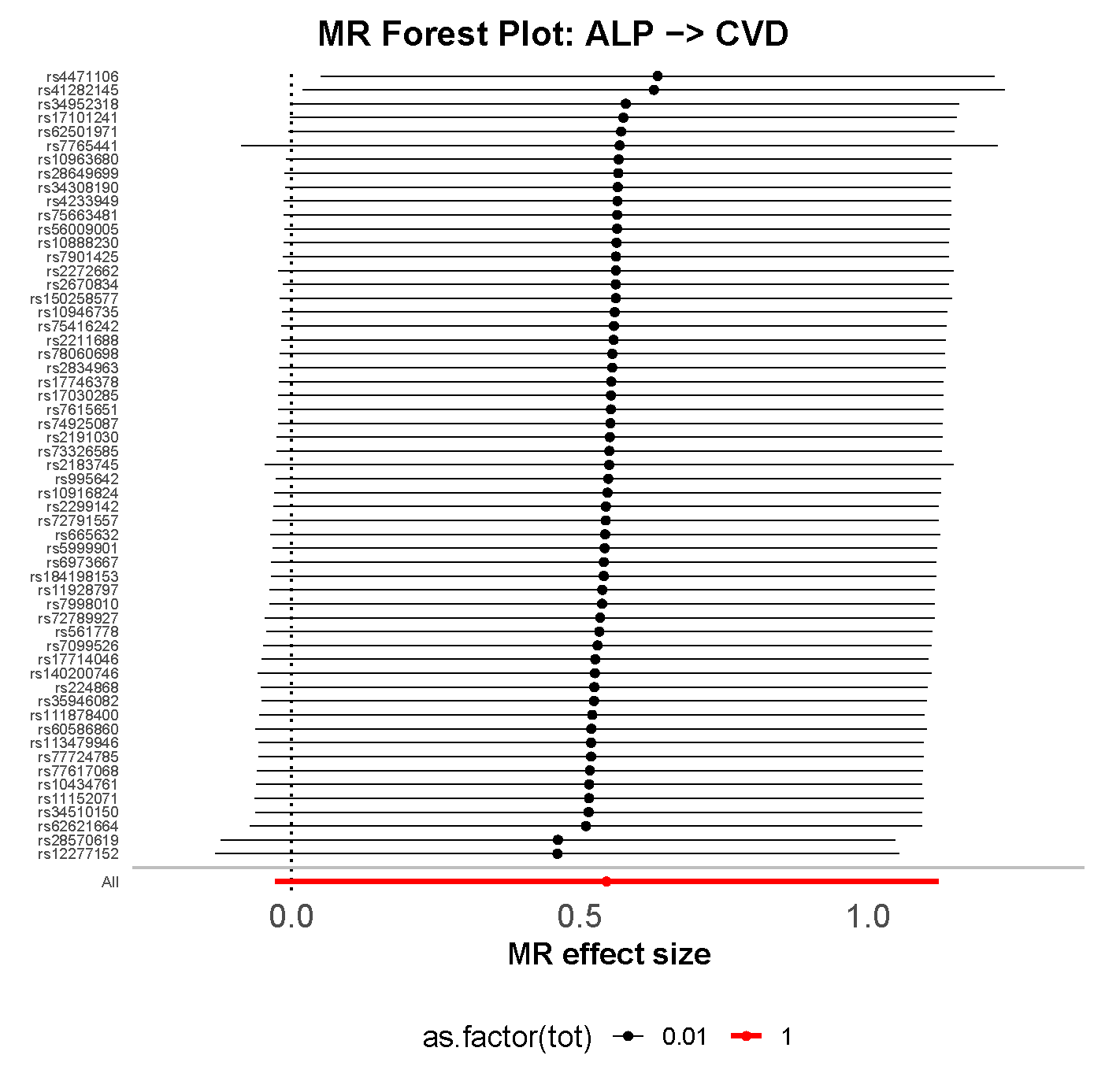

Supplement: S10 Fig — (TIFF) [file pone.0351946.s010.tiff]

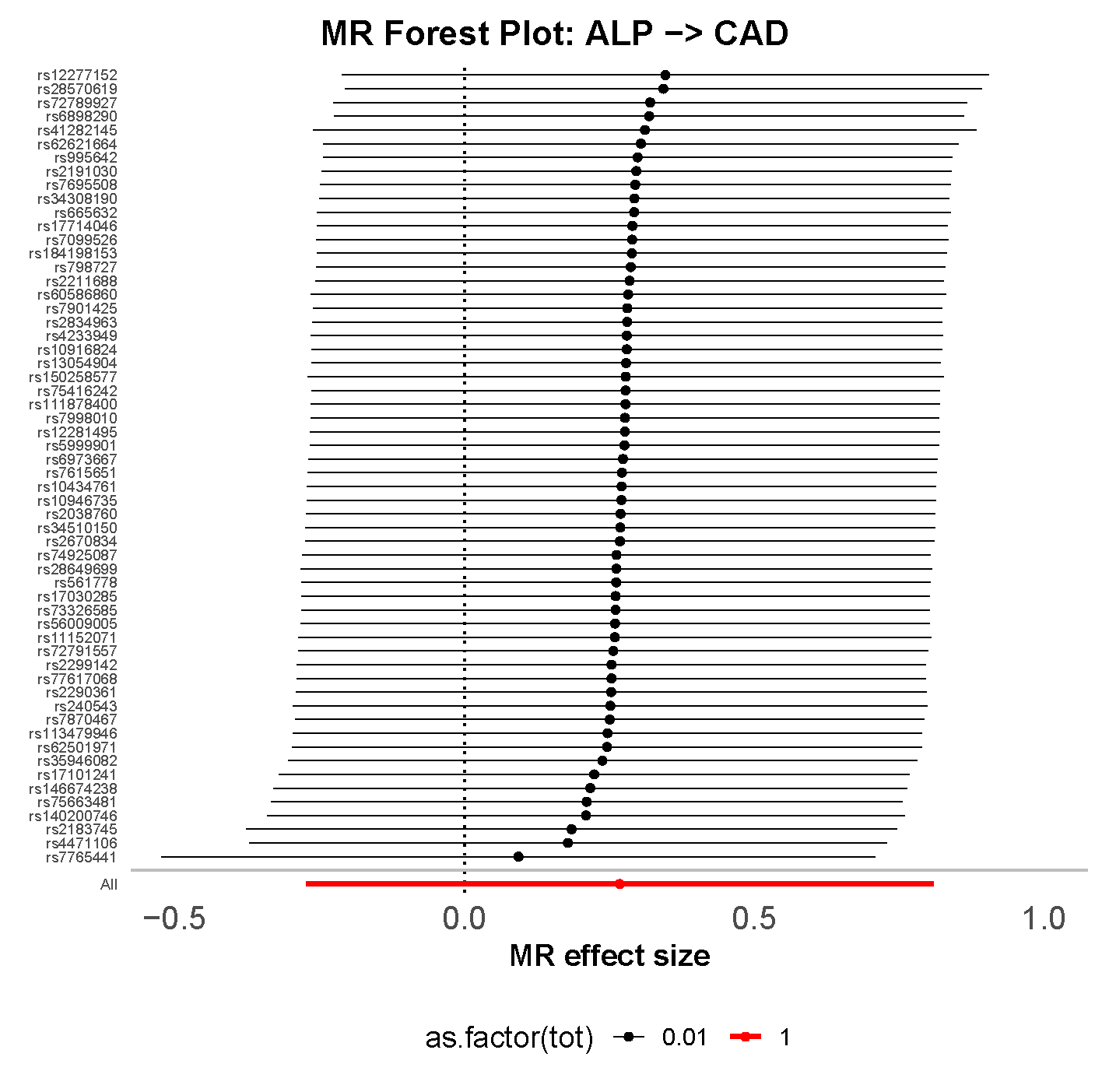

Supplement: S11 Fig — (TIFF) [file pone.0351946.s011.tiff]

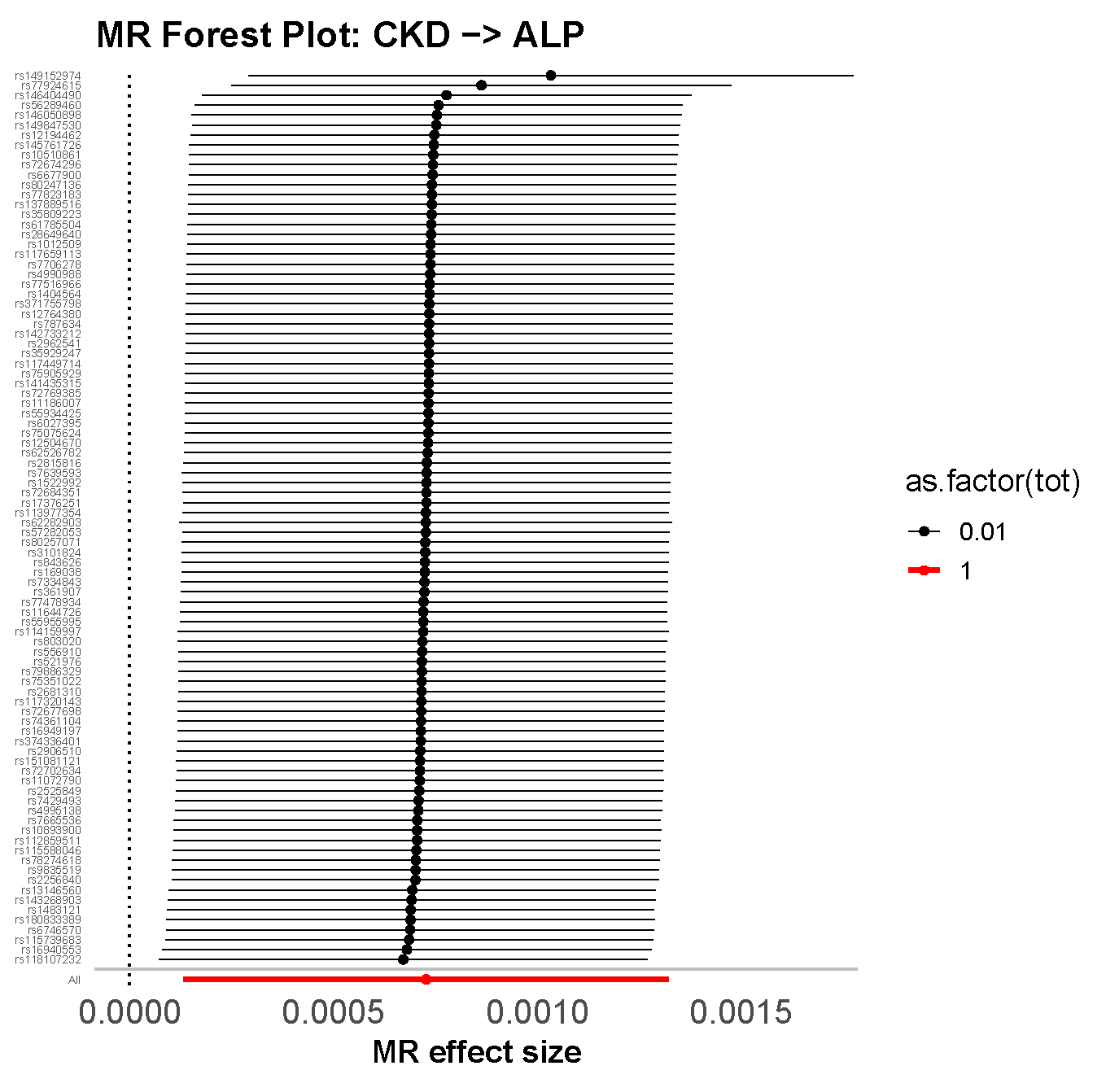

Supplement: S12 Fig — (TIFF) [file pone.0351946.s012.tiff]
