# Supplementary material for: Stage-specific associations of mineralization markers with CKM syndrome: Nationwide survey and genetic evidence for Alkaline phosphatase’s unique clinical role
Source: PLoS One. 2026 Jun 18;21(6):e0351946. doi: 10.1371/journal.pone.0351946 (PMC13278675; doi:10.1371/journal.pone.0351946)
Supplement: S1 Table — (DOCX) [file pone.0351946.s013.docx]

**Table S1. The definitions of CKM-related disease or risk factors.**

| Condition | Definition | NHANES variables |
| --- | --- | --- |
| Cardiovascular disease | Self-reported congestive heart failure, coronary heart disease, myocardial infarction, or stroke. | MCQ160B, MCQ160C, MCQ160E, MCQ160F |
| Renal impairment | Moderate or higher CKD risk; | URDACT, LBXSCR, RIAGENDR, RIDRETH1, RIDAGEYR |
|  | Moderate risk: eGFR [1] 45-59 and ACR <30, or eGFR ≥60 and ACR 30-300; |  |
|  | High risk: eGFR 30-44 and ACR <30, eGFR 45-59 and ACR 30-300, or eGFR ≥60 and ACR >300; |  |
|  | Very high risk: eGFR <30 and ACR <30, eGFR <45 and ACR 30-300, or eGFR < 60 and ACR >300. |  |
| Metabolic disease | Diabetes: HbA1c ≥ 6.5% or self-reported diabetes. | DIQ010, LBXGH, DIQ070, DIQ175W, DIQ050, DIQ060U, DIQ040, DID060 |
| Hypertension | Mean systolic blood pressure ≥ 130 mmHg, or mean diastolic blood pressure ≥ 80 mmHg, or self-reported use of anti-hypertension medication. | BPXSY1-BPXSY4, BPXDI1-BPXDI4, BPQ050A, BPQ030, BPQ040A |
| Prediabetes | Self-reported prediabetes. | DIQ160 |
| Obesity | BMI>25 or waist > 102 cm for women or waist < 88 cm for men. | BMXBMI, BMXWAIST |
| Hyper-Cholesterol | Total cholesterol ≥239 mg/dL OR self-reported using medicine to lower cholesterol levels. | LBXTC, LBXTR, BPQ100D |

1. Inker LA, Eneanya ND, Coresh J, Tighiouart H, Wang D, Sang Y, Crews DC, Doria A, Estrella MM, Froissart M *et al*: **New Creatinine- and Cystatin C-Based Equations to Estimate GFR without Race**. *N Engl J Med* 2021, **385**(19):1737-1749.

Abbreviations: CKM, Cardiovascular-Kidney-Metabolic Syndrome; CKD, chronic kidney disease; eGFR, estimated glomerular filtration rate; ACR, Urine albumin to creatinine ratio; BMI, body mass index.

**Table S2. SNPs of ALP used to construct the instrumental variable for the MR analysis.**

|  | SNP | Exclude/Keep | Exclusion reason | β | *P* value |
| --- | --- | --- | --- | --- | --- |
| 1 | rs112905931 | Exclude | HDL | 0.001527 | 4.60E-11 |
| 2 | rs76703526 | Exclude | BMI | -0.00162 | 5.60E-11 |
| 3 | rs6693447 | Exclude | eGFR | 0.002136 | 3.10E-21 |
| 4 | rs537643 | Keep | / | 0.004942 | 6.30E-41 |
| 5 | rs10916824 | Keep | / | 0.003561 | 8.70E-15 |
| 6 | rs213047 | Exclude | Education | -0.00514 | 3.60E-112 |
| 7 | rs78156905 | Exclude | eGFR | -0.01285 | 1.00E-200 |
| 8 | rs1256335 | Exclude | CKD | -0.0096 | 1.00E-200 |
| 9 | rs10159452 | Exclude | Educational attainment | -0.00956 | 1.00E-200 |
| 10 | rs2291826 | Exclude | Metabolic syndrome | 0.002681 | 1.40E-25 |
| 11 | rs2746056 | Exclude | HDL cholesterol levels | 0.00237 | 2.50E-24 |
| 12 | rs348142 | Exclude | Blood pressure | -0.00141 | 9.40E-10 |
| 13 | rs11210918 | Exclude | Smoking initiation | -0.00238 | 1.10E-26 |
| 14 | rs1883783 | Exclude | Triglycerides | 0.001432 | 1.50E-10 |
| 15 | rs28649699 | Keep | / | 0.002464 | 3.30E-14 |
| 16 | rs4655584 | Exclude | BMI | -0.00271 | 4.10E-30 |
| 17 | rs12120849 | Keep | / | -0.00264 | 4.60E-11 |
| 18 | rs34517439 | Exclude | Whole body fat | 0.002738 | 7.40E-15 |
| 19 | rs66649041 | Exclude | LDL cholesterol levels | -0.00155 | 6.90E-10 |
| 20 | rs12133576 | Exclude | Testosterone levels | -0.0019 | 1.80E-15 |
| 21 | rs1762486 | Exclude | Testosterone levels | -0.00151 | 1.30E-10 |
| 22 | rs180029 | Exclude | LDL cholesterol levels | -0.00294 | 2.90E-24 |
| 23 | rs2622360 | Exclude | Educational attainment | 0.001358 | 1.70E-08 |
| 24 | rs4526605 | Exclude | Type 2 diabetes | -0.00216 | 1.30E-12 |
| 25 | rs12730935 | Exclude | CAD | -0.00378 | 2.10E-61 |
| 26 | rs539657009 | Exclude | HDL cholesterol levels | 0.002989 | 1.30E-17 |
| 27 | rs12938 | Exclude | Type 2 diabetes | -0.00157 | 1.30E-09 |
| 28 | rs1556975 | Exclude | BMI | 0.002204 | 1.70E-14 |
| 29 | rs1609829 | Exclude | eGFR | 0.002155 | 2.00E-18 |
| 30 | rs78444298 | Exclude | eGFR | -0.00939 | 5.90E-31 |
| 31 | rs56276813 | Exclude | Diabetes | 0.002019 | 1.70E-14 |
| 32 | rs10158305 | Exclude | Cardiovascular disease | 0.002154 | 4.00E-21 |
| 33 | rs10864088 | Exclude | Testosterone levels | -0.00265 | 2.90E-27 |
| 34 | rs2642438 | Exclude | LDL cholesterol levels | 0.003266 | 2.50E-41 |
| 35 | rs607407 | Exclude | Educational attainment | 0.002309 | 3.00E-21 |
| 36 | rs2587534 | Exclude | Total cholesterol levels | 0.001752 | 5.60E-16 |
| 37 | rs56188865 | Exclude | Coronary artery disease | -0.00255 | 1.30E-27 |
| 38 | rs6542680 | Exclude | Coronary artery disease | -0.00292 | 2.50E-23 |
| 39 | rs571468 | Exclude | Triglycerides | 0.002062 | 1.30E-19 |
| 40 | rs2339928 | Exclude | Smoking | -0.00136 | 1.20E-08 |
| 41 | rs1260326 | Exclude | Triglyceride levels | -0.00615 | 1.20E-155 |
| 42 | rs2244911 | Exclude | Type 2 diabetes | -0.00267 | 6.00E-13 |
| 43 | rs1043463 | Exclude | Testosterone levels | 0.001712 | 3.00E-12 |
| 44 | rs34848455 | Exclude | Blood urea nitrogen | -0.00164 | 1.80E-12 |
| 45 | rs75416242 | Keep | / | 0.002474 | 8.60E-09 |
| 46 | rs58978223 | Exclude | Educational attainment | -0.00155 | 2.60E-12 |
| 47 | rs4233949 | Keep | / | -0.0026 | 1.20E-28 |
| 48 | rs72809469 | Exclude | Cholesterol levels | -0.0022 | 8.50E-10 |
| 49 | rs2422287 | Exclude | Systolic blood pressure | 0.001563 | 2.80E-11 |
| 50 | rs2706762 | Exclude | Educational attainment | -0.00254 | 8.70E-16 |
| 51 | rs2919872 | Exclude | LDL levels | -0.0025 | 1.40E-26 |
| 52 | rs12470621 | Exclude | LDL cholesterol levels | 0.001425 | 3.30E-10 |
| 53 | rs2244097 | Exclude | LDL cholesterol levels | 0.001951 | 3.20E-12 |
| 54 | rs6741180 | Exclude | Total cholesterol levels | 0.00266 | 4.20E-30 |
| 55 | rs4848324 | Exclude | Educational attainment | -0.00152 | 3.60E-11 |
| 56 | rs995642 | Keep | / | -0.00195 | 9.80E-17 |
| 57 | rs2460383 | Exclude | LDL cholesterol levels | -0.00356 | 6.90E-38 |
| 58 | rs10201242 | Exclude | BMI | -0.00358 | 3.30E-16 |
| 59 | rs853774 | Exclude | Diabetes | 0.002405 | 5.80E-25 |
| 60 | rs2161037 | Exclude | Total cholesterol levels | -0.00467 | 2.70E-92 |
| 61 | rs13006699 | Exclude | Triglycerides | 0.001925 | 4.00E-14 |
| 62 | rs4414647 | Exclude | Smoking cessation | 0.001985 | 2.10E-16 |
| 63 | rs13013390 | Exclude | Obesity-related traits | 0.002343 | 4.60E-22 |
| 64 | rs2041081 | Exclude | Educational attainment | 0.002999 | 3.60E-36 |
| 65 | rs111540166 | Exclude | Coronary artery disease | -0.00164 | 2.20E-09 |
| 66 | rs1048013 | Exclude | BMI | 0.002674 | 6.70E-30 |
| 67 | rs10932194 | Exclude | BMI | 0.001492 | 4.30E-10 |
| 68 | rs10716631 | Exclude | Systolic blood pressure | -0.00208 | 2.50E-18 |
| 69 | rs838717 | Exclude | Type 2 diabetes | -0.00151 | 1.00E-11 |
| 70 | rs62193162 | Exclude | HDL cholesterol | -0.00194 | 8.60E-16 |
| 71 | rs7615651 | Keep | / | 0.002104 | 2.70E-11 |
| 72 | rs9851487 | Exclude | Waist-to-hip ratio | 0.001473 | 1.70E-10 |
| 73 | rs147667955 | Exclude | Cholesterol | -0.01449 | 4.50E-29 |
| 74 | rs73027292 | Exclude | BMI | 0.001885 | 2.20E-08 |
| 75 | rs11928797 | Keep | / | 0.002221 | 1.50E-10 |
| 76 | rs2191030 | Keep | / | -0.00217 | 5.60E-17 |
| 77 | rs990211 | Exclude | Body fat | 0.002485 | 5.00E-18 |
| 78 | rs11130349 | Exclude | BMI | -0.00182 | 4.60E-11 |
| 79 | rs9843214 | Exclude | Cholesterol levels | -0.00241 | 4.20E-26 |
| 80 | rs2682383 | Exclude | Diastolic blood pressure | -0.00213 | 8.10E-19 |
| 81 | rs676556 | Exclude | calcium | 0.001638 | 2.70E-08 |
| 82 | rs10934243 | Keep | / | -0.00151 | 3.70E-10 |
| 83 | rs189174 | Exclude | Total cholesterol levels | 0.002157 | 2.30E-19 |
| 84 | rs1574115 | Exclude | Vascular diseases | 0.001753 | 1.30E-10 |
| 85 | rs62292471 | Exclude | LDL cholesterol | 0.003204 | 1.10E-17 |
| 86 | rs687339 | Exclude | Hypertension | 0.003772 | 1.00E-44 |
| 87 | rs251487 | Exclude | BMI | -0.00162 | 2.70E-12 |
| 88 | rs171448 | Exclude | Triglycerides | -0.00161 | 7.30E-11 |
| 89 | rs74925087 | Keep | / | -0.00263 | 1.00E-09 |
| 90 | rs3172469 | Exclude | Blood pressure | -0.00147 | 2.60E-08 |
| 91 | rs61588443 | Exclude | Diastolic blood pressure | 0.00152 | 3.30E-09 |
| 92 | rs111632154 | Exclude | Systolic blood pressure | 0.00355 | 8.40E-12 |
| 93 | rs798727 | Keep | Hip circumference | 0.001584 | 4.70E-08 |
| 94 | rs17746378 | Keep | / | 0.001321 | 5.30E-09 |
| 95 | rs13149040 | Exclude | Educational attainment | 0.00133 | 1.30E-09 |
| 96 | rs16895705 | Exclude | Systolic blood pressure | 0.001515 | 1.40E-08 |
| 97 | rs77617068 | Keep | / | 0.003164 | 1.40E-16 |
| 98 | rs11726170 | Exclude | Systolic blood pressure | 0.001961 | 2.80E-08 |
| 99 | rs146674238 | keep | / | -0.00911 | 2.10E-19 |
| 100 | rs34707604 | Exclude | Triglyceride levels | -0.00401 | 1.10E-49 |
| 101 | rs11931182 | Exclude | Obesity | -0.00165 | 2.80E-12 |
| 102 | rs1441916 | Exclude | Testosterone levels | 0.001628 | 7.90E-10 |
| 103 | rs6822283 | Exclude | Educational attainment | -0.00187 | 2.90E-11 |
| 104 | rs35946082 | Keep | / | 0.00296 | 9.40E-11 |
| 105 | rs2728101 | Exclude | Serum uric acid levels | -0.00213 | 1.90E-11 |
| 106 | rs6822348 | Exclude | Triglycerides | -0.00482 | 8.60E-86 |
| 107 | rs1154449 | Exclude | Hypertension | 0.002035 | 8.20E-13 |
| 108 | rs11733749 | Exclude | eGFR | -0.00168 | 2.90E-12 |
| 109 | rs17429745 | Exclude | Educational attainment | -0.00238 | 2.50E-23 |
| 110 | rs7695508 | Keep | / | -0.00224 | 4.30E-22 |
| 111 | rs13109814 | Exclude | Type 2 diabetes | 0.001881 | 8.00E-15 |
| 112 | rs1021956 | Exclude | Total testosterone levels | 0.004176 | 1.80E-43 |
| 113 | rs4696659 | Exclude | LDL cholesterol levels | -0.00247 | 1.00E-24 |
| 114 | rs77945361 | Exclude | HDL cholesterol | -0.00248 | 9.50E-14 |
| 115 | rs1428967 | Exclude | Total cholesterol levels | 0.003822 | 9.60E-44 |
| 116 | rs292171 | Exclude | BMI | -0.00153 | 5.10E-10 |
| 117 | rs1499279 | Exclude | Cholesterol levels | -0.00526 | 3.10E-21 |
| 118 | rs4074793 | Exclude | Type 2 diabetes | 0.003209 | 8.00E-14 |
| 119 | rs12655885 | Exclude | eGFR | -0.0018 | 1.90E-14 |
| 120 | rs252761 | Exclude | BMI | 0.001633 | 2.50E-13 |
| 121 | rs224868 | Keep | / | 0.002311 | 3.20E-19 |
| 122 | rs10037512 | Exclude | Educational attainment | 0.002329 | 1.50E-23 |
| 123 | rs10434761 | Keep | / | 0.002515 | 2.20E-14 |
| 124 | rs72793726 | Exclude | Cholesteryl ester levels | -0.00168 | 2.80E-08 |
| 125 | rs72801474 | Exclude | HDL cholesterol levels | -0.00354 | 1.10E-18 |
| 126 | rs6876106 | Exclude | ventricular volume | -0.00135 | 1.70E-08 |
| 127 | rs6898290 | Keep | / | 0.00202 | 1.50E-16 |
| 128 | rs2278222 | Exclude | BMI | -0.00192 | 5.60E-11 |
| 129 | rs17714046 | Keep | / | 0.003568 | 1.30E-10 |
| 130 | rs2038760 | Keep | / | -0.00243 | 2.50E-15 |
| 131 | rs7751058 | Exclude | Type 2 diabetes | -0.00189 | 1.10E-18 |
| 132 | rs75768740 | Exclude | Not associated with ALP | -0.00686 | 6.60E-09 |
| 133 | rs3846829 | Exclude | eGFR | -0.00806 | 1.00E-164 |
| 134 | rs62401885 | Exclude | BMI | 0.014531 | 1.00E-200 |
| 135 | rs7765441 | Keep | / | -0.01308 | 1.00E-200 |
| 136 | rs62621664 | Keep | / | -0.01629 | 3.10E-70 |
| 137 | rs10946735 | Keep | / | -0.00348 | 3.40E-13 |
| 138 | rs73400425 | Exclude | Coronary artery disease | 0.007715 | 6.00E-46 |
| 139 | rs4713840 | Exclude | Triglyceride levels | 0.00296 | 4.50E-22 |
| 140 | rs13202921 | Exclude | BMI | 0.001442 | 1.10E-09 |
| 141 | rs3997520 | Exclude | Stroke | 0.001719 | 6.40E-15 |
| 142 | rs7450517 | Exclude | BMI | -0.00217 | 1.50E-17 |
| 143 | rs9361645 | Exclude | BMI | -0.00132 | 4.90E-08 |
| 144 | rs77795767 | Exclude | BMI | -0.00163 | 2.40E-10 |
| 145 | rs3822856 | Exclude | LDL cholesterol levels | 0.002244 | 1.20E-20 |
| 146 | rs577721086 | Exclude | BMI | -0.00337 | 4.20E-12 |
| 147 | rs13437326 | Exclude | Creatinine | 0.001331 | 1.70E-09 |
| 148 | rs453639 | Exclude | Phosphate levels | 0.00172 | 2.70E-13 |
| 149 | rs4896621 | keep | / | 0.001355 | 6.10E-09 |
| 150 | rs6910479 | keep | / | 0.001704 | 8.90E-13 |
| 151 | rs12208357 | Exclude | Triglycerides | 0.003534 | 6.60E-16 |
| 152 | rs11753995 | Exclude | Lipoprotein (a) levels | 0.001688 | 1.10E-08 |
| 153 | rs78894484 | Exclude | LDL levels | 0.001726 | 2.00E-11 |
| 154 | rs6460898 | Exclude | Triglycerides | 0.001676 | 6.20E-14 |
| 155 | rs12531256 | Exclude | eGFR | -0.00137 | 4.60E-09 |
| 156 | rs7802331 | Exclude | HDL cholesterol levels | 0.001163 | 4.40E-08 |
| 157 | rs13226357 | Exclude | Type 2 diabetes | 0.00188 | 6.90E-14 |
| 158 | rs6973667 | keep | / | -0.00137 | 1.90E-08 |
| 159 | rs2299142 | keep | / | 0.001523 | 1.40E-11 |
| 160 | rs11540050 | Exclude | Triglyceride levels | -0.00371 | 1.90E-12 |
| 161 | rs13245728 | Exclude | eGFR | -0.00149 | 7.40E-10 |
| 162 | rs13225347 | Exclude | Triglyceride levels | -0.00305 | 1.70E-33 |
| 163 | rs6467960 | Exclude | eGFR | 0.002251 | 1.50E-08 |
| 164 | rs10464592 | Exclude | Diastolic blood pressure | 0.002253 | 7.90E-23 |
| 165 | rs7806524 | Exclude | Testosterone levels | -0.00166 | 2.50E-13 |
| 166 | rs75999022 | Exclude | HDL cholesterol | -0.00284 | 1.10E-25 |
| 167 | rs140292667 | Exclude | No strong associations with ALP | 0.004939 | 1.40E-08 |
| 168 | rs62621812 | exclude | LDL cholesterol level | -0.01278 | 1.30E-52 |
| 169 | rs4728142 | Exclude | eGFR | 0.001713 | 6.50E-14 |
| 170 | rs2290361 | keep | / | -0.00193 | 2.10E-17 |
| 171 | rs273965 | Exclude | Blood pressure | 0.001489 | 2.90E-11 |
| 172 | rs115946508 | Exclude | No strong associations with ALP | -0.0031 | 5.40E-16 |
| 173 | rs73169671 | Exclude | Sex | -0.00224 | 2.50E-10 |
| 174 | rs12334083 | keep | / | 0.001367 | 1.90E-09 |
| 175 | rs2936502 | Exclude | LDL cholesterol | 0.001732 | 3.70E-14 |
| 176 | rs983309 | Exclude | HDL cholesterol levels | -0.01267 | 1.00E-200 |
| 177 | rs11250024 | Exclude | Diastolic blood pressure | 0.003189 | 3.30E-31 |
| 178 | rs2645443 | Exclude | Triglycerides levels | 0.00238 | 3.40E-19 |
| 179 | rs1907955 | Exclude | Triglyceride levels | 0.001329 | 3.60E-08 |
| 180 | rs17091891 | Exclude | Cholesterol levels in IDL | -0.00252 | 9.00E-13 |
| 181 | rs62501971 | keep | Smoking initiation | -0.00222 | 2.30E-15 |
| 182 | rs17435276 | Exclude | BMI | -0.00146 | 4.80E-08 |
| 183 | rs2977986 | Exclude | HDL cholesterol levels | -0.00272 | 2.10E-18 |
| 184 | rs62509311 | Exclude | Total cholesterol levels | -0.00183 | 7.50E-11 |
| 185 | rs2436854 | Exclude | HDL cholesterol levels | -0.00193 | 9.30E-14 |
| 186 | rs4876611 | Exclude | Total cholesterol levels | 0.001397 | 1.70E-08 |
| 187 | rs6469788 | Exclude | Educational attainment | 0.005434 | 3.50E-128 |
| 188 | rs16900538 | Exclude | LDL cholesterol levels | -0.0025 | 8.00E-11 |
| 189 | rs2954022 | Exclude | Triglyceride levels | -0.00681 | 1.00E-200 |
| 190 | rs13267392 | Exclude | Type 2 diabetes | -0.00151 | 1.40E-08 |
| 191 | rs2272662 | Keep | / | -0.00415 | 6.00E-70 |
| 192 | rs7870467 | Keep | / | 0.001907 | 4.50E-08 |
| 193 | rs4471106 | Keep | / | 0.004234 | 2.50E-64 |
| 194 | rs56009005 | Keep | / | -0.00253 | 5.50E-09 |
| 195 | rs10963680 | Keep | / | 0.001903 | 3.20E-11 |
| 196 | rs556587401 | Exclude | Total cholesterol levels | 0.001904 | 4.20E-08 |
| 197 | rs61237993 | Exclude | Type 2 diabetes | -0.00271 | 3.80E-16 |
| 198 | rs2812365 | Exclude | Diastolic blood pressure | 0.001313 | 2.70E-09 |
| 199 | rs28713678 | Exclude | Type 2 diabetes | -0.00171 | 7.70E-13 |
| 200 | rs816680 | Exclude | Hip circumference | -0.00164 | 5.50E-12 |
| 201 | rs2183745 | Keep | / | -0.00596 | 1.50E-133 |
| 202 | rs41282145 | Keep | / | 0.013449 | 5.20E-73 |
| 203 | rs140200746 | Keep | / | -0.01059 | 2.60E-20 |
| 204 | rs7860208 | Exclude | Systolic blood pressure | 0.001269 | 2.20E-08 |
| 205 | rs7855852 | Exclude | Pulse pressure | -0.00145 | 9.80E-12 |
| 206 | rs150258577 | Keep | / | -0.01194 | 2.00E-38 |
| 207 | rs34510150 | Keep | / | -0.00361 | 2.80E-21 |
| 208 | rs11793170 | Exclude | Total cholesterol levels | -0.01339 | 1.00E-200 |
| 209 | rs7025162 | Exclude | Total cholesterol levels | -0.01084 | 1.00E-200 |
| 210 | rs71483206 | Exclude | ADAMTS13 protein levels | -0.01212 | 8.00E-32 |
| 211 | rs11523307 | Exclude | LDL cholesterol levels | -0.01179 | 1.00E-200 |
| 212 | rs76669111 | Exclude | Triglyceride levels | 0.00291 | 1.60E-21 |
| 213 | rs4748509 | Exclude | Sudden cardiac arrest | -0.00138 | 2.70E-09 |
| 214 | rs72789927 | Keep | / | -0.00502 | 1.40E-27 |
| 215 | rs10900229 | Exclude | Cholesterol levels | -0.00233 | 5.30E-20 |
| 216 | rs77724785 | Keep | / | -0.00352 | 1.30E-16 |
| 217 | rs538108105 | Keep | / | 0.005399 | 2.10E-11 |
| 218 | rs184198153 | Keep | / | 0.005154 | 4.20E-09 |
| 219 | rs2842280 | Exclude | No strong associations with ALP | -0.00573 | 8.60E-11 |
| 220 | rs79093169 | Exclude | Educational attainment | -0.00874 | 7.90E-17 |
| 221 | rs3858122 | Exclude | Free cholesterol levels | 0.007007 | 1.00E-200 |
| 222 | rs35496892 | Exclude | Systolic blood pressure | -0.00137 | 1.50E-09 |
| 223 | rs7099526 | Keep | / | 0.003003 | 6.90E-34 |
| 224 | rs10824742 | Exclude | BMI | 0.001557 | 4.60E-11 |
| 225 | rs7916812 | Exclude | Diastolic blood pressure | -0.00145 | 8.20E-09 |
| 226 | rs7087141 | Exclude | Smoking initiation | -0.00223 | 6.60E-09 |
| 227 | rs2094296 | Exclude | Diastolic blood pressure | 0.002122 | 5.70E-15 |
| 228 | rs603424 | Exclude | Cardiovascular disease | -0.00367 | 3.70E-35 |
| 229 | rs11190583 | Exclude | Diastolic blood pressure | 0.002556 | 1.10E-20 |
| 230 | rs1049466 | Exclude | Educational attainment | 0.003357 | 1.20E-10 |
| 231 | rs75398587 | Exclude | Educational attainment | -0.00865 | 2.60E-83 |
| 232 | rs10787429 | Exclude | Triglyceride levels | -0.00375 | 1.50E-49 |
| 233 | rs12414592 | Exclude | HDL cholesterol level | -0.0032 | 2.00E-28 |
| 234 | rs1966384 | Exclude | eGFR | 0.001985 | 2.20E-16 |
| 235 | rs67234429 | Exclude | Triglyceride levels | 0.001641 | 1.30E-10 |
| 236 | rs7901425 | keep | / | -0.00196 | 6.60E-13 |
| 237 | rs12277152 | keep | / | 0.009232 | 1.10E-68 |
| 238 | rs6421984 | Exclude | Creatinine levels | 0.00539 | 8.40E-124 |
| 239 | rs12281495 | keep | / | -0.00177 | 3.30E-15 |
| 240 | rs11601507 | Exclude | LDL cholesterol levels | 0.008285 | 3.30E-78 |
| 241 | rs11042106 | Exclude | Myocardial infarction | 0.003496 | 2.80E-29 |
| 242 | rs4757060 | Exclude | Smoking initiation | -0.00128 | 9.70E-10 |
| 243 | rs10430891 | Exclude | Systolic blood pressure | -0.00239 | 2.40E-18 |
| 244 | rs56074687 | Exclude | BMI | -0.00214 | 2.40E-18 |
| 245 | rs1228479 | Exclude | BMI | -0.00142 | 8.40E-10 |
| 246 | rs34467936 | Exclude | Systolic blood pressure | -0.00172 | 6.90E-13 |
| 247 | rs174564 | Exclude | Cholesterol | 0.005614 | 2.70E-130 |
| 248 | rs10897272 | Exclude | Waist-hip index | -0.00143 | 7.30E-10 |
| 249 | rs12270054 | Exclude | Cardiovascular diseases | -0.00168 | 3.70E-10 |
| 250 | rs498936 | Exclude | Total lipid levels in HDL | -0.00247 | 2.10E-16 |
| 251 | rs561778 | keep | / | 0.00163 | 2.40E-10 |
| 252 | rs7950422 | Exclude | Testosterone levels | -0.00175 | 9.90E-12 |
| 253 | rs648116 | Exclude | Body fat percentage | -0.0015 | 6.30E-09 |
| 254 | rs11216938 | Exclude | LDL cholesterol | -0.00382 | 7.30E-38 |
| 255 | rs240543 | keep | / | -0.00376 | 9.20E-38 |
| 256 | rs10893507 | Exclude | Coronary artery disease | -0.00732 | 1.00E-200 |
| 257 | rs80021729 | Exclude | BMI | 0.003494 | 1.10E-26 |
| 258 | rs12360937 | Exclude | BMI and adiposity | -0.00232 | 3.00E-12 |
| 259 | rs7966590 | Exclude | Body fat distribution | 0.006261 | 2.70E-162 |
| 260 | rs149363012 | Exclude | Thyroid levels | 0.007358 | 1.80E-26 |
| 261 | rs61909599 | Exclude | eGFR | -0.00316 | 3.50E-18 |
| 262 | rs2058877 | Exclude | Educational attainment | 0.001505 | 7.80E-11 |
| 263 | rs11048470 | Exclude | Waist-hip ratio | -0.00214 | 2.40E-16 |
| 264 | rs11050191 | Exclude | Total cholesterol levels | 0.00145 | 2.10E-10 |
| 265 | rs7132100 | Exclude | eGFR | 0.001759 | 2.40E-14 |
| 266 | rs930900 | Exclude | HDL levels | 0.003274 | 9.50E-42 |
| 267 | rs11609805 | Exclude | HDL levels | -0.0019 | 1.60E-12 |
| 268 | rs113479946 | keep | / | -0.00253 | 4.10E-08 |
| 269 | rs146787263 | exclude | Smoking initiation | -0.0017 | 6.90E-10 |
| 270 | rs17030285 | keep | / | 0.001862 | 2.10E-08 |
| 271 | rs2041894 | Exclude | BMI | -0.00233 | 4.60E-24 |
| 272 | rs666727 | Exclude | Diastolic blood pressure | -0.00407 | 9.20E-46 |
| 273 | rs79225028 | Exclude | Total cholesterol levels | 0.002199 | 1.60E-09 |
| 274 | rs7970695 | Exclude | Systolic blood pressure | -0.00702 | 1.00E-200 |
| 275 | rs11065593 | Exclude | systolic blood pressure | 0.00215 | 3.50E-08 |
| 276 | rs137889947 | Exclude | BMI | 0.004605 | 4.00E-09 |
| 277 | rs7998010 | keep | / | 0.001521 | 1.30E-10 |
| 278 | rs1170178 | Exclude | eGFR | -0.00225 | 5.70E-10 |
| 279 | rs58973023 | Exclude | Type 1 diabetes | 0.00598 | 1.70E-152 |
| 280 | rs151294127 | Exclude | No strong associations with ALP | -0.00232 | 7.50E-17 |
| 281 | rs665632 | keep | / | 0.00433 | 2.10E-49 |
| 282 | rs9565082 | exclude | Educational attainment | 0.002467 | 7.80E-27 |
| 283 | rs2298058 | Exclude | Triglycerides | 0.005091 | 6.40E-92 |
| 284 | rs7996702 | exclude | Coronary artery disease | -0.00279 | 1.60E-34 |
| 285 | rs9604045 | exclude | HDL cholesterol levels | -0.00172 | 1.50E-09 |
| 286 | rs6602909 | exclude | LDL cholesterol levels | 0.001667 | 1.70E-10 |
| 287 | rs34077103 | exclude | Calcium levels | -0.00176 | 1.60E-11 |
| 288 | rs6573778 | exclude | Testosterone levels | 0.00359 | 1.40E-54 |
| 289 | rs113670117 | Exclude | Systolic blood pressure | -0.00293 | 3.40E-12 |
| 290 | rs1276121 | Exclude | Systolic blood pressure | 0.001407 | 3.20E-09 |
| 291 | rs2239222 | Exclude | Testosterone levels | -0.00308 | 9.80E-40 |
| 292 | rs35322910 | Exclude | BMI | 0.001745 | 4.10E-10 |
| 293 | rs2012627 | Exclude | HDL cholesterol levels | -0.00194 | 1.30E-16 |
| 294 | rs75663481 | keep | / | 0.002535 | 2.60E-24 |
| 295 | rs73326585 | Keep | / | 0.002187 | 9.80E-12 |
| 296 | rs10151930 | Exclude | Waist circumference | -0.00147 | 1.50E-08 |
| 297 | rs117068593 | Exclude | Body fat percentage | -0.00298 | 1.10E-25 |
| 298 | rs28929474 | Exclude | Coronary artery disease | 0.014318 | 1.10E-71 |
| 299 | rs17101241 | Keep | / | 0.002478 | 2.20E-21 |
| 300 | rs141866277 | Exclude | No strong associations with ALP | -0.00893 | 6.70E-34 |
| 301 | rs2899472 | Exclude | Educational attainment | -0.00166 | 7.00E-11 |
| 302 | rs2043085 | Exclude | cholesterol levels | 0.004272 | 1.50E-73 |
| 303 | rs1077835 | Exclude | HDL | -0.00426 | 6.30E-57 |
| 304 | rs8041523 | Exclude | Smoking initiation | 0.001234 | 1.20E-08 |
| 305 | rs4842841 | Exclude | BMI | 0.001386 | 2.40E-08 |
| 306 | rs34239095 | Exclude | Blood pressure | -0.0023 | 6.90E-20 |
| 307 | rs71405688 | Exclude | eGFR | 0.002779 | 1.20E-16 |
| 308 | rs28441180 | Exclude | eGFR | 0.001387 | 7.10E-10 |
| 309 | rs9934839 | Exclude | Educational attainment | 0.001332 | 7.60E-09 |
| 310 | rs9926191 | Exclude | Dilated cardiomyopathy | -0.00549 | 1.40E-34 |
| 311 | rs444985 | Exclude | Systolic blood pressure | 0.001767 | 3.20E-11 |
| 312 | rs933574 | Exclude | Triglyceride levels | 0.002174 | 8.80E-23 |
| 313 | rs3198697 | Exclude | Cholesterol levels | -0.00143 | 2.70E-10 |
| 314 | rs8048601 | Exclude | Coronary artery disease | 0.001655 | 1.50E-10 |
| 315 | rs148702283 | Exclude | BMI | -0.00332 | 2.10E-18 |
| 316 | rs17616063 | Exclude | LDL levels | 0.002425 | 2.50E-09 |
| 317 | rs9932007 | Exclude | Type 2 diabetes | -0.00175 | 4.10E-15 |
| 318 | rs4122238 | Exclude | Ischemic stroke | 0.002347 | 1.10E-13 |
| 319 | rs9888986 | Exclude | HDL cholesterol levels | 0.003488 | 7.40E-20 |
| 320 | rs4788808 | Exclude | Educational attainment | -0.00271 | 1.50E-33 |
| 321 | rs9302635 | Exclude | Cholesterol levels | 0.005652 | 3.40E-83 |
| 322 | rs4575545 | Exclude | eGFR | 0.005709 | 6.00E-114 |
| 323 | rs6564719 | Exclude | eGFR | 0.001856 | 3.90E-11 |
| 324 | rs72791557 | Keep | / | -0.00192 | 2.70E-14 |
| 325 | rs904801 | Exclude | Total cholesterol levels | 0.001437 | 2.40E-08 |
| 326 | rs11078597 | Exclude | Testosterone levels | -0.0042 | 2.60E-44 |
| 327 | rs113461397 | Exclude | Coronary artery disease | 0.0041 | 7.50E-14 |
| 328 | rs3760352 | Exclude | Coronary artery disease | -0.00476 | 2.10E-77 |
| 329 | rs1222786 | Exclude | Total cholesterol levels | -0.00477 | 1.10E-95 |
| 330 | rs12449427 | Exclude | Total cholesterol levels | 0.008809 | 1.00E-200 |
| 331 | rs12150564 | Exclude | Educational attainment | -0.00131 | 1.20E-08 |
| 332 | rs62644890 | Exclude | Educational attainment | -0.00205 | 8.40E-12 |
| 333 | rs7502029 | Exclude | Smoking initiation | -0.00146 | 7.50E-09 |
| 334 | rs903504 | Exclude | Cardiovascular disease | -0.00378 | 2.90E-56 |
| 335 | rs16940845 | Exclude | BMI | 0.002157 | 3.20E-21 |
| 336 | rs62078384 | Exclude | HDL levels | 0.001727 | 4.50E-15 |
| 337 | rs60586860 | Keep | / | 0.007198 | 5.50E-28 |
| 338 | rs76708468 | Exclude | Sex | -0.00422 | 3.40E-12 |
| 339 | rs71378928 | Exclude | Coronary artery disease | -0.00305 | 3.90E-29 |
| 340 | rs113408695 | Exclude | High cholesterol | 0.003522 | 4.70E-10 |
| 341 | rs2670834 | Keep | / | -0.00334 | 2.80E-14 |
| 342 | rs4468717 | Exclude | Systolic blood pressure | -0.00297 | 3.10E-12 |
| 343 | rs2040189 | Exclude | Triglyceride levels | -0.00164 | 2.20E-13 |
| 344 | rs1943981 | Exclude | HDL cholesterol | -0.00253 | 8.70E-17 |
| 345 | rs117369509 | Exclude | Triglyceride levels | -0.00305 | 5.10E-10 |
| 346 | rs11152071 | Keep | / | -0.00322 | 5.70E-35 |
| 347 | rs884205 | Keep | / | -0.00487 | 2.00E-73 |
| 348 | rs11663201 | Exclude | eGFR | -0.00197 | 3.50E-15 |
| 349 | rs764036 | Exclude | eGFR | 0.002054 | 5.50E-18 |
| 350 | rs350832 | Exclude | HDL cholesterol | 0.002106 | 9.30E-14 |
| 351 | rs6510807 | Exclude | Kidney stone disease | 0.001323 | 9.20E-09 |
| 352 | rs78060698 | Keep | Kidney stone disease | 0.004436 | 1.20E-15 |
| 353 | rs12979056 | Exclude | Coronary artery disease | 0.001318 | 1.90E-09 |
| 354 | rs11554159 | Exclude | eGFR | -0.00249 | 4.10E-22 |
| 355 | rs58542926 | Exclude | Triglycerides | -0.01065 | 1.70E-134 |
| 356 | rs188247550 | Exclude | No strong associations with ALP | -0.01166 | 1.20E-30 |
| 357 | rs28570619 | Keep | / | 0.00722 | 3.80E-43 |
| 358 | rs78134016 | Exclude | Total cholesterol levels | -0.00229 | 2.20E-19 |
| 359 | rs34308190 | Keep | / | -0.00265 | 2.10E-08 |
| 360 | rs1672981 | Exclude | Body fat percentage | -0.00268 | 5.80E-10 |
| 361 | rs3865454 | Exclude | Smoking behaviour | -0.0052 | 4.50E-102 |
| 362 | rs5112 | Exclude | Cholesterol level | -0.00721 | 6.80E-189 |
| 363 | rs8102445 | Exclude | BMI | -0.00145 | 7.50E-12 |
| 364 | rs296393 | Exclude | Testosterone levels | -0.00274 | 1.20E-18 |
| 365 | rs2081194 | Exclude | Triglycerides in HDL | 0.00791 | 1.00E-200 |
| 366 | rs8736 | Exclude | No strong associations with ALP | -0.00572 | 8.00E-137 |
| 367 | rs11084326 | Keep | / | 0.001729 | 1.00E-08 |
| 368 | rs1172822 | Exclude | eGFR | 0.001307 | 5.40E-09 |
| 369 | rs573322136 | Exclude | Educational attainment | -0.00159 | 3.80E-08 |
| 370 | rs17721822 | Exclude | BMI | -0.00172 | 3.50E-12 |
| 371 | rs34952318 | Keep | / | 0.003994 | 4.20E-14 |
| 372 | rs3746786 | Exclude | BMI | 0.001572 | 1.70E-12 |
| 373 | rs2618566 | Exclude | High cholesterol | -0.00189 | 3.50E-15 |
| 374 | rs869358 | Exclude | HDL cholesterol levels | 0.005565 | 2.40E-134 |
| 375 | rs17259459 | Exclude | Triglyceride levels | -0.00166 | 1.90E-10 |
| 376 | rs2143877 | Exclude | Cholesterol levels | -0.00321 | 2.60E-39 |
| 377 | rs146496844 | Exclude | No strong associations with ALP | 0.006487 | 2.50E-12 |
| 378 | rs3918253 | Exclude | Cholesterol levels | 0.002434 | 1.00E-26 |
| 379 | rs111878400 | Keep | / | 0.004301 | 1.50E-14 |
| 380 | rs10888230 | Keep | / | 0.001814 | 7.00E-11 |
| 381 | rs7265084 | Exclude | BMI | 0.001475 | 1.60E-09 |
| 382 | rs3810502 | Exclude | Diastolic blood pressure | -0.00224 | 2.90E-14 |
| 383 | rs62219537 | Keep | / | 0.001414 | 3.20E-09 |
| 384 | rs2211688 | Keep | / | -0.00133 | 2.20E-08 |
| 385 | rs2834963 | Keep | / | -0.00194 | 2.40E-15 |
| 386 | rs2776288 | Exclude | eGFR | -0.00165 | 2.50E-13 |
| 387 | rs2836881 | Exclude | Coronary artery disease | -0.00242 | 3.60E-22 |
| 388 | rs35665085 | Exclude | Triglyceride levels | 0.003213 | 8.10E-12 |
| 389 | rs12165570 | Exclude | Diastolic blood pressure | -0.00163 | 4.00E-10 |
| 390 | rs13054904 | Keep | / | -0.0018 | 1.50E-13 |
| 391 | rs5751777 | Exclude | Testosterone levels | -0.00222 | 1.40E-22 |
| 392 | rs4822983 | Exclude | HDL levels | -0.00218 | 6.50E-19 |
| 393 | rs5999901 | Keep | / | 0.002352 | 2.40E-09 |
| 394 | rs132642 | Exclude | BMI | 0.003559 | 6.00E-30 |
| 395 | rs3788533 | Exclude | Triglycerides level | 0.001566 | 1.40E-12 |
| 396 | rs9611177 | Exclude | BMI | -0.00262 | 1.20E-27 |
| 397 | rs6008845 | Exclude | Sex | 0.001451 | 2.10E-09 |
| 398 | rs5771222 | Exclude | Type 2 diabetes | -0.00139 | 9.60E-11 |
| 399 | rs6009946 | Exclude | Educational attainment | 0.001558 | 9.50E-10 |

Abbreviations: ALP, alkaline phosphatase; LDL, low-density lipoprotein; HDL, high-density lipoprotein; CKD, chronic kidney disease; eGFR, estimated glomerular filtration rate; BMI, body mass index.

**Table S3. ALP quartiles, calcium, and phosphorus on CKM-cause and all-cause mortality.**

|  | CKM-cause mortality | | All-cause mortality | | |
| --- | --- | --- | --- | --- | --- |
| **Characteristic** | **HR**(**95% CI**) | **p-value** | **HR**(**95% CI**) | | **p-value** |
| **ALP Quartile** |  |  |  | |  |
| 1^st^ Quartile | Reference | | | Reference | |
| 2^nd^ Quartile | 1.56(1.01, 2.40) | ***0.043*** | 1.51(1.14, 2.01) | | ***0.004*** |
| 3^rd^ Quartile | 1.06(0.79, 1.42) | *0.7* | 1.41(1.12, 1.78) | | ***0.004*** |
| 4^th^ Quartile | 2.20(1.47, 3.28) | ***<0.001*** | 2.15(1.70, 2.70) | | ***<0.001*** |
| **Calcium** | 0.84(0.58, 1.22) | *0.4* | 0.74(0.56, 0.98) | | ***0.038*** |
| **Phosphorus** | 2.28(1.86, 2.79) | *<0.001* | 1.57(1.36, 1.83) | | ***<0.001*** |

This survey-weighted Cox proportional hazards model was adjusted by Age (years), Race and ethnicity, Poverty income ratio (PIR), Sex, BMI, Smoking status, Education, and vitamin D level.

Abbreviations: CKM, Cardiovascular-Kidney-Metabolic Syndrome; HRs, Hazards ratios; 95%CI, 95 confidence interval; BMI, body mass index.

**Table S4. Mortality rates of participants with different CRM stages.**

|  | All-cause deaths | All-cause cases | All-cause mortality rate | CKM-cause deaths | CKM-cause cases | CKM-cause mortality rate |
| --- | --- | --- | --- | --- | --- | --- |
| Stage 0 | 32 | 1746 | 1.8 | 1 | 1746 | 0.1 |
| Stage 1 | 62 | 3501 | 1.8 | 11 | 3501 | 0.3 |
| Stage 2 | 598 | 8270 | 7.2 | 155 | 8270 | 1.9 |
| Stage 3 | 62 | 186 | 33.3 | 21 | 186 | 11.3 |
| Stage 4a | 145 | 857 | 16.9 | 44 | 857 | 5.1 |
| Stage 4b | 224 | 673 | 33.3 | 112 | 673 | 16.6 |

**Table S5. Characteristics of GWAS datasets enrolled in the MR study.**

| **Items** | **GWAS ID** | **Consortium** | **Sample size** | **Population** |
| --- | --- | --- | --- | --- |
| **Serum ALP** | **ebi-a-GCST90025947** |  |  |  |
| **Cardiovascular disease** |  |  |  |  |
| **Chronic kidney disease** |  |  |  |  |
| **Diabetes** |  |  |  |  |

**Table S5. Heterogeneity and pleiotropy test for Mendelian analysis.**

|  | Method | Detected methods | ALP and CVD | CKD and ALP | ALP and T2DM |
| --- | --- | --- | --- | --- | --- |
| *P*-value of Heterogeneity | Cochran’s Q test | MR Egger | ***0.784*** | ***0.976*** | ***0.831*** |
|  |  | IVW | ***0.771*** | ***0.981*** | ***0.840*** |
| *P*-value of  Pleiotropy | MR-Egger intercept test |  | ***0.254*** | ***0.069*** | ***0.452*** |
