# Supplementary material for: Stage-specific associations of mineralization markers with CKM syndrome: Nationwide survey and genetic evidence for Alkaline phosphatase’s unique clinical role
Source: PLoS One. 2026 Jun 18;21(6):e0351946. doi: 10.1371/journal.pone.0351946 (PMC13278675; doi:10.1371/journal.pone.0351946)
Supplement: S2 Table — (DOCX) [file pone.0351946.s014.docx]

**Table S2. Characteristics of GWAS datasets enrolled in the MR study.**

| Items | GWAS ID | Consortium | Sample size | Population |
| --- | --- | --- | --- | --- |
| ALP | ebi-a-GCST90025947 | UKB | 437,896 | European ancestry |
| CVD | finn-b-I9_CVD_HARD | FinnGen | 147,212 | European ancestry |
| CKD | finn-b-N14_CHRONKIDNEYDIS | FinnGen | 216,743 | European ancestry |
| Diabetes | finn-b-T2D | FinnGen | 191,497 | European ancestry |

Abbreviations: CKD, chronic kidney disease; eGFR, estimated glomerular filtration rate; CVD, cardiovascular diseases; UKB, UK biobank.
