# Supplementary material for: Stage-specific associations of mineralization markers with CKM syndrome: Nationwide survey and genetic evidence for Alkaline phosphatase’s unique clinical role
Source: PLoS One. 2026 Jun 18;21(6):e0351946. doi: 10.1371/journal.pone.0351946 (PMC13278675; doi:10.1371/journal.pone.0351946)
Supplement: S4 Table — (DOCX) [file pone.0351946.s016.docx]

**Table S4. Mortality rates of participants with CKM stages(0-4b).**

| CKM stages | CKM-cause deaths | CKM-cause cases | CKM-cause mortality rate | All-cause deaths | All-cause cases | All-cause mortality rate |
| --- | --- | --- | --- | --- | --- | --- |
| Stage 0 | 1 | 1746 | 0.1% | 32 | 1746 | 1.8% |
| Stage 1 | 11 | 3501 | 0.3% | 62 | 3501 | 1.8% |
| Stage 2 | 155 | 8270 | 1.9% | 598 | 8270 | 7.2% |
| Stage 3 | 21 | 186 | 11.3% | 62 | 186 | 33.3% |
| Stage 4a | 44 | 857 | 5.1% | 145 | 857 | 16.9% |
| Stage 4b | 112 | 673 | 16.6% | 224 | 673 | 33.3% |

Abbreviations: CKM, Cardiovascular-Kidney-Metabolic Syndrome;
