# Supplementary material for: Stage-specific associations of mineralization markers with CKM syndrome: Nationwide survey and genetic evidence for Alkaline phosphatase’s unique clinical role
Source: PLoS One. 2026 Jun 18;21(6):e0351946. doi: 10.1371/journal.pone.0351946 (PMC13278675; doi:10.1371/journal.pone.0351946)
Supplement: S5 Table — (DOCX) [file pone.0351946.s017.docx]

**Table S5. Mortality rates of participants in non-advanced and advanced CKM stages.**

| CKM stages | All-cause deaths | All-cause cases | All-cause mortality rate | CKM-cause deaths | CKM-cause cases | CKM-cause mortality rate |
| --- | --- | --- | --- | --- | --- | --- |
| Non-advanced | 94 | 5247 | 1.8% | 12 | 5247 | 0.2% |
| Advanced | 1029 | 9986 | 10.3% | 332 | 9986 | 3.3% |

Abbreviations: CKM, Cardiovascular-Kidney-Metabolic Syndrome;
