# Supplementary material for: Stage-specific associations of mineralization markers with CKM syndrome: Nationwide survey and genetic evidence for Alkaline phosphatase’s unique clinical role
Source: PLoS One. 2026 Jun 18;21(6):e0351946. doi: 10.1371/journal.pone.0351946 (PMC13278675; doi:10.1371/journal.pone.0351946)
Supplement: S6 Table — (DOCX) [file pone.0351946.s018.docx]

**Table S6.** Survey-weighted multinomial logistic regression results for associations between ALP, Calcium, Phosphorus levels, and CKM stages 0-4b after excluding participants with liver diseases.

|  | ALP quartile | | Calcium (mg/dL) | | Phosphorus (mg/dL) | |
| --- | --- | --- | --- | --- | --- | --- |
| CKM Stages | RRR (95% CI) | *p*-value | RRR (95% CI) | *p*-value | RRR (95% CI) | *p*-value |
| Stage 0 | Reference |  | Reference |  | Reference |  |
| Stage 1 | 1.01 (1.00, 1.23) | ***0.056*** | 0.99 (0. 71, 1.38) | *0.952* | 0.92 (0. 77, 1.09) | *0. 324* |
| Stage 2 | 1.25 (1.13, 1.39) | ***<0.001*** | 2.11 (1.52, 2.94) | ***<0.001*** | 1.03 (0. 85, 1.24) | *0. 773* |
| Stage 3 | 2.08 (1.55, 2.79) | ***<0.001*** | 1.39 (0.63, 3.07) | *0.412* | 2.66 (1.65, 4.30) | ***<0.001*** |
| Stage 4a | 1.34 (1.18, 1.53) | ***<0.001*** | 1.54 (1.05, 2.25) | ***0.028*** | 1.05 (0.82, 1.36) | *0.683* |
| Stage 4b | 1.37 (1.16, 1.61) | ***<0.001*** | 1.69 (1.13, 2.51) | ***0.011*** | 1.75 (1.33, 2.31) | ***<0.001*** |

Adjusted by Age (years), Race and ethnicity, Poverty income ratio (PIR), Sex, BMI, Smoking status, Education, and vitamin D level.

Abbreviations: ORs, odds ratios; 95%CI, 95% confidence interval; CKM, Cardiovascular-Kidney-Metabolic Syndrome; BMI, body mass index.
