# Supplementary material for: Stage-specific associations of mineralization markers with CKM syndrome: Nationwide survey and genetic evidence for Alkaline phosphatase’s unique clinical role
Source: PLoS One. 2026 Jun 18;21(6):e0351946. doi: 10.1371/journal.pone.0351946 (PMC13278675; doi:10.1371/journal.pone.0351946)
Supplement: S7 Table — (DOCX) [file pone.0351946.s019.docx]

**Table S7.** The associations of ALP quartiles, phosphorus level, and calcium level with the likelihood of being classified into the advanced CKM stages after excluding the participants with liver diseases.

|  | Model 1 | | Model 2 | |
| --- | --- | --- | --- | --- |
| **Characteristic** | OR (95% CI) | *p*-value | OR (95% CI) | *p*-value |
| **ALP Quartile** |  |  |  |  |
| 1^st^ Quartile | Reference |  | Reference |  |
| 2^nd^ Quartile | 1.41 (1.22, 1.64) | ***<0.001*** | 1.17 (0.97, 1.40) | *0.093* |
| 3^rd^ Quartile | 1.75 (1.48, 2.05) | ***<0.001*** | 1.25 (1.01, 1.54) | ***0.040*** |
| 4^th^ Quartile | 2.73 (2.31, 3.23) | ***<0.001*** | 1.57 (1.31, 1.88) | ***<0.001*** |
| **Calcium (mg/dL)** | 1.54 (1.37, 1.72) | ***<0.001*** | 2.10 (1.74, 2.52) | ***<0.001*** |
| **Phosphorus (mg/dL)** | 0.91 (0.83, 0.99) | ***0.026*** | 1.10 (0.97, 1.25) | *0.13* |

Model 1: only ALP quartiles, Calcium (mg/dL), and Phosphorus (mg/dL), without adjustment.

Model 2: Model 1, adjusted by Age (years), Race and ethnicity, Poverty income ratio (PIR), Sex, BMI, Smoking status, Education, and vitamin D level.

Abbreviations: ORs, odds ratios; 95%CI, 95% confidence interval; CKM, Cardiovascular-Kidney-Metabolic Syndrome; BMI, body mass index.
