# Supplementary material for: Stage-specific associations of mineralization markers with CKM syndrome: Nationwide survey and genetic evidence for Alkaline phosphatase’s unique clinical role
Source: PLoS One. 2026 Jun 18;21(6):e0351946. doi: 10.1371/journal.pone.0351946 (PMC13278675; doi:10.1371/journal.pone.0351946)
Supplement: S8 Table — (DOCX) [file pone.0351946.s020.docx]

**Table S8.** Survey-weighted multinomial logistic regression results for associations between ALP, Calcium, Phosphorus levels, and CKM stages 0-4b, with the further adjustment of ALT, AST.

|  | ALP quartile | | Calcium (mg/dL) | | Phosphorus (mg/dL) | |
| --- | --- | --- | --- | --- | --- | --- |
| CKM Stages | RRR (95% CI) | *p*-value | RRR (95% CI) | *p*-value | RRR (95% CI) | *p*-value |
| Stage 0 | Reference |  | Reference |  | Reference |  |
| Stage 1 | 1.09 (0.99, 1.22) | ***0.096*** | 1.00 (0. 73, 1.38) | *0.994* | 0.91 (0. 76, 1.08) | *0. 324* |
| Stage 2 | 1.21 (1.09, 1.35) | ***0.001*** | 2.01 (1.47, 2.76) | ***<0.001*** | 1.01 (0. 84, 1.21) | *0. 936* |
| Stage 3 | 2.00 (1.50, 2.68) | ***<0.001*** | 1.48 (0.69, 3.20) | *0.309* | 2.79 (1.79, 4.33) | ***<0.001*** |
| Stage 4a | 1.27 (1.12, 1.45) | ***<0.001*** | 1.58 (1.11, 2.24) | ***0.012*** | 1.03 (0.81, 1.30) | *0.812* |
| Stage 4b | 1.35 (1.15, 1.58) | ***<0.001*** | 1.72 (1.18, 2.52) | ***0.006*** | 1.76 (1.35, 2.29) | ***<0.001*** |

Adjusted by Age (years), Race and ethnicity, Poverty income ratio (PIR), Sex, BMI, Smoking status, Education, and vitamin D level, ALT, AST.

Abbreviations: ORs, odds ratios; 95%CI, 95% confidence interval; CKM, Cardiovascular-Kidney-Metabolic Syndrome; BMI, body mass index; ALT, Alanine transaminase; Aspartate aminotransferase, AST.
