# Supplementary material for: Stage-specific associations of mineralization markers with CKM syndrome: Nationwide survey and genetic evidence for Alkaline phosphatase’s unique clinical role
Source: PLoS One. 2026 Jun 18;21(6):e0351946. doi: 10.1371/journal.pone.0351946 (PMC13278675; doi:10.1371/journal.pone.0351946)
Supplement: S9 Table — (DOCX) [file pone.0351946.s021.docx]

**Table S9.** The associations of ALP quartiles, phosphorus level, and calcium level with the likelihood of being classified into the advanced CKM stages, with the further adjustment of ALT, AST.

|  | Model 1 | | Model 2 | |
| --- | --- | --- | --- | --- |
| **Characteristic** | OR (95% CI) | *p*-value | OR (95% CI) | *p*-value |
| **ALP Quartile** |  |  |  |  |
| 1^st^ Quartile | Reference |  | Reference |  |
| 2^nd^ Quartile | 1.42 (1.23, 1.64) | ***<0.001*** | 1.16 (0.97, 1.39) | *0.093* |
| 3^rd^ Quartile | 1.76 (1.49, 2.07) | ***<0.001*** | 1.24 (1.01, 1.53) | ***0.040*** |
| 4^th^ Quartile | 2.72 (2.30, 3.22) | ***<0.001*** | 1.47 (1.22, 1.76) | ***<0.001*** |
| **Calcium (mg/dL)** | 1.53 (1.37, 1.72) | ***<0.001*** | 2.10 (1.74, 2.52) | ***<0.001*** |
| **Phosphorus (mg/dL)** | 0.90 (0.83, 0.98) | ***0.020*** | 1.09 (0.96, 1.23) | *0.20* |

Model 1: only ALP quartiles, Calcium (mg/dL), and Phosphorus (mg/dL), without adjustment.

Model 2: Model 1, adjusted by Age (years), Race and ethnicity, Poverty income ratio (PIR), Sex, BMI, Smoking status, Education, and vitamin D level, ALT, Alanine transaminase; Aspartate aminotransferase, AST.

Abbreviations: ORs, odds ratios; 95%CI, 95% confidence interval; CKM, Cardiovascular-Kidney-Metabolic Syndrome; BMI, body mass index.
