# Supplementary material for: Stage-specific associations of mineralization markers with CKM syndrome: Nationwide survey and genetic evidence for Alkaline phosphatase’s unique clinical role
Source: PLoS One. 2026 Jun 18;21(6):e0351946. doi: 10.1371/journal.pone.0351946 (PMC13278675; doi:10.1371/journal.pone.0351946)
Supplement: S10 Table — (DOCX) [file pone.0351946.s022.docx]

**Table S10.** Survey-weighted multinomial logistic regression results for associations of ALP Quartile, Calcium, and Phosphorus levels with CKM stages 0-4b with the further adjustment of ALT, AST, vitamin D supplement intake, and phosphate binder use.

|  | ALP Quartile | | Calcium (mg/dL) | | Phosphorus (mg/dL) | |
| --- | --- | --- | --- | --- | --- | --- |
| CKM Stages | RRR (95% CI) | *p*-value | RRR (95% CI) | *p*-value | RRR (95% CI) | *p*-value |
| Stage 0 | Reference |  | Reference |  | Reference |  |
| Stage 1 | 1.11 (0.97, 1.27) | *0.122* | 1.04 (0.68, 1.61) | *0.841* | 0.97 (0.81, 1.17) | *0.766* |
| Stage 2 | 1.25 (1.08, 1.44) | ***0.003*** | 1.96 (1.30, 2.94) | ***0.002*** | 1.09 (0.89, 1.34) | *0.379* |
| Stage 3 | 2.24 (1.61, 3.10) | ***<0.001*** | 1.21 (0.51, 2.89) | *0.655* | 3.48 (2.23, 5.43) | ***<0.001*** |
| Stage 4a | 1.32 (1.14, 1.53) | ***0.001*** | 1.50 (0.95, 2.39) | *0.084* | 1.17 (0.89, 1.53) | *0.259* |
| Stage 4b | 1.39 (1.16, 1.66) | ***0.001*** | 1.70 (1.08, 2.66) | ***0.022*** | 1.70 (1.26, 2.31) | ***0.001*** |

Adjustment: Age (years), Race and ethnicity, Poverty income ratio (PIR), Sex, BMI, Smoking status, Education, vitamin D level, ALT, AST, vitamin D supplement intake, and phosphate binder use.

Abbreviations: RRRs, relative risk ratios; 95%CI, 95 confidence interval; CKM, Cardiovascular-Kidney-Metabolic Syndrome; BMI, body mass index; ALT, Alanine transaminase; Aspartate aminotransferase, AST.
