# Supplementary material for: Stage-specific associations of mineralization markers with CKM syndrome: Nationwide survey and genetic evidence for Alkaline phosphatase’s unique clinical role
Source: PLoS One. 2026 Jun 18;21(6):e0351946. doi: 10.1371/journal.pone.0351946 (PMC13278675; doi:10.1371/journal.pone.0351946)
Supplement: S11 Table — (DOCX) [file pone.0351946.s023.docx]

**Table S11.** The associations of ALP quartiles, albumin-corrected calcium level, phosphorus level, and the likelihood of being classified into the advanced CKM stages with the further adjustment of ALT, AST, vitamin D supplement intake, and phosphate binder use.

|  | Model 1 | | Model 2 | |
| --- | --- | --- | --- | --- |
| **Characteristic** | OR (95% CI) | *p*-value | OR (95% CI) | *p*-value |
| **ALP Quartile** |  |  |  |  |
| 1^st^ Quartile | Reference |  | Reference |  |
| 2^nd^ Quartile | 1.48 (1.24, 1.77) | ***<0.001*** | 1.25 (1.00, 1.55) | ***0.045*** |
| 3^rd^ Quartile | 1.80 (1.53, 2.11) | ***<0.001*** | 1.29 (1.02, 1.63) | ***0.033*** |
| 4^th^ Quartile | 2.87 (2.42, 3.41) | ***<0.001*** | 1.52 (1.23, 1.87) | ***<0.001*** |
| **Calcium (mg/dL)** | 1.39 (1.19, 1.61) | ***<0.001*** | 1.88 (1.51, 2.34) | ***<0.001*** |
| **Phosphorus (mg/dL)** | 0.92 (0.83, 1.02) | *0.100* | 1.12 (0.97, 1.29) | *0.13* |

Model 1: only ALP quartiles, Calcium (mg/dL), and Phosphorus (mg/dL), without adjustment.

Model 2: Model 1, adjusted by Age (years), Race and ethnicity, Poverty income ratio, Sex, BMI, Smoking status, Education, vitamin D level, ALT, AST, vitamin D supplement intake, and phosphate binder use.

Abbreviations: ORs, odds ratios; 95%CI, 95% confidence interval; CKM, Cardiovascular-Kidney-Metabolic Syndrome; BMI, body mass index; ALT, Alanine transaminase; Aspartate aminotransferase, AST.
