# Supplementary material for: Stage-specific associations of mineralization markers with CKM syndrome: Nationwide survey and genetic evidence for Alkaline phosphatase’s unique clinical role
Source: PLoS One. 2026 Jun 18;21(6):e0351946. doi: 10.1371/journal.pone.0351946 (PMC13278675; doi:10.1371/journal.pone.0351946)
Supplement: S12 Table — (DOCX) [file pone.0351946.s024.docx]

**Table S12.** Survey-weighted multinomial logistic regression results for associations between ALP, Albumin-corrected calcium, Phosphorus levels, and CKM stages 0-4b, with the further adjustment of ALT, AST.

|  | ALP quartile | | Corrected calcium (mg/dL) | | Phosphorus (mg/dL) | |
| --- | --- | --- | --- | --- | --- | --- |
| CKM Stages | RRR (95% CI) | *p*-value | RRR (95% CI) | *p*-value | RRR (95% CI) | *p*-value |
| Stage 0 | Reference |  | Reference |  | Reference |  |
| Stage 1 | 1.10 (0.99, 1.23) | ***0.083*** | 0.92 (0. 64, 1.32) | *0.656* | 0.91 (0. 76, 1.08) | *0. 262* |
| Stage 2 | 1.21 (1.08, 1.34) | ***0.001*** | 1.62 (1.20, 2.20) | ***<0.001*** | 1.01 (0. 88, 1.29) | *0. 510* |
| Stage 3 | 1.93 (1.45, 2.57) | ***<0.001*** | 4.46 (2.37, 8.39) | ***<0.001*** | 2.87 (1.74, 4.74) | ***<0.001*** |
| Stage 4a | 1.27 (1.12, 1.44) | ***<0.001*** | 1.52 (1.00, 2.31) | ***0.048*** | 1.07 (0.84, 1.35) | *0.599* |
| Stage 4b | 1.33 (1.13, 1.56) | ***0.001*** | 2.33 (1.54, 3.53) | ***<0.001*** | 1.84 (1.41, 2.41) | ***<0.001*** |

Adjusted by Age (years), Race and ethnicity, Poverty income ratio (PIR), Sex, BMI, Smoking status, Education, and vitamin D level, ALT, AST.

Abbreviations: ORs, odds ratios; 95%CI, 95% confidence interval; CKM, Cardiovascular-Kidney-Metabolic Syndrome; BMI, body mass index; ALT, Alanine transaminase; Aspartate aminotransferase, AST.
