# Supplementary material for: Stage-specific associations of mineralization markers with CKM syndrome: Nationwide survey and genetic evidence for Alkaline phosphatase’s unique clinical role
Source: PLoS One. 2026 Jun 18;21(6):e0351946. doi: 10.1371/journal.pone.0351946 (PMC13278675; doi:10.1371/journal.pone.0351946)
Supplement: S13 Table — (DOCX) [file pone.0351946.s025.docx]

**Table S13.** The associations of ALP quartiles, albumin-corrected calcium level, phosphorus level, and the likelihood of being classified into the advanced CKM stages.

|  | Model 1 | | | Model 2 | |
| --- | --- | --- | --- | --- | --- |
| **Characteristic** | | OR (95% CI) | *p*-value | OR (95% CI) | *p*-value |
| **ALP Quartile** | |  |  |  |  |
| 1^st^ Quartile | | Reference |  | Reference |  |
| 2^nd^ Quartile | | 1.38 (1.19, 1.60) | ***<0.001*** | 1.15 (0.95, 1.37) | *0.14* |
| 3^rd^ Quartile | | 1.68 (1.43, 1.97) | ***<0.001*** | 1.23 (1.00, 1.50) | ***0.048*** |
| 4^th^ Quartile | | 2.40 (2.04, 2.83) | ***<0.001*** | 1.41 (1.19, 1.68) | ***<0.001*** |
| **Corrected calcium (mg/dL)** | | 1.53 (1.37, 1.72) | ***0.006*** | 1.14 (1.01, 1.29) | ***0.032*** |
| **Phosphorus (mg/dL)** | | 0.90 (0.83, 0.98) | ***<0.001*** | 1.74 (1.51, 2.00) | ***<0.001*** |

Model 1: only ALP quartiles, Calcium (mg/dL), and Phosphorus (mg/dL), without adjustment.

Model 2: Model 1, adjusted by Age (years), Race and ethnicity, Poverty income ratio (PIR), Sex, BMI, Smoking status, Education, and vitamin D level, ALT, Alanine transaminase; Aspartate aminotransferase, AST.

Abbreviations: ORs, odds ratios; 95%CI, 95% confidence interval; CKM, Cardiovascular-Kidney-Metabolic Syndrome; BMI, body mass index.
