# Supplementary material for: Stage-specific associations of mineralization markers with CKM syndrome: Nationwide survey and genetic evidence for Alkaline phosphatase’s unique clinical role
Source: PLoS One. 2026 Jun 18;21(6):e0351946. doi: 10.1371/journal.pone.0351946 (PMC13278675; doi:10.1371/journal.pone.0351946)
Supplement: S14 Table — (DOCX) [file pone.0351946.s026.docx]

**Table S14. ALP quartiles, calcium, albumin-corrected calcium, and phosphorus on CKM-cause and all-cause mortality.**

|  | CKM-cause mortality | | | All-cause mortality | | | |
| --- | --- | --- | --- | --- | --- | --- | --- |
| **Characteristic** | | **HR**(**95% CI**) | **p-value** | | | **HR**(**95% CI**) | **p-value** |
| **ALP Quartile** | |  |  | | |  |  |
| 1^st^ Quartile | | Reference | | | Reference | | |
| 2^nd^ Quartile | | 1.56(1.01, 2.40) | ***0.043*** | | | 1.51(1.14, 2.01) | ***0.004*** |
| 3^rd^ Quartile | | 1.06(0.79, 1.42) | *0.700* | | | 1.41(1.12, 1.78) | ***0.004*** |
| 4^th^ Quartile | | 2.20(1.47, 3.28) | ***<0.001*** | | | 2.15(1.70, 2.70) | ***<0.001*** |
| **Calcium** | | 0.84(0.58, 1.22) | *0.400* | | | 0.74(0.56, 0.98) | ***0.038*** |
| **Corrected calcium** | | 1.37(0.90, 2.10) | *0.140* | | | 1.34(1.02, 1.76) | ***0.037*** |
| **Phosphorus** | | 2.28(1.86, 2.79) | ***<0.001*** | | | 1.57(1.36, 1.83) | ***<0.001*** |

This survey-weighted Cox proportional hazards model was adjusted by Age (years), Race and ethnicity, Poverty income ratio (PIR), Sex, BMI, Smoking status, Education, and vitamin D level.

Abbreviations: CKM, Cardiovascular-Kidney-Metabolic Syndrome; HRs, Hazards ratios; 95%CI, 95 confidence interval; BMI, body mass index.
