# Supplementary material for: Stage-specific associations of mineralization markers with CKM syndrome: Nationwide survey and genetic evidence for Alkaline phosphatase’s unique clinical role
Source: PLoS One. 2026 Jun 18;21(6):e0351946. doi: 10.1371/journal.pone.0351946 (PMC13278675; doi:10.1371/journal.pone.0351946)
Supplement: S15 Table — (DOCX) [file pone.0351946.s027.docx]

**Table S15. Heterogeneity and pleiotropy test for Mendelian analysis.**

|  | Detected methods | ALP and CVD | CKD and ALP | ALP andT2DM |
| --- | --- | --- | --- | --- |
| *P*-value of Heterogeneity | MR Egger | ***0.994*** | ***0.976*** | ***0.831*** |
|  | IVW | ***0.994*** | ***0.981*** | ***0.840*** |
| *P*-value of Pleiotropy |  | ***0.359*** | ***0.069*** | ***0.452*** |

Abbreviations: CKM, Cardiovascular-Kidney-Metabolic Syndrome; CKD, chronic kidney disease; eGFR, estimated glomerular filtration rate; ACR, Urine albumin to creatinine ratio; BMI, body mass index.
