# Supplementary material for: Stage-specific associations of mineralization markers with CKM syndrome: Nationwide survey and genetic evidence for Alkaline phosphatase’s unique clinical role
Source: PLoS One. 2026 Jun 18;21(6):e0351946. doi: 10.1371/journal.pone.0351946 (PMC13278675; doi:10.1371/journal.pone.0351946)
Supplement: Attachment — Submitted filename: Comment for PLOS ONE 45261.docx [file pone.0351946.s028.docx]

**Dear the Editor of the PLOS ONE Journal,**

Thank you very much for providing me with this opportunity. I would also like to sincerely acknowledge the authors for their valuable contributions to the scientific community and for advancing knowledge in their respective field.

**Manuscript Number:** PONE-D-25-45261

**Article Type:** Research Article

**Title:** Stage-Specific Associations of Mineralization Markers with CKM Syndrome: Nationwide Survey and Genetic Evidence for Alkaline Phosphatase's Unique Clinical Role

- My overall evaluation of the manuscript is that the manuscript addresses an important and timely topic: the role of mineralization markers (ALP, calcium, and phosphorus) across stages of Cardiovascular–Kidney–Metabolic (CKM) syndrome using a large, nationally representative dataset (NHANES), complemented by Mendelian randomization analyses. The combination of stage-specific epidemiological analysis, mortality outcomes, and genetic causal inference is a notable strength. The study is generally well designed and statistically sophisticated, and the findings particularly the consistent association of ALP with CKM stages and mortality are potentially clinically relevant. However, several methodological clarifications, interpretational refinements, and presentation improvements are required to strengthen the scientific validity and transparency of the work.

**Major Comments**

**Conceptual clarity of CKM staging and causal interpretation**

- The manuscript applies modified AHA CKM staging using NHANES variables, which is reasonable; however, **CKM stages are composite constructs** incorporating outcomes (CVD, CKD, diabetes) that are also biologically linked to ALP, calcium, and phosphorus. This creates a risk of **circularity or over interpretation,** particularly when associations are described as “risk” of CKM stages in a cross-sectional design. What I recommend is that the authors are expected to clarify throughout manuscript that the primary analyses are **associational**, not predictive and replace phrasing such as “risk of CKM stage” with **“likelihood of being classified into CKM stage”** where appropriate and explicitly discuss potential **bidirectional relationships** between ALP and CKM components in the discussion.

**Study participants**

- Participants under 20 years of age or those who were pregnant were excluded. Additionally, individuals with insufficient data to evaluate CKM staging were excluded. According to WHO, age of 18 years and above is considered as an adult, what is your justification to exclude participants under 20 years old? In addition, individuals with insufficient data were excluded from the study. What percentage or which data level was considered insufficient?

**Cross-sectional design vs. causal inference**

- While the Mendelian randomization (MR) analysis strengthens causal inference for ALP CVD and CKD ALP relationships, the **NHANES analyses remain cross-sectional.** Some interpretations in the discussion imply disease progression or temporality that cannot be established. Therefore, clearly distinguish between cross-sectional NHANES findings (association), prospective mortality analyses (temporal but limited), and MR findings (genetic causality) and avoid implying that ALP “drives progression” of CKM stages without longitudinal CKM staging data.

**Use and interpretation of ALP as a non-specific biomarker**

- Total serum ALP includes **hepatic, bone, intestinal, and placental isoenzymes,** yet the manuscript interprets ALP largely as a calcification/mineralization marker. Liver disease, cholestasis, and subclinical hepatic dysfunction may confound associations. Thus, explicitly acknowledge the **non-specific nature of total ALP** and discuss potential confounding from liver-related ALP sources. If available, clarify whether AST/ALT or liver disease variables were considered or why they were not included.

**Calcium findings: inconsistency across stages and outcomes**

- Calcium shows positive association with advanced CKM stages, non-linear associations in RCS models, and inverse association with all-cause mortality. These findings appear **biologically inconsistent** and require clearer explanation. Therefore, expand discussion on tight homeostatic regulation of serum calcium, influence of albumin (uncorrected calcium limitation), CKD-related hypocalcemia vs. CVD-related hypercalcemia and clarify whether **albumin-corrected calcium** was considered and justify its exclusion.

**Phosphorus: contradictory associations across analyses**

- Phosphorus is associated with stage 3 and 4b in multinomial models, not associated with advanced CKM after adjustment, and strongly associated with mortality. This pattern suggests **context-specific effects,** primarily driven by CKD severity. Thus, explicitly frame phosphorus as a **late-stage CKD marker** rather than a general CKM biomarker and emphasize that phosphorus may be more relevant to **mortality risk** than CKM stage classification.

**Mendelian randomization: instrument selection and interpretation**

- The MR analysis is a strength, but removing >80% of ALP-associated SNPs raises concerns about **instrument strength and selection bias**. The MR-Egger results are largely non-significant. The authors are recommended to provide F-statistics or mean F-statistics to demonstrate instrument strength, discuss the discrepancy between IVW and MR-Egger results more cautiously and avoid strong causal language where MR evidence is borderline or inconsistent.

**Minor Comments**

- Several sentences require grammatical polishing (e.g., “To addressing this gap,” “This was because the relative risk ratios…”).
- Use consistent terminology for: “advanced CKM stages” vs. “advanced CKM”, “CKM-cause mortality” vs. “CKM-related mortality”
- Clearly state whether calcium and phosphorus RRRs are per 1 mmol/L, 1 mg/dL, or per SD increase.
- Ensure consistent use of OR vs. RRR vs. HR.
- Figures showing RCS curves should clearly label reference values.
- Strengths of the Study
- Large, nationally representative sample.
- Comprehensive CKM staging framework.
- Robust survey-weighted statistical methods.
- Integration of mortality analysis.
- Use of Mendelian randomization to explore causality.
- Clear evidence supporting ALP as a consistent CKM-associated biomarker.

**Overall Recommendation**

**Major revision**

The manuscript is scientifically valuable and suitable for publication after **conceptual clarification, cautious interpretation of causality, and improved discussion of biomarker specificity and biological mechanisms.** Addressing the points above will significantly enhance the rigor, clarity, and impact of the study.
