# Supplementary material for: Stage-specific associations of mineralization markers with CKM syndrome: Nationwide survey and genetic evidence for Alkaline phosphatase’s unique clinical role
Source: PLoS One. 2026 Jun 18;21(6):e0351946. doi: 10.1371/journal.pone.0351946 (PMC13278675; doi:10.1371/journal.pone.0351946)
Supplement: Attachment — Submitted filename: Response to reviewers.docx [file pone.0351946.s030.docx]

Dear Editor,

We are submitting a revised version of our manuscript (Manuscript ID: PONE-D-25-45261), ‘Stage-Specific Associations of Mineralization Markers with CKM Syndrome: Nationwide Survey and Genetic Evidence for Alkaline Phosphatase's Unique Clinical Role’, for further consideration at ***PLOS One***.

Understanding the mechanisms of Cardiovascular-Kidney-Metabolic (CKM) syndrome in developing novel diagnostic markers and therapeutic strategies, but the role of calcification markers in CKM stages remains unclear. Our manuscript shows that ALP may serve as a marker for CKM syndrome. While calcium showed stage-specific associations and phosphorus was less informative, ALP demonstrated consistent linear relationships across CKM stages. The reviewers responded that this study is “statistically sophisticated” and also “potentially clinically relevant”, some refinements should be addressed.

We revised our data analysis and clarification, addressed (where possible) all reviewer comments, and our revised manuscript is substantially stronger after completing this round of revisions. In this revised version, changes to our manuscript were all highlighted using track changes. All Reviewer comments have been addressed and are outlined in detail in our Response to Reviewer and attached as a Word file.

We hope our work is of sufficiently high quality to warrant further consideration for publication in ***PLOS One***.

Thank you for serving as the editor for our manuscript, and please feel free to contact me with any questions.

Sincerely yours,

Dr. Liangru Shen

**Reviewer #1:**

**Comment:** My overall evaluation of the manuscript is that the manuscript addresses an important and timely topic: the role of mineralization markers (ALP, calcium, and phosphorus) across stages of Cardiovascular–Kidney–Metabolic (CKM) syndrome using a large, nationally representative dataset (NHANES), complemented by Mendelian randomization analyses. The combination of stage-specific epidemiological analysis, mortality outcomes, and genetic causal inference is a notable strength. The study is generally well designed and statistically sophisticated, and the findings particularly the consistent association of ALP with CKM stages and mortality are potentially clinically relevant. However, several methodological clarifications, interpretational refinements, and presentation improvements are required to strengthen the scientific validity and transparency of the work.

**Response:** Thank you again for your positive comments and valuable suggestions to improve the quality of our manuscript. Thank you for the enthusiasm!!!!!

**Major Comments**

**Conceptual clarity of CKM staging and causal interpretation**

**Comment:** The manuscript applies modified AHA CKM staging using NHANES variables, which is reasonable; however, CKM stages are composite constructs incorporating outcomes (CVD, CKD, diabetes) that are also biologically linked to ALP, calcium, and phosphorus. This creates a risk of circularity or over interpretation, particularly when associations are described as “risk” of CKM stages in a cross-sectional design. What I recommend is that the authors are expected to clarify throughout manuscript that the primary analyses are associational, not predictive and replace phrasing such as “risk of CKM stage” with “likelihood of being classified into CKM stage” where appropriate and explicitly discuss potential bidirectional relationships between ALP and CKM components in the discussion.

**Response:** Thank you for pointing this out!! We feel very grateful for your professional review work on our article. As you are concerned, using “risk of CKM stage” or “risk” term to describe the results in a cross-sectional study is not appropriate. According to your nice suggestions, we have made extensive corrections to our previous draft and replaced the“risk of CKM stage” with “likelihood of being classified into CKM stage” and also avoided using “risk” to describe our current results.

**Study participants**

**Comment:** Participants under 20 years of age or those who were pregnant were excluded. Additionally, individuals with insufficient data to evaluate CKM staging were excluded. According to WHO, age of 18 years and above is considered as an adult, what is your justification to exclude participants under 20 years old?

**Response:** Thank you for the your kind and constructive suggestions to improve the quality of our manuscript. We apologize for these confusions we’ve caused. The reason why we excluded participants aged below 20 years old was that some of the demographic data, such as `marital status` and `education level` was only available among participants above 20 years old to avoid the potential disclosure risks. Also, among the `anthropometric data`, different BMI calculation formulas were used for participants below 20 years old. Therefore, for consistency throughout the study, we only included adults above 20 years old instead of using 18 years old as one of the inclusion criterion.

To avoid possible confusion for the readers, we also included the explanation in the revised manuscript.

*The reason why we excluded participants aged below 20 years old instead of 18 years old was that some of the demographic data in the NHANES survey, such as `marital status` and `education level`, was only available among participants above 20 years old to avoid the potential disclosure risks.* (**Materials and Methods section, lines 28-31, page 4**)

**Comment:** In addition, individuals with insufficient data were excluded from the study. What percentage or which data level was considered insufficient?

**Response:** We appreciate your thought-provoking questions regarding the definition of `insufficient data`, which we did not clarify in the previous manuscript. In our current study, calcification markers (ALP, calcium, phosphorus) and CKM stages were necessary variables to perform analyses. Participants with missing data in calcification markers and CKM stage classifications were thus excluded from the cohort. Therefore, no certain percentage was defined for insufficient data.

We strengthened the description for the missing data as follows:

*Specifically, those missing information on major CVD events, estimated glomerular filtration rate (eGFR), diabetes status, body mass index (BMI), or cholesterol levels,* *since these are key parameters for determining CKM stage. Participants lacking serum ALP, calcium, or phosphorus measurements were also excluded* *to ensure the exposure data were available.* (**Materials and Methods section, lines 22-25, page 4**).

And we also reported the overall percentage of the missing confounders:

*The overall percentage of missing confounders in our study varied from 3.5% to 9.7%.* (**Results section, lines 11-12, page 9**).

**Cross-sectional design vs. causal inference**

**Comment:** While the Mendelian randomization (MR) analysis strengthens causal inference for ALP CVD and CKD ALP relationships, the NHANES analyses remain cross-sectional. Some interpretations in the discussion imply disease progression or temporality that cannot be established. Therefore, clearly distinguish between cross-sectional NHANES findings (association), prospective mortality analyses (temporal but limited), and MR findings (genetic causality), and avoid implying that ALP “drives progression” of CKM stages without longitudinal CKM staging data.

**Response:** Your comprehensive and constructive comments and suggestions are instrumental in elevating the accuracy of our manuscript. We totally agree with you that no longitudinal CKM staging data were analyzed in the current study. The sentences regarding ALP “drives progression” of CKM stages were therefore extensively revised or removed in the discussion section to prevent misleading the readers or overinterpreting the results.

**Use and interpretation of ALP as a non-specific biomarker**

**Comment:** Total serum ALP includes hepatic, bone, intestinal, and placental isoenzymes, yet the manuscript interprets ALP largely as a calcification/mineralization marker. Liver disease, cholestasis, and subclinical hepatic dysfunction may confound associations. Thus, explicitly acknowledge the non-specific nature of total ALP and discuss potential confounding from liver-related ALP sources.

**Response:** Thank you for this critical comment regarding the non-specific nature of total ALP. Admittedly, there are 4 isoenzymes of ALP and many isoforms generated from these 4 isoenzymes, as you mentioned above. However, we also wish to highlight a physiological point that supports the biological plausibility of our interpretations. As described by Haarhaus et al. (2017), bone and liver tissue-nonspecific ALP (TNALP) are the most abundant isoforms of ALP, comprising more than 90% of total serum ALP activity in a 1:1 ratio in serum. We therefore interpreted ALP largely as a calcification/mineralization marker, neglecting the potential confounding effects from the liver TNALP, as you pointed out.

To address the concern raised by the reviewer as much as possible, we first performed the sensitivity analysis by excluding all the participants with a self-report of `still having a liver condition (including fatty liver, liver fibrosis, liver cirrhosis, viral hepatitis, autoimmune hepatitis, other liver disease)`. As a result, 341 participants were excluded from the current cohort, and conclusions regarding serum ALP, calcium, and phosphorus remain consistent after full adjustment in both multinomial and binomial logistic regression analysis (**Table S6-S7**).

We also describe the updated analysis in the revised manuscript:

*Except for bone ALP, liver ALP is another source abundant in serum. We therefore performed the sensitivity analysis by excluding all the participants with a self-report of `still having a liver condition (including fatty liver, liver fibrosis, liver cirrhosis, viral hepatitis, autoimmune hepatitis, other liver disease)`, adjusting for all the variables aforementioned.* (**Materials and Methods section, lines 14-19, page 8**).

*To avoid the potential confounding effects of liver diseases on ALP, the sensitivity analyses of multinomial and binomial logistic regressions were performed by excluding the participants with liver diseases. As a result, 341 participants were excluded from the current cohort, and conclusions regarding serum ALP, calcium, and phosphorus remained consistent in both multinomial and binomial logistic regression analysis (****Table*** ***S6-S7****), indicating the stability of the results.* (**Results section, lines 27-32, page 10**).

***Table S6.*** *Survey-weighted multinomial logistic regression results for associations between ALP, Calcium, Phosphorus levels, and CKM stages 0-4b after excluding participants with liver diseases.*

*Adjusted by Age (years), Race and ethnicity, Poverty income ratio (PIR), Sex, BMI, Smoking status, Education, and vitamin D level.*

*Abbreviations: ORs, odds ratios; 95%CI, 95% confidence interval; CKM, Cardiovascular-Kidney-Metabolic Syndrome; BMI, body mass index.*

***Table S7.*** *The associations of ALP quartiles, phosphorus level, and calcium level with the likelihood of being classified into the advanced CKM stages after excluding the participants with liver diseases.*

*Model 1: only ALP quartiles, Calcium (mg/dL), and Phosphorus (mg/dL), without adjustment.*

*Model 2: Model 1, adjusted by Age (years), Race and ethnicity, Poverty income ratio (PIR), Sex, BMI, Smoking status, Education, and vitamin D level.*

*Abbreviations: ORs, odds ratios; 95%CI, 95% confidence interval; CKM, Cardiovascular-Kidney-Metabolic Syndrome; BMI, body mass index.*

In the discussion section, we further acknowledged the non-specific nature of total ALP and discussed potential confounding from liver-related ALP sources according to your constructive suggestions:

*However, serum ALP was comprised of hepatic, bone, intestinal, and placental isoenzymes, and bone and liver isoforms account for over 90% of total serum ALP activity [34]. Although our further sensitivity analysis demonstrated consistent results by excluding the participants with liver diseases and further adjusting ALT as well as AST, we could not diminish the confounding effects of subclinical hepatic dysfunction.* (**Discussion section, lines 5-10, page 15**).

*[Reference]*

*34.	Haarhaus M, Brandenburg V, Kalantar-Zadeh K, Stenvinkel P, Magnusson P. Alkaline Phosphatase: A Novel Treatment Target for Cardiovascular Disease in CKD. Nat Rev Nephrol (2017) 13(7):429-42. Epub 20170515. doi: 10.1038/nrneph.2017.60.*

*Furthermore, we could not differentiate the bone ALP and liver ALP since they are both abundant in serum. Hence, further mechanism research needs to be performed to determine which ALP is more significantly associated with the CKM syndrome.* (**Discussion section, lines 17-19, page 17**).

**Comment:** If available, clarify whether AST/ALT or liver disease variables were considered or why they were not included.

**Response:** We sincerely appreciated your thought-provoking comments concerning the availability of variables related to liver functions. Indeed, we agreed that AST/ALT are appropriate adjustments for the potential confounding effects caused by the dysfunctional liver on ALP. Therefore, we further adjusted serum AST/ALT levels in multinomial and binomial logistic regressions and the results were updated in **Table S8-S9**, revealing the same conclusions on ALP, calcium, and phosphorus in CKM stages.

We also added the above content in the revised manuscript:

*Additionally, to further exclude the potential confounding effects of the dysfunctional liver, serum alanine transaminase (ALT) and aspartate aminotransferase (AST) were additionally adjusted.* (**Materials and Methods section**, **lines 17-19, page 8**).

*Moreover, we further adjusted serum ALT and AST in the multinomial and binomial logistic regressions,* *revealing the same conclusions on ALP, calcium, and phosphorus in CKM stages (****Table S8-S9****).* (**Results section**, **lines 32-34, page 10**).

***Table S8.*** *Survey-weighted multinomial logistic regression results for associations between ALP, Calcium, Phosphorus levels, and CKM stages 0-4b, with the further adjustment of ALT, AST.*

*Adjusted by Age (years), Race and ethnicity, Poverty income ratio (PIR), Sex, BMI, Smoking status, Education, and vitamin D level, ALT, AST.*

*Abbreviations: ORs, odds ratios; 95%CI, 95% confidence interval; CKM, Cardiovascular-Kidney-Metabolic Syndrome; BMI, body mass index; ALT, Alanine transaminase; Aspartate aminotransferase, AST.*

***Table S9****. The associations of ALP quartiles, phosphorus level, and calcium level with the likelihood of being classified into the advanced CKM stages, with the further adjustment of ALT, AST.*

*Model 1: only ALP quartiles, Calcium (mg/dL), and Phosphorus (mg/dL), without adjustment.*

*Model 2: Model 1, adjusted by Age (years), Race and ethnicity, Poverty income ratio (PIR), Sex, BMI, Smoking status, Education, and vitamin D level, ALT, Alanine transaminase; Aspartate aminotransferase, AST.*

*Abbreviations: ORs, odds ratios; 95%CI, 95% confidence interval; CKM, Cardiovascular-Kidney-Metabolic Syndrome; BMI, body mass index.*

Additionally, liver disease variables could be obtained only via self-reported questionnaires, which we used for sensitivity analysis.

**Calcium findings: inconsistency across stages and outcomes**

**Comment:** Calcium shows positive association with advanced CKM stages, non-linear associations in RCS models, and inverse association with all-cause mortality. These findings appear biologically inconsistent and require clearer explanation. Therefore, expand discussion on tight homeostatic regulation of serum calcium, influence of albumin (uncorrected calcium limitation), CKD-related hypocalcemia vs. CVD-related hypercalcemia

**Response:** We sincerely thank you for your valuable feedback regarding calcium levels in CKM syndrome that we have used to improve the quality of our manuscript. As you suggested, we have expanded the discussion to explain the inconsistency existing in the associations of calcium with CKM stages and with all-cause mortality.

The discussion we expanded was as follows:

*Hypoalbuminemia, a common disturbance in hospitalized patients, has been reported to mask the concentration of increased calcium among the critically ill patients [27], which was also observed among the current cohort, where CKM stage 0 exhibited the highest serum albumin concentrations. Also, CKD-related ‘masked’ hypocalcemia might be partially explained by this phenomenon, in which ionized calcium (active form) concentration can be normal or even higher but the total calcium level is lower due to the reduced albumin-bound calcium [40]. Hence, our biologically inconsistent results between positive associations of calcium level with CKM stages and its inverse association with all-cause mortality since end-stage participants tended to have masked hypocalcemia.* (**Discussion section**, **lines 30-34, page 15; Discussion section**, **lines 1-4, page 16**).

*[Reference]*

*27.	Figge J, Jabor A, Kazda A, Fencl V. Anion Gap and Hypoalbuminemia. Crit Care Med (1998) 26(11):1807-10. doi: 10.1097/00003246-199811000-00019.*

**Comment:** And clarify whether albumin-corrected calcium was considered and justify its exclusion.

**Response:** We sincerely appreciated your professional comments and advice. As the concern you mentioned above, in the revised manuscript, further replacement with albumin-corrected calcium levels in the associations with CMK stages and CKM-caused, all-cause mortality was performed to validate the rationality of the explanations. Consequently, in fully adjusted models, albumin-corrected calcium demonstrated significant positive associations with both CKM stage 2-4b (**Table S10**) and all-cause mortality risk (**Table S11**).

The updated part was also inserted in the revised manuscript:

*Since serum albumin can bind with calcium and mask the changes of the ionized calcium levels among critically ill participants, albumin-corrected calcium levels were calculated using the formula: albumin-corrected serum calcium (mg/dL) = serum total calcium (mg/dL) + 0.8 * (4(g/dL) - serum albumin (g/dL)) [27]. Survey-designed multinomial and binomial logistic regressions were performed to evaluate the associations of albumin-corrected calcium with CKM stages and Cox proportional hazards models were applied to investigate its association with CKM-cause and all-cause mortality.* (**Materials and Methods section**, **lines 20-26, page 8**).

*[Reference]*

*27.	Figge J, Jabor A, Kazda A, Fencl V. Anion Gap and Hypoalbuminemia. Crit Care Med (1998) 26(11):1807-10. doi: 10.1097/00003246-199811000-00019.*

*Survey-weighted multinomial logistic regression results for associations of albumin-corrected calcium revealed that albumin-corrected calcium was positively associated with the likelihood of being classified into stage 2-4b, referring to stage 0 (****Table S10****). Also, a positive association of albumin-corrected calcium with the likelihood of being classified into advanced CKM stages (****Table S11****) and with all-cause mortality (Table* ***S12****).* (**Results section**, **lines 1-5, page 11**).

***Table S10.*** *Survey-weighted multinomial logistic regression results for associations between ALP, albumin-corrected calcium, Phosphorus levels, and CKM stages 0-4b.*

*Adjusted by Age (years), Race and ethnicity, Poverty income ratio (PIR), Sex, BMI, Smoking status, Education, and vitamin D level, ALT, AST.*

*Abbreviations: ORs, odds ratios; 95%CI, 95% confidence interval; CKM, Cardiovascular-Kidney-Metabolic Syndrome; BMI, body mass index; ALT, Alanine transaminase; Aspartate aminotransferase, AST.*

***Table S11.*** *The associations of ALP quartiles, albumin-corrected calcium level, phosphorus level, and the likelihood of being classified into the advanced CKM stages.*

*Model 1: only ALP quartiles, Calcium (mg/dL), and Phosphorus (mg/dL), without adjustment.*

*Model 2: Model 1, adjusted by Age (years), Race and ethnicity, Poverty income ratio (PIR), Sex, BMI, Smoking status, Education, and vitamin D level, ALT, Alanine transaminase; Aspartate aminotransferase, AST.*

*Abbreviations: ORs, odds ratios; 95%CI, 95% confidence interval; CKM, Cardiovascular-Kidney-Metabolic Syndrome; BMI, body mass index.*

*Our subsequent results using albumin-corrected calcium [43] instead of total calcium (****Table S10-S12****) exhibited positive associations of albumin-corrected calcium both with CKM stage 2-4b and all-cause mortality, documenting the rationality of the explanation.* (**Discussion section**, **lines 4-7, page 15**)

*[Reference]*

*43.	Qi H, Wang B, Zhu L. Independent associations of serum calcium with or without albumin adjustment and serum phosphorus with nonalcoholic fatty liver disease: results from NHANES 1999-2018. 2024. Front Endocrinol (Lausanne) 15: 1323990*

**Phosphorus: contradictory associations across analyses**

**Comment:** Phosphorus is associated with stage 3 and 4b in multinomial models, not associated with advanced CKM after adjustment, and strongly associated with mortality. This pattern suggests context-specific effects, primarily driven by CKD severity. Thus, explicitly frame phosphorus as a late-stage CKD marker rather than a general CKM biomarker and emphasize that phosphorus may be more relevant to mortality risk than CKM stage classification.

**Response:** We sincerely thank your insight into the phosphorus and CKD. According to your suggestions, we underscored the role of phosphorus in CKD rather than a general CKM biomarker.

In the revised manuscript, we also pointed this out as follows:

*Similarly, a significant increase in the likelihood of being classified into odds of stage 3 (RRR: 2.85; 95% CI: 1.77, 4.59; p < 0.001) and stage 4b (RRR: 1.78; 95% CI: 1.37, 2.31; p < 0.001) was associated with higher phosphorus level, indicating its role in late-stage CKD-driven CKM.* (**Results section, lines 3-6, page 10**).

*To put together, our current data suggested that serum phosphorus levels served as a late-stage CKD marker and CKD-driven CKM rather than a general CKM biomarker. Notably, the COX analysis results of phosphorus indicated that phosphorus may be more relevant to mortality risk than CKM stage classification.* (**Discussion section, lines 15-19, page 16**).

**Mendelian randomization: instrument selection and interpretation**

**Comment:** The MR analysis is a strength, but removing >80% of ALP-associated SNPs raises concerns about instrument strength and selection bias. The MR-Egger results are largely non-significant. The authors are recommended to provide F-statistics or mean F-statistics to demonstrate instrument strength.

**Response:** Thank you for the helpful suggestion. We filtered the SNPs with the LDlink tool (https://ldlink.nih.gov/?tab=home), developed by the National Institutes of Health, was used to further exclude SNPs associated with known confounders (e.g., BMI, smoking status, education level) or outcomes (e.g., CVD, diabetes). Since CKM included several outcomes and shared risk factors, these might be the reason why we excluded so many SNPs.

Follow your suggestions, we provided the mean *F*-statistic of ALP in the revised manuscript, and added the *F*-statistics of included SNPs in **Table S3**.

*Detailed exclusion reasons are presented in* ***Table S3****, and 77 selected SNPs were adopted for further analysis, and their mean F-statistic was 87.53 (ranging from 28.24 to 589.78).* (**Materials and Methods section, lines 15-17, page 7**)

**Comment:** discuss the discrepancy between IVW and MR-Egger results more cautiously and avoid strong causal language where MR evidence is borderline or inconsistent.

**Response:** We apologize for the inaccurate description of the MR results. Now we have extensively revised the causal relationship with the ‘potential relationship’ to increase the scientific accuracy.

**Minor Comments**

**Comment:** Several sentences require grammatical polishing (e.g., “To addressing this gap,” “This was because the relative risk ratios…”).

**Response:** Thank you for pointing this out! To address this point, based on your comments, we have made the grammatical corrections throughout the manuscript.

**Comment:** Use consistent terminology for: “advanced CKM stages” vs. “advanced CKM”, “CKM-cause mortality” vs. “CKM-related mortality”.

**Response:** We are grateful for the your detailed advice, which has helped us refine our manuscript. Based on your comments, we now consistently use “advanced CKM stages” and “CKM-cause mortality” in the revised manuscript.

**Comment:** Clearly state whether calcium and phosphorus RRRs are per 1 mmol/L, 1 mg/dL, or per SD increase.

**Response:** Thank you for the practical suggestions. We have clarified that calcium and phosphorus levels were only analyzed as continuous variables (mg/dL) through all the models in the revised manuscript.

*Calcium and phosphorus levels were only analyzed as continuous variables (mg/dL) through all the models.* (**Materials and Methods section, lines 32-33, page 5**)

**Comment:** Ensure consistent use of OR vs. RRR vs. HR.

**Response:** We deeply appreciate the reviewer's thoughtful comment. We have extensively checked the use of OR vs. RRR vs. HR. to keep the consistency of each section.

**Comment:** Figures showing RCS curves should clearly label reference values.

**Response:** Thanks for the reviewer’s suggestion concerning reference values labelling in RCS curves. Lines have been inserted to point out reference values in RCS curves.

**Strengths of the Study.**

**Comment:**

- Large, nationally representative sample.
- Comprehensive CKM staging framework.
- Robust survey-weighted statistical methods.
- Integration of mortality analysis.
- Use of Mendelian randomization to explore causality.
- Clear evidence supporting ALP as a consistent CKM-associated biomarker.

**Response:** We are honored by the reviewer's appreciation for the strengths of our analysis, which we have further enhanced in response to your professional feedback.
