# Supplementary material for: Stage-specific associations of mineralization markers with CKM syndrome: Nationwide survey and genetic evidence for Alkaline phosphatase’s unique clinical role
Source: PLoS One. 2026 Jun 18;21(6):e0351946. doi: 10.1371/journal.pone.0351946 (PMC13278675; doi:10.1371/journal.pone.0351946)
Supplement: Attachment — Submitted filename: Response to reviewers_2.docx [file pone.0351946.s031.docx]

Dear Editor,

We are submitting a revised version of our manuscript (Manuscript ID: PONE-D-25-45261), ‘Stage-Specific Associations of Mineralization Markers with CKM Syndrome: Nationwide Survey and Genetic Evidence for Alkaline Phosphatase's Unique Clinical Role’, for further consideration at ***PLOS One***.

Understanding the mechanisms of Cardiovascular-Kidney-Metabolic (CKM) syndrome in developing novel diagnostic markers and therapeutic strategies, but the role of calcification markers in CKM stages remains unclear. Our manuscript shows that ALP may serve as a marker for CKM syndrome. While calcium showed stage-specific associations and phosphorus was less informative, ALP demonstrated consistent linear relationships across CKM stages. The reviewers responded that this study is “statistically sophisticated” and also “potentially clinically relevant”, some refinements should be addressed.

We revised our data analysis and clarification, addressed (where possible) all reviewer comments, and our revised manuscript is substantially stronger after completing this round of revisions. In this revised version for Reviewer 2#, changes to our manuscript were all highlighted using track changes. All Reviewer comments have been addressed and are outlined in detail in our Response to Reviewer and attached as a Word file.

We hope our work is of sufficiently high quality to warrant further consideration for publication in ***PLOS One***.

Thank you for serving as the editor for our manuscript, and please feel free to contact me with any questions.

Sincerely yours,

Dr. Liangru Shen

**Reviewer #2:**

**Major Comments**

**Comment 1:** The claim that ALP is more robust than calcium or phosphorus is not yet methodologically fair. ALP is modeled as quartiles, whereas calcium and phosphorus are modeled as continuous variables in mg/dL. Statistical significance across these different parameterizations is not directly comparable. The conclusion that ALP has a “unique clinical role” may therefore reflect modeling choices rather than true biological superiority. A stronger comparison would analyze all three markers on the same scale, such as z-scores, quartiles, or restricted spline models.

**Response:** Thank you for your professional and practical suggestions on the data comparison!! This is really a great idea! Therefore, to improve the comparison for the analysis, we standardized ALP, calcium, and phosphorus levels using z-scores and performed both survey-weighted multinomial logistic regression and survey-weighted modified Poisson regression models. The added results of using standardized data in regressions are updated in **Table 4** and **Table 6**.

Consistent with the conclusions from the unscaled data, per 1-SD increase of ALP was significantly positively associated with CKM stage 2-4b (all *p-values* < *0.001*), while associations of per 1-SD increase in calcium and phosphorus were weaker with CKM stage 3, or stage 4a in survey-weighted multinomial logistic regressions (**Table 4**).

***Table 4.*** *Survey-weighted multinomial logistic regression results for associations of scaled ALP, Calcium, and Phosphorus levels with CKM stages 0-4b.*

*Adjustment: Age (years), Race and ethnicity, Poverty income ratio, Sex, BMI, Smoking status, Education, and vitamin D level.*

*Abbreviations: RRRs, relative risk ratios; 95%CI, 95 confidence interval; CKM, Cardiovascular-Kidney-Metabolic Syndrome; BMI, body mass index.*

Survey-weighted modified Poisson regression models (**Table 6**) unveiled that per 1-SD increase in ALP and calcium were both significantly associated with advanced CKM stages in Model 1 and Model 2, but the association of per 1-SD increase in phosphorus with advanced CKM stages was not significant after adjustment.

***Table 6.*** *Survey-weighted modified Poisson regression for associations of scaled ALP, Calcium, and Phosphorus levels, with advanced CKM stages.*

*Model 1: only ALP (per 1-SD increase), Calcium (per 1-SD increase), and Phosphorus (per 1-SD increase), without adjustment.*

*Model 2: Model 1, adjusted by Age (years), Race and ethnicity, Poverty income ratio, Sex, BMI, Smoking status, Education, and vitamin D level.*

*Abbreviations: ORs, odds ratios; 95%CI, 95% confidence interval; CKM, Cardiovascular-Kidney-Metabolic Syndrome; BMI, body mass index.*

In summary, analyses of the three standardized markers strengthened our current conclusion that ALP showed a more stable and consistent association across CKM stages.

We also described the updated analysis in the revised manuscript:

*To improve comparability, regression analyses were also performed with all three markers standardized as z-scores.* (**Materials and Methods section, lines 3-4, page 8**).

*In the survey-weighted multinomial logistic regressions of standardized three markers, the associations between per 1-SD increase of ALP and CKM stages 2-4b remained significantly positive, whereas the associations for per 1-SD increase of calcium and phosphorus were weaker (****Table 4****), yielding findings consistent with those from unscaled analyses.* (**Results section, lines 5-8, page 10**).

*Also, in regression models standardizing the three markers, per 1-SD increase of ALP and calcium were positively associated with the advanced CKM stages in both models while association of per 1-SD increase of phosphorus with advanced CKM stages were not significant after adjustment (****Table 6****).* (**Results section, lines 28-31, page 10**).

**Comment 2:** Although sensitivity analyses (liver disease exclusion, ALT/AST adjustment, and albumin-corrected calcium) are commendable, residual confounding remains a concern. Key unadjusted factors, such as parathyroid hormone, fibroblast growth factor-23, phosphate binder use, and vitamin D supplement use, directly influence mineral metabolism and CKM progression.

**Response:** Thank you for your kind and constructive suggestions to improve the quality of our manuscript.

We agree that additional factors related to mineral metabolism may confound the associations between ALP, calcium, phosphorus, and CKM stages. To address this concern, we performed an additional sensitivity analysis by further adjusting for vitamin D supplement use and phosphate binder use where available in NHANES. The associations of ALP, calcium, and phosphorus with CKM stages remained substantially unchanged after these additional adjustments (**Table S10-S11**), suggesting that the main findings were stable.

***Table S10.*** *Survey-weighted multinomial logistic regression results for associations of ALP Quartile, Calcium, and Phosphorus levels with CKM stages 0-4b with the further adjustment of ALT, AST, vitamin D supplement intake, and phosphate binder use.*

*Adjustment: Age (years), Race and ethnicity, Poverty income ratio, Sex, BMI, Smoking status, Education, vitamin D level, ALT, AST, vitamin D supplement intake, and phosphate binder use.*

*Abbreviations: RRRs, relative risk ratios; 95%CI, 95 confidence interval; CKM, Cardiovascular-Kidney-Metabolic Syndrome; BMI, body mass index; ALT, Alanine transaminase; Aspartate aminotransferase, AST.*

***Table S11.*** *The associations of ALP quartiles, albumin-corrected calcium level, phosphorus level, and the likelihood of being classified into the advanced CKM stages with the further adjustment of ALT, AST, vitamin D supplement intake, and phosphate binder use.*

*Model 1: only ALP quartiles, Calcium (mg/dL), and Phosphorus (mg/dL), without adjustment.*

*Model 2: Model 1, adjusted by Age (years), Race and ethnicity, Poverty income ratio, Sex, BMI, Smoking status, Education, vitamin D level, ALT, AST, vitamin D supplement intake, and phosphate binder use.*

*Abbreviations: ORs, odds ratios; 95%CI, 95% confidence interval; CKM, Cardiovascular-Kidney-Metabolic Syndrome; BMI, body mass index; ALT, Alanine transaminase; Aspartate aminotransferase, AST.*

However, parathyroid hormone and fibroblast growth factor-23 were not available in the public NHANES files for the study cycles included in our analysis. Therefore, we were unable to adjust for these biomarkers. We have acknowledged this issue in the revised limitation section and further research with complete data should be performed in the future:

*Other residual confounders related to mineral metabolism, such as parathyroid hormone, fibroblast growth factor-23, were not included in the adjustment due to absence of data. Hence, further research is needed to extend the analysis and confirm the role of ALP in CKM syndrome.* (**Discussion section, lines 14-17, page 17**).

**Comment 3:** The NHANES analysis is based on a multi-ethnic U.S. population including Mexican Americans, Non-Hispanic Blacks, and multiracial groups. However, the MR analysis exclusively uses summary statistics from individuals of European ancestry (UK Biobank and FinnGen). Genetic variants (SNPs) associated with ALP levels can vary significantly across ethnicities. Applying European-derived genetic instruments to interpret findings from a diverse U.S. cohort may limit the validity of the causal inferences made in the conclusion.

**Response:** We really appreciated the reviewer for this comment. We agreed that the ancestry mismatch between the observational NHANES analysis and the MR analysis may limit the interpretation of causal inference in NHANES results. However, the summary statistics from the multi-ethnic population were not available for us currently. Therefore, we described this limitation in the discussion section as below:

*Given that the MR analysis relied on European-ancestry summary statistics, while NHANES included participants from diverse racial and ethnic backgrounds, the causal inferences from MR analysis here may not fully generalize to NHANES population and explain the conclusions in NHANES data.* (**Discussion section, lines 4-8, page 17**).
