# Supplementary material for: Stage-specific associations of mineralization markers with CKM syndrome: Nationwide survey and genetic evidence for Alkaline phosphatase’s unique clinical role
Source: PLoS One. 2026 Jun 18;21(6):e0351946. doi: 10.1371/journal.pone.0351946 (PMC13278675; doi:10.1371/journal.pone.0351946)
Supplement: Peer review history

# Peer review: Decision Letter 0

**DOI:** 10.1371/journal.pone.0351946.r001

**Authors:** Johanna Pruller

27 Jan 2026

PONE-D-25-45261

Stage-Specific Associations of Mineralization Markers with CKM Syndrome: Nationwide Survey and Genetic Evidence for Alkaline Phosphatase’s Unique Clinical Role

PLOS One

Dear Dr. Shen,

Thank you for submitting your manuscript to PLOS ONE. After careful consideration, we feel that it has merit but does not fully meet PLOS ONE’s publication criteria as it currently stands. Therefore, we invite you to submit a revised version of the manuscript that addresses the points raised during the review process.

Please submit your revised manuscript by Mar 13 2026 11:59PM. If you will need more time than this to complete your revisions, please reply to this message or contact the journal office at plosone@plos.org. When you’re ready to submit your revision, log on to https://www.editorialmanager.com/pone/ and select the ‘Submissions Needing Revision’ folder to locate your manuscript file.

Please include the following items when submitting your revised manuscript:

- A letter that responds to each point raised by the academic editor and reviewer(s). You should upload this letter as a separate file labeled ‘Response to Reviewers’.
- A marked-up copy of your manuscript that highlights changes made to the original version. You should upload this as a separate file labeled ‘Revised Manuscript with Track Changes’.
- An unmarked version of your revised paper without tracked changes. You should upload this as a separate file labeled ‘Manuscript’.

If you would like to make changes to your financial disclosure, please include your updated statement in your cover letter. Guidelines for resubmitting your figure files are available below the reviewer comments at the end of this letter.

If applicable, we recommend that you deposit your laboratory protocols in protocols.io to enhance the reproducibility of your results. Protocols.io assigns your protocol its own identifier (DOI) so that it can be cited independently in the future. For instructions see: https://journals.plos.org/plosone/s/submission-guidelines#loc-laboratory-protocols. Additionally, PLOS ONE offers an option for publishing peer-reviewed Lab Protocol articles, which describe protocols hosted on protocols.io. Read more information on sharing protocols at https://plos.org/protocols?utm_medium=editorial-email&utm_source=authorletters&utm_campaign=protocols.

We look forward to receiving your revised manuscript.

Kind regards,

Johanna Pruller, Ph.D.

Senior Editor

PLOS One

Journal Requirements:

When submitting your revision, we need you to address these additional requirements.

1.Please ensure that your manuscript meets PLOS ONE’s style requirements, including those for file naming. The PLOS ONE style templates can be found at

https://journals.plos.org/plosone/s/file?id=wjVg/PLOSOne_formatting_sample_main_body.pdf and

https://journals.plos.org/plosone/s/file?id=ba62/PLOSOne_formatting_sample_title_authors_affiliations.pdf

2. Thank you for stating the following financial disclosure:

“This study received support from the National Natural Science Foundation of China [grant number 82300346], the Natural Science Foundation of Suqian [Grant Number 2021J296].”

Please state what role the funders took in the study.  If the funders had no role, please state: "The funders had no role in study design, data collection and analysis, decision to publish, or preparation of the manuscript."

If this statement is not correct you must amend it as needed.

Please include this amended Role of Funder statement in your cover letter; we will change the online submission form on your behalf.

3. Please note that funding information should not appear in any section or other areas of your manuscript. We will only publish funding information present in the Funding Statement section of the online submission form. Please remove any funding-related text from the manuscript.

4. Your ethics statement should only appear in the Methods section of your manuscript. If your ethics statement is written in any section besides the Methods, please move it to the Methods section and delete it from any other section. Please ensure that your ethics statement is included in your manuscript, as the ethics statement entered into the online submission form will not be published alongside your manuscript.

5. Please include captions for your Supporting Information files at the end of your manuscript, and update any in-text citations to match accordingly. Please see our Supporting Information guidelines for more information: http://journals.plos.org/plosone/s/supporting-information.

6. If the reviewer comments include a recommendation to cite specific previously published works, please review and evaluate these publications to determine whether they are relevant and should be cited. There is no requirement to cite these works unless the editor has indicated otherwise.

Additional Editor Comments:

Please note that we have only been able to secure a single reviewer to assess your manuscript. We are issuing a decision on your manuscript at this point to prevent further delays in the evaluation of your manuscript. Please be aware that the editor who handles your revised manuscript might find it necessary to invite additional reviewers to assess this work once the revised manuscript is submitted. However, we will aim to proceed on the basis of this single review if possible.

Could you please revise the manuscript to carefully address the concerns raised?

[Note: HTML markup is below. Please do not edit.]

Reviewer’s Responses to Questions

**Comments to the Author**

1. Is the manuscript technically sound, and do the data support the conclusions?

The manuscript must describe a technically sound piece of scientific research with data that supports the conclusions. Experiments must have been conducted rigorously, with appropriate controls, replication, and sample sizes. The conclusions must be drawn appropriately based on the data presented.

Reviewer #1: Yes

**********

2. Has the statistical analysis been performed appropriately and rigorously?

Reviewer #1: Yes

**********

3. Have the authors made all data underlying the findings in their manuscript fully available?

The PLOS Data policy requires authors to make all data underlying the findings described in their manuscript fully available without restriction, with rare exception (please refer to the Data Availability Statement in the manuscript PDF file). The data should be provided as part of the manuscript or its supporting information, or deposited to a public repository. For example, in addition to summary statistics, the data points behind means, medians and variance measures should be available. If there are restrictions on publicly sharing data—e.g. participant privacy or use of data from a third party—those must be specified.

Reviewer #1: Yes

**********

4. Is the manuscript presented in an intelligible fashion and written in standard English?

PLOS ONE does not copyedit accepted manuscripts, so the language in submitted articles must be clear, correct, and unambiguous. Any typographical or grammatical errors should be corrected at revision, so please note any specific errors here.

Reviewer #1: Yes

**********

5. Review Comments to the Author

Please use the space provided to explain your answers to the questions above. You may also include additional comments for the author, including concerns about dual publication, research ethics, or publication ethics. (Please upload your review as an attachment if it exceeds 20,000 characters)

Reviewer #1: Dear the Editor of the PLOS ONE Journal,

Thank you very much for providing me with this opportunity. I would also like to sincerely acknowledge the authors for their valuable contributions to the scientific community and for advancing knowledge in their respective field.

Manuscript Number: PONE-D-25-45261

Article Type: Research Article

Title: Stage-Specific Associations of Mineralization Markers with CKM Syndrome: Nationwide Survey and Genetic Evidence for Alkaline Phosphatase’s Unique Clinical Role

• My overall evaluation of the manuscript is that the manuscript addresses an important and timely topic: the role of mineralization markers (ALP, calcium, and phosphorus) across stages of Cardiovascular–Kidney–Metabolic (CKM) syndrome using a large, nationally representative dataset (NHANES), complemented by Mendelian randomization analyses. The combination of stage-specific epidemiological analysis, mortality outcomes, and genetic causal inference is a notable strength. The study is generally well designed and statistically sophisticated, and the findings particularly the consistent association of ALP with CKM stages and mortality are potentially clinically relevant. However, several methodological clarifications, interpretational refinements, and presentation improvements are required to strengthen the scientific validity and transparency of the work.

Major Comments

Conceptual clarity of CKM staging and causal interpretation

• The manuscript applies modified AHA CKM staging using NHANES variables, which is reasonable; however, CKM stages are composite constructs incorporating outcomes (CVD, CKD, diabetes) that are also biologically linked to ALP, calcium, and phosphorus. This creates a risk of circularity or over interpretation, particularly when associations are described as “risk” of CKM stages in a cross-sectional design. What I recommend is that the authors are expected to clarify throughout manuscript that the primary analyses are associational, not predictive and replace phrasing such as “risk of CKM stage” with “likelihood of being classified into CKM stage” where appropriate and explicitly discuss potential bidirectional relationships between ALP and CKM components in the discussion.

Study participants

• Participants under 20 years of age or those who were pregnant were excluded. Additionally, individuals with insufficient data to evaluate CKM staging were excluded. According to WHO, age of 18 years and above is considered as an adult, what is your justification to exclude participants under 20 years old? In addition, individuals with insufficient data were excluded from the study. What percentage or which data level was considered insufficient?

Cross-sectional design vs. causal inference

• While the Mendelian randomization (MR) analysis strengthens causal inference for ALP CVD and CKD ALP relationships, the NHANES analyses remain cross-sectional. Some interpretations in the discussion imply disease progression or temporality that cannot be established. Therefore, clearly distinguish between cross-sectional NHANES findings (association), prospective mortality analyses (temporal but limited), and MR findings (genetic causality) and avoid implying that ALP “drives progression” of CKM stages without longitudinal CKM staging data.

Use and interpretation of ALP as a non-specific biomarker

• Total serum ALP includes hepatic, bone, intestinal, and placental isoenzymes, yet the manuscript interprets ALP largely as a calcification/mineralization marker. Liver disease, cholestasis, and subclinical hepatic dysfunction may confound associations. Thus, explicitly acknowledge the non-specific nature of total ALP and discuss potential confounding from liver-related ALP sources. If available, clarify whether AST/ALT or liver disease variables were considered or why they were not included.

Calcium findings: inconsistency across stages and outcomes

• Calcium shows positive association with advanced CKM stages, non-linear associations in RCS models, and inverse association with all-cause mortality. These findings appear biologically inconsistent and require clearer explanation. Therefore, expand discussion on tight homeostatic regulation of serum calcium, influence of albumin (uncorrected calcium limitation), CKD-related hypocalcemia vs. CVD-related hypercalcemia and clarify whether albumin-corrected calcium was considered and justify its exclusion.

Phosphorus: contradictory associations across analyses

• Phosphorus is associated with stage 3 and 4b in multinomial models, not associated with advanced CKM after adjustment, and strongly associated with mortality. This pattern suggests context-specific effects, primarily driven by CKD severity. Thus, explicitly frame phosphorus as a late-stage CKD marker rather than a general CKM biomarker and emphasize that phosphorus may be more relevant to mortality risk than CKM stage classification.

Mendelian randomization: instrument selection and interpretation

• The MR analysis is a strength, but removing >80% of ALP-associated SNPs raises concerns about instrument strength and selection bias. The MR-Egger results are largely non-significant. The authors are recommended to provide F-statistics or mean F-statistics to demonstrate instrument strength, discuss the discrepancy between IVW and MR-Egger results more cautiously and avoid strong causal language where MR evidence is borderline or inconsistent.

Minor Comments

• Several sentences require grammatical polishing (e.g., “To addressing this gap,” “This was because the relative risk ratios…”).

• Use consistent terminology for: “advanced CKM stages” vs. “advanced CKM”, “CKM-cause mortality” vs. “CKM-related mortality”

• Clearly state whether calcium and phosphorus RRRs are per 1 mmol/L, 1 mg/dL, or per SD increase.

• Ensure consistent use of OR vs. RRR vs. HR.

• Figures showing RCS curves should clearly label reference values.

• Strengths of the Study

• Large, nationally representative sample.

• Comprehensive CKM staging framework.

• Robust survey-weighted statistical methods.

• Integration of mortality analysis.

• Use of Mendelian randomization to explore causality.

• Clear evidence supporting ALP as a consistent CKM-associated biomarker.

Overall Recommendation

Major revision

The manuscript is scientifically valuable and suitable for publication after conceptual clarification, cautious interpretation of causality, and improved discussion of biomarker specificity and biological mechanisms. Addressing the points above will significantly enhance the rigor, clarity, and impact of the study.

**********

6. PLOS authors have the option to publish the peer review history of their article (what does this mean?). If published, this will include your full peer review and any attached files.

If you choose “no”, your identity will remain anonymous but your review may still be made public.

**Do you want your identity to be public for this peer review?**    For information about this choice, including consent withdrawal, please see our Privacy Policy.

Reviewer #1: **Yes:**   Dr. Hussen Abdu Muhidin

**********

[NOTE: If reviewer comments were submitted as an attachment file, they will be attached to this email and accessible via the submission site. Please log into your account, locate the manuscript record, and check for the action link "View Attachments". If this link does not appear, there are no attachment files.]

To ensure your figures meet our technical requirements, please review our figure guidelines: https://journals.plos.org/plosone/s/figures

You may also use PLOS’s free figure tool, NAAS, to help you prepare publication quality figures: https://journals.plos.org/plosone/s/figures#loc-tools-for-figure-preparation.

NAAS will assess whether your figures meet our technical requirements by comparing each figure against our figure specifications.

Attachment Submitted filename: Comment for PLOS ONE 45261.docx

# Author comment: Author response to Decision Letter 1

**DOI:** 10.1371/journal.pone.0351946.r002

23 Feb 2026

Dear reviewer,

We are submitting a revised version of our manuscript (Manuscript ID: PONE-D-25-45261), ‘Stage-Specific Associations of Mineralization Markers with CKM Syndrome: Nationwide Survey and Genetic Evidence for Alkaline Phosphatase’s Unique Clinical Role’, for further consideration at PLOS One.

Understanding the mechanisms of Cardiovascular-Kidney-Metabolic (CKM) syndrome in developing novel diagnostic markers and therapeutic strategies, but the role of calcification markers in CKM stages remains unclear. Our manuscript shows that ALP may serve as a marker for CKM syndrome. While calcium showed stage-specific associations and phosphorus was less informative, ALP demonstrated consistent linear relationships across CKM stages. You responded that this study is “statistically sophisticated” and also “potentially clinically relevant”, but some refinements should be addressed.

We revised our data analysis and clarified the methodological description, addressed (where possible) all your comments, and our revised manuscript is substantially stronger after completing this round of revisions. In this revised version, changes to our manuscript were all highlighted using track changes. All your comments have been addressed and are outlined in detail in our Response to Reviewer and attached as a Word file.

We hope our work is of sufficiently high quality to warrant further consideration for publication in PLOS One.

Thank you for serving as the reviewer for our manuscript, and please feel free to contact me with any questions.

Sincerely yours,

Dr. Liangru Shen

Attachment Submitted filename: Response to reviewers.docx

# Peer review: Decision Letter 1

**DOI:** 10.1371/journal.pone.0351946.r003

**Authors:** Rashmi Supriya

19 May 2026

PONE-D-25-45261R1

Stage-Specific Associations of Mineralization Markers with CKM Syndrome: Nationwide Survey and Genetic Evidence for Alkaline Phosphatase’s Unique Clinical Role

PLOS One

Dear Dr. Shen,

Thank you for submitting your manuscript to PLOS ONE. After careful consideration, we feel that it has merit but does not fully meet PLOS ONE’s publication criteria as it currently stands. Therefore, we invite you to submit a revised version of the manuscript that addresses the points raised during the review process.

Please submit your revised manuscript by Jul 03 2026 11:59PM. If you will need more time than this to complete your revisions, please reply to this message or contact the journal office at plosone@plos.org. When you’re ready to submit your revision, log on to https://www.editorialmanager.com/pone/ and select the ‘Submissions Needing Revision’ folder to locate your manuscript file.

Please include the following items when submitting your revised manuscript:

- A letter that responds to each point raised by the academic editor and reviewer(s). You should upload this letter as a separate file labeled ‘Response to Reviewers’.
- A marked-up copy of your manuscript that highlights changes made to the original version. You should upload this as a separate file labeled ‘Revised Manuscript with Track Changes’.
- An unmarked version of your revised paper without tracked changes. You should upload this as a separate file labeled ‘Manuscript’.

If you would like to make changes to your financial disclosure, please include your updated statement in your cover letter. Guidelines for resubmitting your figure files are available below the reviewer comments at the end of this letter.

If applicable, we recommend that you deposit your laboratory protocols in protocols.io to enhance the reproducibility of your results. Protocols.io assigns your protocol its own identifier (DOI) so that it can be cited independently in the future. For instructions see: https://journals.plos.org/plosone/s/submission-guidelines#loc-laboratory-protocols. Additionally, PLOS ONE offers an option for publishing peer-reviewed Lab Protocol articles, which describe protocols hosted on protocols.io. Read more information on sharing protocols at https://plos.org/protocols?utm_medium=editorial-email&utm_source=authorletters&utm_campaign=protocols.

As the corresponding author, your ORCID iD is verified in the submission system and will appear in the published article. PLOS supports the use of ORCID, and we encourage all coauthors to register for an ORCID iD and use it as well. Please encourage your coauthors to verify their ORCID iD within the submission system before final acceptance, as unverified ORCID iDs will not appear in the published article. *Only*    the individual author can complete the verification step; PLOS staff *cannot*    verify ORCID iDs on behalf of authors.

We look forward to receiving your revised manuscript.

Kind regards,

Rashmi Supriya

Academic Editor

PLOS One

**Journal Requirements:**

1. If the reviewer comments include a recommendation to cite specific previously published works, please review and evaluate these publications to determine whether they are relevant and should be cited. There is no requirement to cite these works unless the editor has indicated otherwise.

2. Please review your reference list to ensure that it is complete and correct. If you have cited papers that have been retracted, please include the rationale for doing so in the manuscript text, or remove these references and replace them with relevant current references. Any changes to the reference list should be mentioned in the rebuttal letter that accompanies your revised manuscript. If you need to cite a retracted article, indicate the article’s retracted status in the References list and also include a citation and full reference for the retraction notice.

[Note: HTML markup is below. Please do not edit.]

Reviewers’ comments:

Reviewer’s Responses to Questions

**Comments to the Author**

1. If the authors have adequately addressed your comments raised in a previous round of review and you feel that this manuscript is now acceptable for publication, you may indicate that here to bypass the “Comments to the Author” section, enter your conflict of interest statement in the “Confidential to Editor” section, and submit your "Accept" recommendation.

Reviewer #2: All comments have been addressed

**********

2. Is the manuscript technically sound, and do the data support the conclusions?

The manuscript must describe a technically sound piece of scientific research with data that supports the conclusions. Experiments must have been conducted rigorously, with appropriate controls, replication, and sample sizes. The conclusions must be drawn appropriately based on the data presented.

Reviewer #2: Yes

**********

3. Has the statistical analysis been performed appropriately and rigorously?

Reviewer #2: Yes

**********

4. Have the authors made all data underlying the findings in their manuscript fully available?

The PLOS Data policy requires authors to make all data underlying the findings described in their manuscript fully available without restriction, with rare exception (please refer to the Data Availability Statement in the manuscript PDF file). The data should be provided as part of the manuscript or its supporting information, or deposited to a public repository. For example, in addition to summary statistics, the data points behind means, medians and variance measures should be available. If there are restrictions on publicly sharing data—e.g. participant privacy or use of data from a third party—those must be specified.

Reviewer #2: Yes

**********

5. Is the manuscript presented in an intelligible fashion and written in standard English?

PLOS ONE does not copyedit accepted manuscripts, so the language in submitted articles must be clear, correct, and unambiguous. Any typographical or grammatical errors should be corrected at revision, so please note any specific errors here.

Reviewer #2: Yes

**********

6. Review Comments to the Author

Please use the space provided to explain your answers to the questions above. You may also include additional comments for the author, including concerns about dual publication, research ethics, or publication ethics. (Please upload your review as an attachment if it exceeds 20,000 characters)

Reviewer #2: 1. The claim that ALP is more robust than calcium or phosphorus is not yet methodologically fair. ALP is modeled as quartiles, whereas calcium and phosphorus are modeled as continuous variables in mg/dL. Statistical significance across these different parameterizations is not directly comparable. The conclusion that ALP has a “unique clinical role” may therefore reflect modeling choices rather than true biological superiority. A stronger comparison would analyze all three markers on the same scale, such as z-scores, quartiles, or restricted spline models.

2. Although sensitivity analyses (liver disease exclusion, ALT/AST adjustment, and albumin-corrected calcium) are commendable, residual confounding remains a concern. Key unadjusted factors, such as parathyroid hormone, fibroblast growth factor-23, phosphate binder use, and vitamin D supplement use, directly influence mineral metabolism and CKM progression.

3. The NHANES analysis is based on a multi-ethnic U.S. population including Mexican Americans, Non-Hispanic Blacks, and multiracial groups. However, the MR analysis exclusively uses summary statistics from individuals of European ancestry (UK Biobank and FinnGen). Genetic variants (SNPs) associated with ALP levels can vary significantly across ethnicities. Applying European-derived genetic instruments to interpret findings from a diverse U.S. cohort may limit the validity of the causal inferences made in the conclusion.

**********

7. PLOS authors have the option to publish the peer review history of their article (what does this mean?). If published, this will include your full peer review and any attached files.

If you choose “no”, your identity will remain anonymous but your review may still be made public.

**Do you want your identity to be public for this peer review?**    For information about this choice, including consent withdrawal, please see our Privacy Policy.

Reviewer #2: No

**********

[NOTE: If reviewer comments were submitted as an attachment file, they will be attached to this email and accessible via the submission site. Please log into your account, locate the manuscript record, and check for the action link "View Attachments". If this link does not appear, there are no attachment files.]

To ensure your figures meet our technical requirements, please review our figure guidelines: https://journals.plos.org/plosone/s/figures

You may also use PLOS’s free figure tool, NAAS, to help you prepare publication quality figures: https://journals.plos.org/plosone/s/figures#loc-tools-for-figure-preparation.

NAAS will assess whether your figures meet our technical requirements by comparing each figure against our figure specifications.

# Author comment: Author response to Decision Letter 2

**DOI:** 10.1371/journal.pone.0351946.r004

26 May 2026

Dear reviewer 2#:

Thanks very much for your devotions in reviewing our manuscript! We revised our data analysis and clarification, addressed (where possible) your comments, and our revised manuscript is substantially stronger after completing this round of revisions. In this revised version for Reviewer 2#, changes to our manuscript were all highlighted using track changes. All Reviewer comments have been addressed and are outlined in detail in our Response to Reviewer and attached as a Word file.

We hope our work is of sufficiently high quality to warrant further consideration for publication in PLOS One.

Sincerely yours,

Dr. Liangru Shen

Attachment Submitted filename: Response to reviewers_2.docx

# Editor report: Decision Letter 2

**DOI:** 10.1371/journal.pone.0351946.r005

**Authors:** Rashmi Supriya

4 Jun 2026

Stage-Specific Associations of Mineralization Markers with CKM Syndrome: Nationwide Survey and Genetic Evidence for Alkaline Phosphatase’s Unique Clinical Role

PONE-D-25-45261R2

Dear Authors,

We’re pleased to inform you that your manuscript has been judged scientifically suitable for publication and will be formally accepted for publication once it meets all outstanding technical requirements.

Within one week, you’ll receive an e-mail detailing the required amendments. When these have been addressed, you’ll receive a formal acceptance letter and your manuscript will be scheduled for publication.

An invoice will be generated when your article is formally accepted. Please note, if your institution has a publishing partnership with PLOS and your article meets the relevant criteria, all or part of your publication costs will be covered. Please make sure your user information is up-to-date by logging into Editorial Manager at Editorial Manager® and clicking the ‘Update My Information’ link at the top of the page. For questions related to billing, please contact billing support.

If your institution or institutions have a press office, please notify them about your upcoming paper to help maximize its impact. If they’ll be preparing press materials, please inform our press team as soon as possible -- no later than 48 hours after receiving the formal acceptance. Your manuscript will remain under strict press embargo until 2 pm Eastern Time on the date of publication. For more information, please contact onepress@plos.org.

Kind regards,

Rashmi Supriya

Academic Editor

PLOS One

# Editor report: Acceptance letter

**DOI:** 10.1371/journal.pone.0351946.r006

**Authors:** Rashmi Supriya

PONE-D-25-45261R2

PLOS One

Dear Dr. Shen,

I’m pleased to inform you that your manuscript has been deemed suitable for publication in PLOS One. Congratulations! Your manuscript is now being handed over to our production team.

At this stage, our production department will prepare your paper for publication. This includes ensuring the following:

* All references, tables, and figures are properly cited

* All relevant supporting information is included in the manuscript submission,

* There are no issues that prevent the paper from being properly typeset

You will receive further instructions from the production team, including instructions on how to review your proof when it is ready. Please keep in mind that we are working through a large volume of accepted articles, so please give us a few days to review your paper and let you know the next and final steps.

Lastly, if your institution or institutions have a press office, please let them know about your upcoming paper now to help maximize its impact. If they’ll be preparing press materials, please inform our press team within the next 48 hours. Your manuscript will remain under strict press embargo until 2 pm Eastern Time on the date of publication. For more information, please contact onepress@plos.org.

You will receive an invoice from PLOS for your publication fee after your manuscript has reached the completed accept phase. If you receive an email requesting payment before acceptance or for any other service, this may be a phishing scheme. Learn how to identify phishing emails and protect your accounts at https://explore.plos.org/phishing.

If we can help with anything else, please email us at customercare@plos.org.

Thank you for submitting your work to PLOS ONE and supporting open access.

Kind regards,

PLOS ONE Editorial Office Staff

on behalf of

Dr. Rashmi Supriya

Academic Editor

PLOS One
